# Calcitriol promotes M2 polarization of tumor-associated macrophages in 4T1 mouse mammary gland cancer via the induction of proinflammatory cytokines

**DOI:** 10.1038/s41598-024-54433-x

**Published:** 2024-02-15

**Authors:** Martyna Stachowicz-Suhs, Natalia Łabędź, Artur Anisiewicz, Joanna Banach, Dagmara Kłopotowska, Magdalena Milczarek, Aleksandra Piotrowska, Piotr Dzięgiel, Adam Maciejczyk, Rafał Matkowski, Joanna Wietrzyk

**Affiliations:** 1 Department of Experimental Oncology, Hirszfeld Institute of Immunology and Experimental Therapy, Weigla 12, 53-114 Wroclaw, Poland; 2 Division of Histology and Embryology, Department of Human Morphology and Embryology, Faculty of Medicine, Wroclaw Medical University, Chałubińskiego 6a, 50-368 Wroclaw, Poland; 3 Department of Oncology, Wroclaw Medical University, Pl. Ludwika Hirszfelda 12, 53-413 Wrocław, Poland; 4 Lower Silesian Oncology, Pulmonology and Hematology Center, Pl. Ludwika Hirszfelda 12, 53-413 Wrocław, Poland

**Keywords:** Cancer, Cell biology, Immunology, Oncology

## Abstract

Our research found that vitamin D_3_ (VD_3_) treatment increased lung metastasis in mice with 4T1 murine breast cancer (BC). This study aims to investigate the impact of VD_3_ on the activation of tumor-associated macrophages (TAMs) in BC. Mice bearing 4T1, E0771, 67NR BC cells, and healthy mice, were fed diets with varying VD_3_ contents (100—deficient, 1000—normal, and 5000 IU/kg—elevated). Some mice in the 1000 and 100 IU/kg groups received calcitriol. We studied bone metastasis and characterized TAMs and bone marrow-derived macrophages (BMDMs). 4T1 cells had higher bone metastasis potential in the 5000 IU/kg and calcitriol groups. In the same mice, an elevated tumor osteopontin level and M2 polarization of TAMs (MHCII^low^ CD44^high^ phenotype) were observed. Gene expression analysis confirmed M2 polarization of 4T1 (but not 67NR) TAMs and BMDMs, particularly in the 100 IU + cal group (increased *Mrc1*, *Il23*, and *Il6*). This polarization was likely due to COX-2/PGE_2_ induction in 4T1 calcitriol-treated cells, leading to increased proinflammatory cytokines like IL-6 and IL-23. Future studies will explore COX-2/PGE_2_ as a primary mediator of calcitriol-stimulated inflammation in the BC microenvironment, especially relevant for BC patients with VD_3_ deficiency and supplementation.

## Introduction

In the early stages of tumor development, cancer cells orchestrate the requirement of various cell types to form the tumor microenvironment (TME). Within the TME, there are cells with both inhibitory and stimulatory roles in furthering tumor growth^1^. Notably, tumor-associated macrophages (TAMs) constitute a significant component of the TME, often accounting for up to 50% of the tumor cell composition. High TAM infiltration is associated with poor prognosis, particularly in breast cancer (BC)^2^. Macrophages, integral to the innate immune system, possess phagocytic capabilities and can present antigens, thereby modulating immune responses. Monocyte-derived macrophages encompass the following two distinct populations: M1 and M2. M1 macrophages, the so-called classical or inflammatory macrophages, are proficient at phagocytosis and cytotoxicity. Conversely, M2 macrophages, referred to as alternative or immunosuppressive macrophages, play a role in tissue repair. Both macrophage populations coexist within the TME. Cancer cells release various molecules, such as CC motif chemokine ligand 2 (CCL2), to recruit monocytes and macrophages to the primary tumor site, where they transform into TAMs^3,4^. Generally, M1 macrophages within the TME exhibit anticancer activity. However, as cancer progresses, M2-like macrophages with immunosuppressive properties become predominant. These cells promote primary tumor growth, enhance metastatic potential, and contribute to increased vascularization and remodeling of tumor stroma^5^.

Vitamin D_3_ (VD_3_), primarily acting through its hormonally active metabolite calcitriol (1,25(OH)_2_D_3_), is recognized as an immunomodulatory molecule^6^. Calcitriol is a ligand for vitamin D receptor (VDR), which forms a heterodimer with retinoid X receptor (RXR) after ligand binding, functioning as a transcription factor that regulates the expression of multiple genes^7^. VDR expression is widespread across various cell types, including cancer and immune cells^8^. Calcitriol’s impact on immune cells is often described as immunosuppressive, as it can shift the immune response from Th1 to Th2 and promote the expansion of Treg cells^9^. Calcitriol is also known to induce the differentiation of M2 macrophages^10^. Despite its immunomodulatory properties, calcitriol has been investigated for its potential anticancer effects. Apart from the direct inhibition of cancer cell proliferation^11,12^, these effects are attributed to the concurrent induction of inflammation often observed during cancer development, where calcitriol’s immunosuppressive properties could prove beneficial^12^. Numerous studies, including experimental^13–17^, epidemiological^18–20^, or clinical^21^, have suggested potential benefits of VD_3_ for cancer patients, including those with BC. However, some epidemiological studies have reported no significant effects of VD_3_ on the clinicopathological features of BC, and in some cases, adverse effects have been observed in patients with elevated levels of the VD_3_ metabolite 25(OH)D_3_ in their plasma^22,23^. In our previous investigations, we observed that calcitriol treatment of mice with 4T1 mouse mammary gland tumor led to increased plasma and tumor tissue levels of CCL2 and elevated arginase-1 (Arg-1) expression in tumor tissue^24^. Concurrently, we noted an increased metastatic potential of 4T1 cells in mice treated with calcitriol or its analogs^25,26^. Subsequent ex vivo studies on murine bone marrow-derived macrophages (BMDMs) exposed to calcitriol and differentiated into M0, M1, or M2 in the presence or absence of conditioned media (CM) from cancer 4T1, 67NR, and normal Eph4-Ev mouse mammary gland cells indicated that calcitriol enhanced the differentiation of M2 macrophages, particularly in the presence of CM from 4T1 metastatic cells. Moreover, M2 macrophages stimulated with calcitriol during differentiation were found to enhance the migration of 4T1 cells through fibronectin^27^. On the other hand, Zhang et al. cocultured VDR-overexpressing 4T1 cells with RAW 264.7 macrophages and demonstrated that VDR overexpression reduced the prometastatic effects of cocultured macrophages on 4T1 cancer cells and inhibited the induction of EMT in these cells^28^. In addition, a study involving E0771 mouse mammary gland tumors revealed that cholecalciferol (VD_3_) administered via gavage decreased tumor growth in normal mice and increased it in obese mice. This was correlated with decreased infiltration of tumor tissue by CD8^+^ T cells in obese mice and an increased level of these lymphocytes in normal mice. Moreover, the spleen and lymph nodes of normal mice bearing E0771 tumors and treated with cholecalciferol showed decreased infiltration by M1 (F4/80^+^ CD11b^+^) macrophages^29^.

It motivated us to investigate TAMs in mice bearing 4T1, E0771 metastatic, and 67NR non-metastatic mouse mammary gland tumors. These mice were subjected to varying levels of VD_3_ in their diet and treated with or without calcitriol^26^. Considering the prevalent VD_3_ deficiency among BC patients^30^, our experimental approach aimed to assess not only the impact of deficient or excessive 25(OH)D_3_ levels in plasma on mammary gland tumor development in mice but also whether administering calcitriol to an organism deficient in VD_3_ would yield the same effects as administering calcitriol to the body with normal levels of this vitamin^26^. We analyzed the phenotype of TAMs isolated from 4T1, 67NR, and E0771 tumors and examined the expression of genes associated with macrophage characteristics and VD_3_ action. Given that the observed effects of calcitriol on metastasis enhancement were specific for the 4T1 tumor model, we used CM from 4T1 cells treated or not with calcitriol to differentiate BMDMs from healthy mice being exposed to various VD_3_ diets and calcitriol treatment. Additionally, we investigated how calcitriol influences 4T1 cells in vitro within the context of factors associated with inflammation in BC. In light of the findings from our mouse experiments, we conducted an analysis of BC patients’ tumors, considering the expression of cyclooxygenase 2 (COX-2), VD_3_ metabolizing enzymes, VDR expression, as well as 25(OH)D_3_ plasma levels.

## Materials and methods

### Cancer cell lines culture

The 4T1 cell line was obtained from the American Type Culture Collection (ATCC, Rockville, MD, USA). These cells were cultured in RPMI 1640 Gluta-MAX (Gibco™, Thermo Fisher Scientific, Waltham, MA, USA), supplemented with 10% Foetal Bovine Serum (FBS; HyClone, GE Healthcare, Chicago, IL, USA), 1 mM sodium pyruvate, and 3.5 g/L glucose (both from Sigma-Aldrich, Saint Louis, MO, USA). The 67NR (non-metastatic isogenic to 4T1 cell line) was obtained from Barbara Ann Karmanos Cancer Institute, Detroit, MI, USA. These cells were cultured in Dulbecco’s modified Eagle medium (DMEM; Gibco, Scotland, UK) supplemented with 10% calf bovine serum (CBS; ATCC, Rockville, MA, USA), 1% amino acid, and 2 mM l-glutamine (both from Sigma-Aldrich Chemie GmbH, Steinheim, Germany). E0771 cell line^31^ was generously provided by Dr Andreas Möller, School of Medicine, University of Queensland, and the Tumour Microenvironment Laboratory, QIMR Berghofer Medical Research Institute, Herston, Queensland, Australia. These cells were cultured in DMEM (Gibco, Scotland, UK) with 10% FBS (GE Healthcare, Chicago, IL, USA) and supplemented with 2 mM l-glutamine (Sigma-Aldrich Chemie GmbH, Steinheim, Germany). All culture media were supplemented with antibiotics (100 µg/mL streptomycin and 100 U/mL penicillin; Sigma-Aldrich Chemie GmbH, Steinheim, Germany and Polfa Tarchomin S.A., Warsaw, Poland, respectively). These cells were maintained in a humidified atmosphere with 5% CO_2_ at 37 °C.

### Cell lysate preparation

Cell lysates were collected using the same procedure from all cell lines (4T1, 67NR, and E0771). To achieve this, cells were initially seeded at the density of 0.2 × 10^6^ in 10 cm culture dishes (Sarstedt, Nümbrecht, Germany). Following a 2-h incubation period, calcitriol (100 nM), vehicle (0.1% ethanol, which served as the solvent for calcitriol), or culture medium was added and maintained for 72 h. Subsequently, cell lysates were harvested using either TRI Reagent solution (Molecular Research Center, Cincinnati, OH, USA) or RIPA lysis buffer containing protease and phosphatase inhibitors (all Sigma-Aldrich Chemie GmbH Steinheim, Germany). These lysates were then utilized for the characterization of these cells by qPCR and western blot analyses, respectively.

### Animal experiments

To investigate the influence of different VD_3_ (cholecalciferol) diets in combination with calcitriol administration on the roles of TAMs and BMDMs in cancer progression, we conducted experiments using female BALB/c (4T1, 67NR) or C57BL/6 (E0771) mice (Charles River Laboratories, Sulzfeld, Germany), following previously established protocols^26^. Briefly, the mice were subjected to the following dietary conditions: normal (control; 1000 IU/kg), deficient (100 IU/kg), and elevated (5000 IU/kg) VD_3_ diets. After 6 weeks on these diets, we orthotopically injected 4T1, 67NR, or E0771 cells (1 × 10^4^, 2 × 10^5^, and 5 × 10^4^, respectively) into mammary fat pad on day 0. The specified diets were continued throughout the experiment (day 28 for 4T1 and 67NR or 23 for E0771 tumor-bearing mice). For mice on the 1000 IU/kg and 100 IU/kg diets, additional oral calcitriol gavage was initiated 7 days after cancer cell inoculation (1 µg/kg; three times a week). The same treatment regimen was applied to healthy BALB/c and C57BL/6 mice, serving as the control groups to contextualize the results observed in tumor-bearing mice. Mice were euthanized by cervical dislocation on day 23 (C57BL/6) or 28 (BALB/c) of cancer cell inoculation under isoflurane (inhalation) anesthesia and buprenorphine analgesia (0.1 mg/kg subcutaneously)^26^. All experiments were conducted in compliance with the EU Directive 2010/63/EU regarding the protection of animals used for scientific purposes and received approval from the first Local Committee for Experiments with the Use of Laboratory Animals, Wroclaw, Poland (permission number: 66/2018). It is worth noting that all tissues used in this study were derived from the animals previously described in our earlier publications and are reported in accordance with ARRIVE guidelines^26,32^.

### Isolation of TAMs

After 1 h of tumor digestion using DNAze I (Roche, Basel, Switzerland) and collagenase IV (Collagenase from *Clostridium histolyticum*; Sigma-Aldrich, Saint-Louis, MO, USA)—both 1 mg/mL) proceeded by mechanical tissue disintegration, the resultant cell suspension was filtered through a 100 µm cell strainer (Easystrainer, Greiner Bio-One, Kremsmünster, Austria). Subsequently, the cells were blocked using an anti-CD16/CD32 blocking antibody (BD Biosciences, San Jose, CA, USA). The cells were then incubated with anti-F4/80 MicroBeads (Miltenyi Biotec Inc., Auburn, CA, USA) and separated using magnetic columns (MACS® Cell Separation Columns—Miltenyi Biotec Inc., Auburn, CA, USA) following the manufacturer’s guidelines. The isolated cells were subsequently subjected to analysis for surface marker expression through flow cytometry or underwent short-term culture (72 h) to purify the material from debris. For the analysis of gene expression via qPCR, lipopolysaccharide (LPS; Sigma-Aldrich, Saint-Louis, MO, USA, 100 ng/mL) was added at a concentration of 100 ng/mL 24 h before lysis in TRI Reagent solution (Molecular Research Center, OH, USA). The cell culture supernatants were utilized for ELISA analysis.

### Isolation of BMDMs and differentiation to macrophages in the presence of tumor cell culture supernatants

**Figure 1:**
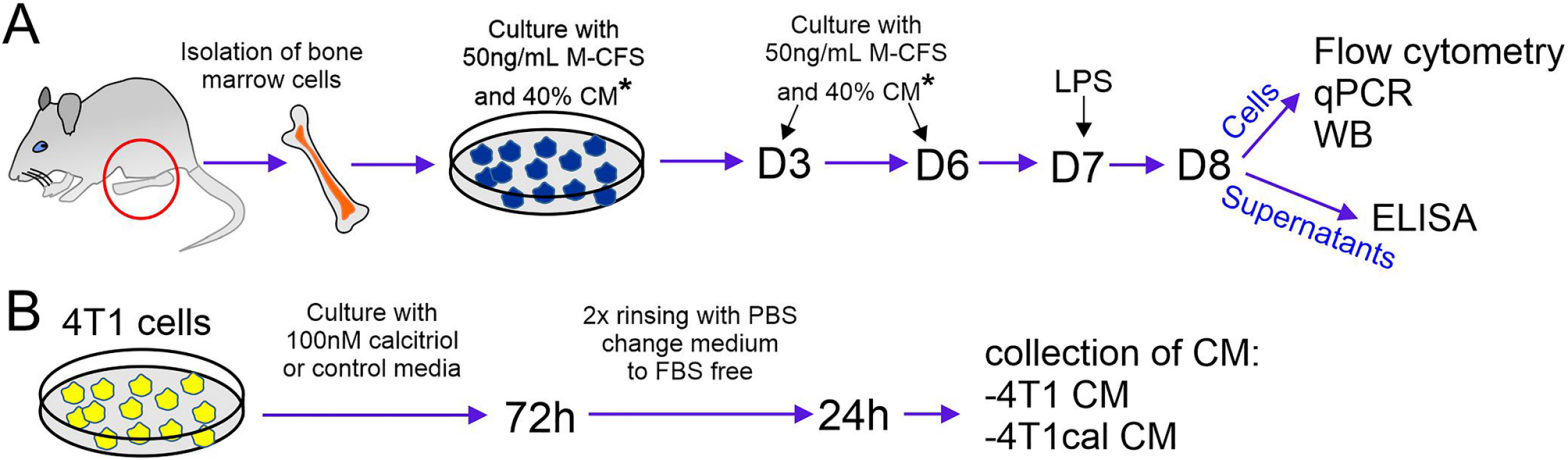
The scheme of bone marrow-derived macrophage (BMDM) differentiation. (**A**) Procedure for the isolation and differentiation of BMDMs. Tumor-bearing or healthy BALB/c mice were fed diets containing various VD_3_ contents (normal: 1000 IU/kg, elevated: 5000 IU/kg, and deficient: 100 IU/kg) for 6 weeks. Statistically significant differences in 25(OH)D_3_ levels were obtained between groups before tumors were inoculated in some mice. Subsequently, calcitriol gavage (1 µg/kg) was started thrice a week for 3 weeks in mice fed with normal and deficient diets, starting from day 7 after cancer cell inoculation. All diets were continued until the day of euthanasia (in total for 10 weeks)^26^. (**B**) Generation of conditioned media from 4T1 cells treated or not with 100 nM calcitriol for 72 h in vitro. *CM from untreated (4T1 CM) and calcitriol-treated (4T1cal CM) 4T1 cells or normal culture medium were used solely during the differentiation of BMDMs from healthy mice.

After euthanizing BALB/c mice bearing 4T1 cancer cells, the femurs and tibias of both legs were placed in cold phosphate-buffered saline (PBS; Institute of Immunology and Experimental Therapy (IIET), Wroclaw, Poland) containing antibiotics. Care was taken not to disrupt the knee and hip joints to maintain bone marrow sterility. The epiphyses of the bones were aseptically removed, and each bone was flushed on both sides with a total volume of 10 mL with cold PBS (IIET, Wroclaw, Poland). The bone marrow cell suspension was centrifuged (432×*g*, 7 min, 4 °C) and subsequently frozen in a solution of FBS and 10% DMSO (both from Sigma-Aldrich, Saint Louis, MO, USA) for further analysis. On thawing, cell quantity and viability were determined using trypan blue solution (Sigma-Aldrich, Saint-Louis, MO, USA). To differentiate BMDMs, cells were counted and seeded into 6-well plates (Greiner Bio-One, Kremsmünster, Austria) at a density of 2 × 10^6^ cells per well. The culture medium (DMEM Gibco (Thermo Fisher Scientific, Waltham, MA, USA) supplemented with 10% FBS (Sigma-Aldrich, Saint Louis, MO, USA), 4 nM l-glutamine (Merck, Darmstadt, Niemcy), 1% MEM nonessential amino-acid solution (Sigma-Aldrich, Saint-Louis, MO, USA), and antibiotics (100 µg/mL streptomycin and 100 U/mL penicillin; Sigma-Aldrich Chemie GmbH, Steinheim, Germany and Polfa Tarchomin S.A., Warsaw, Poland, respectively) was further enriched with 50 ng/mL of mouse recombinant M-CSF (BioLegend, San Diego, CA, USA) on day 1. BMDMs were cultured at 37 °C in a humidified atmosphere with 5% CO_2_ for a total of 8 days. On days 3 and 6, half of the medium volume was replaced with fresh medium, along with an additional 50 ng/mL M-CSF. 24 h before sample collection on day 8, 100 ng/mL of LPS was added. Subsequently, BMDMs were washed twice with PBS (IIET, Wroclaw, Poland) and harvested in TRI Reagent solution (Molecular Research Center, OH, USA) for further qPCR analysis or RIPA buffer for western blot analysis. The supernatant was used for ELISA (Fig. 1A).

Bone marrow from healthy BALB/c mice was isolated as described above. Treatment with 4T1 conditioned medium (CM) or CM from 4T1 cells stimulated with calcitriol (4T1cal CM) was carried out throughout the entire 7-day differentiation period. Specifically, 40% of 4T1 CM was added on days 1, 3, and 6 when the medium was refreshed. The procedure for generating CM is described below. On day 7, 100 ng/mL of LPS (Sigma-Aldrich, Saint-Louis, MO, USA) was added after washing with PBS for 2 times (IIET, Wroclaw, Poland). After 24 h (day 8), supernatants and cells were collected for ELISA, flow cytometry, qPCR, and western blot analysis, respectively (Fig. 1A).

CM from 4T1 cell culture was generated to investigate how factors secreted by calcitriol-stimulated 4T1 cells could influence the phenotype of BMDMs and their cytokine secretion profile. To achieve this, cells were seeded at a density of 0.2 × 10^6^ in 10 cm culture dish (Sarstedt, Nümbrecht, Germany). After 2 h, calcitriol (100 nM) or culture medium was added for 72 h. Next, 4T1 cells were washed twice with PBS (IIET, Wroclaw, Poland), and a fresh medium without FBS was added for media conditioning. After 24 h of incubation, CM from untreated (4T1 CM) and calcitriol-treated (4T1cal CM) cells were collected, centrifuged (10,000×*g*, 10 min, 4 °C), and frozen for further analysis (− 80 °C) (Fig. 1B).

### Phenotype analysis by flow cytometry

To determine the phenotypes of TAMs and BMDMs, 5 × 10^4^ cells were centrifuged (192×*g*, 5 min at 4 °C) and then incubated with a mixture of antibodies (rat anti-mouse antibodies F4/80-BV-421; CD11b-APC; and CD204-BV650 (BD Biosciences, San Jose, CA, USA), major histocompatibility complex class II (MHCII)-PerCP/Cy5.5, epithelial cell adhesion molecule (EpCAM)-FITC; CD44-BV510 (BioLegend, San Diego, CA, USA), CD163-PE (ThermoFisher Scientific, Waltham, MA, USA), and hamster anti-mouse antibodies CD80-PE/Cy7 (BioLegend, San Diego, CA, USA)) for 30 min at 4 °C in the dark. This incubation with antibodies followed TruStain FcX (BioLegend, San Diego, CA, USA) treatment. Afterward, the samples were washed with PBS (IIET, Wroclaw, Poland) and resuspended in 200 µL of 2% FBS (Sigma-Aldrich, Saint Louis, MO, USA) in PBS (IIET, Wroclaw, Poland). Flow cytometry analysis was performed using an LSR Fortessa cytometer with FACSDiva v.8.0.1 software (BD Biosciences, San Jose, CA, USA).

### qPCR analysis

#### Screenings with the use of customized gene arrays

The total RNA was isolated directly from the samples preserved in TRI Reagent (Molecular Research Center Inc, Cincinnati, OH, USA) using the Direct-Zol RNA Kit (Zymo Research, Irvine, CA, USA) following the manufacturer’s protocol. The quantity and purity of isolated RNAs were measured at 260 nm using NanoDrop 2000 (Thermo Fisher Scientific, Waltham, MA, USA). cDNA was synthesized using SuperScript™ IV VILO™ Master Mix (Thermo Fisher Scientific, Waltham, MA, USA). Expression profile of 63 genes (the full list of targets in supplementary data attached: Suppl. Table S1) related to VD_3_ metabolism or TAMs metastatic properties were analyzed using custom 384-well TaqMan™ Array cards (microfluidic cards), following the manufacturer’s protocol (Thermo Fisher Scientific, Waltham, MA, USA). For each fill reservoir, 75 ng of pooled cDNA (3 mice from each group) and TaqMan™ Gene Expression Master Mix (Thermo Fisher Scientific, Waltham, MA, USA) were used. Data calculation for array cards included the use of multiple endogenous controls: 18S-Hs99999901_s1 (technical control of the manufacturer), B2m-Mm00437762_m1, Actb-Mm01205647_g1, and Gapdh-Mm99999915_g1. Experiments conducted on array cards, as well as real-time quantitative PCR of separate samples from each treatment group, were performed using ViiA™7 device (Thermo Fisher Scientific, Waltham, MA, USA). All qPCR analyses were conducted using ExpressionSuite Software v1.3 (Thermo Fisher Scientific, Waltham, MA, USA) and the ΔΔCt analysis method. The 1000 IU group was chosen as the calibrator, and the results were presented as RQ (fold-change).

### qPCR analysis of selected genes

The list of genes selected for further analysis based on screening tests is provided in Table 1. RNA isolation and reverse transcription were carried out as described earlier. For the qPCR analysis of the selected genes, BMDMs from 6 mice in each group bearing 4T1 cancer cells were used. Gene expression assays were conducted using 25 ng cDNA/reaction (10 µL final volume), 2 × TaqMan™ Gene Expression Master Mix, and TaqMan™ probes (Thermo Fisher Scientific, Waltham, MA, USA). Endogenous controls used are listed in Table 1.

**Table 1:** TaqMan™ gene expression assays are utilized for RT-PCR.

Gene	Assay number
*Gapdh*	Mm99999915_g1
*Hprt*	Mm00446968_m1
*B2m*	Mm00437762_m1
*Cyp27b1*	Mm01165918_g1
*Cyp24a1*	Mm00487244_m1
*Vdr*	Mm00437297_m1
*Il6*	Mm00446190_m1
*Il23*	Mm00518984_m1
*Irf4*	Mm00516431_m1
*Fn1*	Mm01256744_m1
*Mrc1*	Mm01329362_m1
*Tgfb1*	Mm01178820_m1
*Tnfa*	Mm00443258_m1
*Arg1*	Mm00475988_m1
*Infg*	Mm01168134_m1
*Spp1*	Mm00436767_m1

Endogenous controls: Glyceraldehyde-3-phosphate dehydrogenase (*Gapdh*); hypoxanthine phosphoribosyltransferase 1 (*Hprt*); and beta-2-microglobulin (*B2m*). Target genes: cytochrome P450 family 27 subfamily B member 1 (*Cyp27b1*); cytochrome P450, family 24, subfamily a, polypeptide 1 (*Cyp24a1*); vitamin D receptor (*Vdr*); interleukin 6 (*Il6*), interleukin 23 (*Il23*), interferon regulatory factor 4 (*Irf4*); fibronectin 1 (*Fn1*); mannose receptor C-type 1 (*Mrc1*); transforming growth factor-beta 1 (*Tgfb1*); tumor necrosis factor alpha (*Tnfa*); arginase 1 (*Arg1*); interferon gamma (*Infg*); and osteopontin (*Spp1*).

### ELISA

TAMs and BMDMs supernatants, CM from 4T1 cells, and tumor tissue homogenates were assessed for protein secretion and lysate content. ELISA kits were used for this purpose, specifically IL-6 (Wuhan EIAab Science Co, Ltd, Wuhan, China), PGE_2_, and IL-23 (Bio-techne® R&D System, Minneapolis, MN, USA). The assays were conducted following the manufacturer’s instructions. Results were obtained using a plate reader (BioTek Instruments, Winooski, VT, USA), and the best standard curve was selected using CurveExpert ver. 1.4 software.

### Western blot

Cell culture lysates and tissue homogenates were prepared in RIPA buffer supplemented with a protease inhibitor cocktail (both from Sigma-Aldrich, Steinheim, Germany). Subsequently, samples were centrifuged for 10 min at 4 °C and 10,000×*g*. Total protein concentration analysis was performed using the modified Lowry method (Bio-Rad, Warsaw, Poland) following the manufacturer’s protocol. Samples containing 50 μg of protein were subjected to western blot analysis, as described previously^26^. Wash buffer contained 0.1% of Tween-20 (Sigma-Aldrich, Steinheim, Germany) in TBS (TBS-T; IIET, Wroclaw, Poland)). For membrane blocking, 5% nonfat dry milk in TBS-T was used. Membranes were incubated overnight at 4 °C with primary antibodies at concentrations recommended by the manufacturers. For murine samples, the primary antibodies used were as follows: rabbit anti-EpCAM (21050-1-AP); rabbit anti-osteopontin (OPN; 22952-1-AP); rabbit anti-transforming growth factor (TGF-β; 21898-1-AP); rabbit anti-zinc finger E-box-binding homeobox 1 (Zeb1; 21544-1-AP); rabbit anti-JUN (AP-1, 24909-1-AP); rabbit anti-interferon regulatory factor 4 (IRF4; 11247-1-AP); rabbit anti-E-cadherin (20874-1-AP) (all above from Protein-tech®, Wuhan, China); rabbit anti-VDR monoclonal antibody (D2K6W, CellSignaling Technology, Danvers, MA, USA); rabbit anti-CYP24A1 polyclonal antibody (H087, #sc-66851; Santa Cruz Biotechnology, Dallas, TX, USA); rabbit anti-CYP27B1 monoclonal antibody (ab206655; Abcam, Cambridge, UK); rabbit anti-matrix metalloproteinase 3 (MMP-3; NBP2-75931), rabbit anti-exosome endoribonuclease and 3ʹ-5ʹ exoribonuclease polyclonal antibody (DIS3; NBP2-81804), rabbit anti-parathyroid hormone like hormone (PTHLH; NBP3-03168) (all above from Novus, Bio-techne® R&D System, Minneapolis, MN, USA); mouse anti-cyclooxygenase 2 (COX-2; BD 610203; BD Biosciences, San Jose, CA, USA); and rabbit anti-protein disulfide-isomerase A3 (PDIA3; ERP57; 2881S, Cell Signaling Technology, Danvers, MA, USA). For human samples, the antibodies used were as follows: anti-PTGS2 (COX-2; 12375–1-Ap, Proteintech Rosemont, IL, USA), anti-CYP24A1 (ab203308, Abcam, Cambridge, UK), anti-VDR (bs2987, Bioss Antibodies, Woburn, MA, USA), and anti-CYP27B1—the same as mentioned above. Secondary antibodies, used at concentrations recommended by the manufacturer, were goat anti-mouse IgG-HRP antibody (P0447; Dako, Carpinteria, CA, USA) and mouse anti-rabbit IgG-HRP (sc-2357; Santa Cruz Biotechnology, Dallas, TX, USA). Detection reactions were carried out using Clarity Western ECL Substrate (Bio-Rad, Hercules, CA, USA), and chemiluminescence visualization was performed on ChemiDoc Imaging System (Bio-Rad, Hercules, CA, USA). Mouse anti-β-actin-HRP antibody (sc-47778, Santa Cruz Biotechnology, Dallas, TX, USA) was used for target protein normalization, and densitometry analysis was performed using ImageJ 1.48v software.

### Bone marrow metastasis

After thawing the bone marrow samples collected from 4T1 tumor-bearing mice, the cells were counted, and 10 million cells from each mouse in the group were plated on a 10 cm petri dish (Sarstedt, Nümbrecht, Germany). After 24 h, 10 µM of 6-thioguanine (4T1 cells are resistant to this compound) (Sigma-Aldrich, Saint-Louis, MO, USA) was added to the culture medium. The cells were left for 14 days and cultured at 37 °C in a humidified atmosphere with 5% CO_2_. After the incubation time, metastatic 4T1 colonies were stained with a 20% crystal violet solution (Sigma-Aldrich, Saint Louis, MO, USA) in 80% methanol (Avantor, Gliwice, Poland) and counted.

### Patients

Breast cancer patients diagnosed between October 2019 and December 2020 at Lower Silesian Oncology, Pulmonology, and Hematology Center (Wroclaw, Poland) were enrolled in the study. These patients had not undergone any prior anticancer treatment. Blood samples were collected from patients 1 day before surgery, and tumor tissues were obtained during surgery. The patients were categorized into groups based on their menopausal status (on the basis of plasma FSH level: FSH > 25.8 mIU/mL postmenopausal and FSH < 25.8 mIU/mL premenopausal), VD_3_ status (normal: 25(OH)D_3_ ≥ 30 ng/mL and deficient: 25(OH)D_3_ < 30 ng/mL plasma level), and expression levels of selected tumor proteins. The median value of densitometric analysis (protein tested/β-actin) was used as a threshold to divide patients into groups with high and low levels of VDR, CYP24A1, or CYP27B1 (Table 2). The study was conducted in accordance with the ethical standards of both the national and institutional research committees, as well as with the most recent version of the 1964 Helsinki Declaration. The study protocol received approval from the local Institutional Review Board, Wroclaw, Poland (permission no 603/2018), and informed consent was obtained from every patient.

**Table 2:** Overview of the characteristics of BC patients.

Parameter	Patient characteristics		Statistics
	Premenopausal	Postmenopausal	
No	23	37	–
Age	47 (32–59)	66 (48–83)	*P* < 0.0001
25(OH)D_3_ [ng/mL]	23.6 (11.5–50.4)	23.4 (7.14–60.1)	ns
VDR	0.15 (0.01–2.61)	0.17 (0.01–4.33)	ns
CYP24A1	0.514 (0.22–2.04)	0.609 (0.16–2.74)	ns
CYP27B1	0.41 (0.02–5.17)	0.49 (0.02–2.79)	ns
COX-2	0.44 (0.003–4.53)	0.33 (0.02–3.85)	ns

	VD_3_ deficient	VD_3_ normal	
No	42	18	–
Age	57.5 (32–83)	57.5 (46.5–83)	ns
25(OH)D_3_ [ng/mL]	20.7 (7.14–29.2)	36.5 (31.1–60.1)	*P* < 0.0001

	VDR low	VDR high	
No	30	30	–
Age	56 (35–73)	61 (32–83)	ns
25(OH)D_3_ [ng/mL]	25.0 (9.2–60.1)	22.4 (7.14–50.4)	ns
VDR	0.05 (0.01–0.15)	0.34 (0.16–4.33)	*P* < 0.0001

	CYP24A1 low	CYP24A1 high	
No	30	30	–
Age	58 (36–83)	57 (32–80)	ns
25(OH)D_3_ [ng/mL]	26.9 (10.0–45.0)	22.3 (7.14–60.1)	ns
CYP24A1	0.36 (0.16–0.54)	1.14 (0.61–2.74)	*P* < 0.0001

	CYP27B1 low	CYP27B1 high	
No	30	30	*–*
Age	58.5 (32–83)	56.5 (35–83)	ns
25(OH)D_3_ [ng/mL]	23.5 (7.14–60.1)	24.05 (9.2–41.5)	ns
CYP27B1	0.14 (0.02–0.48)	0.92 (0.49–5.17)	*P* < 0.0001

No of all patients = 60; age of all patients = 57.5 (32–83) years; 25(OH)D_3_ level of all patients = 23.55 (7.14–60.10) ng/mL; VDR (VDR/β-actin ratio) of all patients = 0.16 (0.01–4.33); CYP24A1 (CYP24A1/β-actin ratio) of all patients = 0.58 (0.16–2.74); CYP27B1 (CYP27B1/β-actin ratio) of all patients = 0.49 (0.02–5.17); COX-2 (COX-2/β-actin ratio) of all patients = 0.35 (0.003–4.53). Median values with a min–max range are presented. Statistical analysis: Mann–Whitney test.

### Statistical analysis

Statistical analysis was performed using GraphPad Prism 7.01 (GraphPad Software Inc., San Diego, CA, USA). The assumptions for the analysis of variance (ANOVA) were evaluated using Shapiro–Wilk’s normality test and D’Agostiono-Pearson tests. *P*-value ≤ 0.05 was considered significant, and the specific tests used for each data analysis are specified in the figure legends.

### Ethics approval and consent to participate

All animal experiments were performed in accordance with the EU Directive 2010/63/EU on the protection of animals used for scientific purposes and were approved by the first Local Committee for Experiments with the Use of Laboratory Animals, Wroclaw, Poland (permission no. 66/2018). Patient tissue studies were carried out in line with the ethical standards provided by the national and institutional research committee and with the latest version of the 1964 Helsinki Declaration. The study protocol obtained approval from the local Institutional Review Board, Wroclaw, Poland (permission no. 603/2018). All participants provided written informed consent.

## Results

### Characterization of 4T1, 67NR, and E0771 cells for the expression of VDR, VD_3_ metabolizing enzymes, and tumor tissues for molecules distinctive for metastatic cells

**Figure 2:**
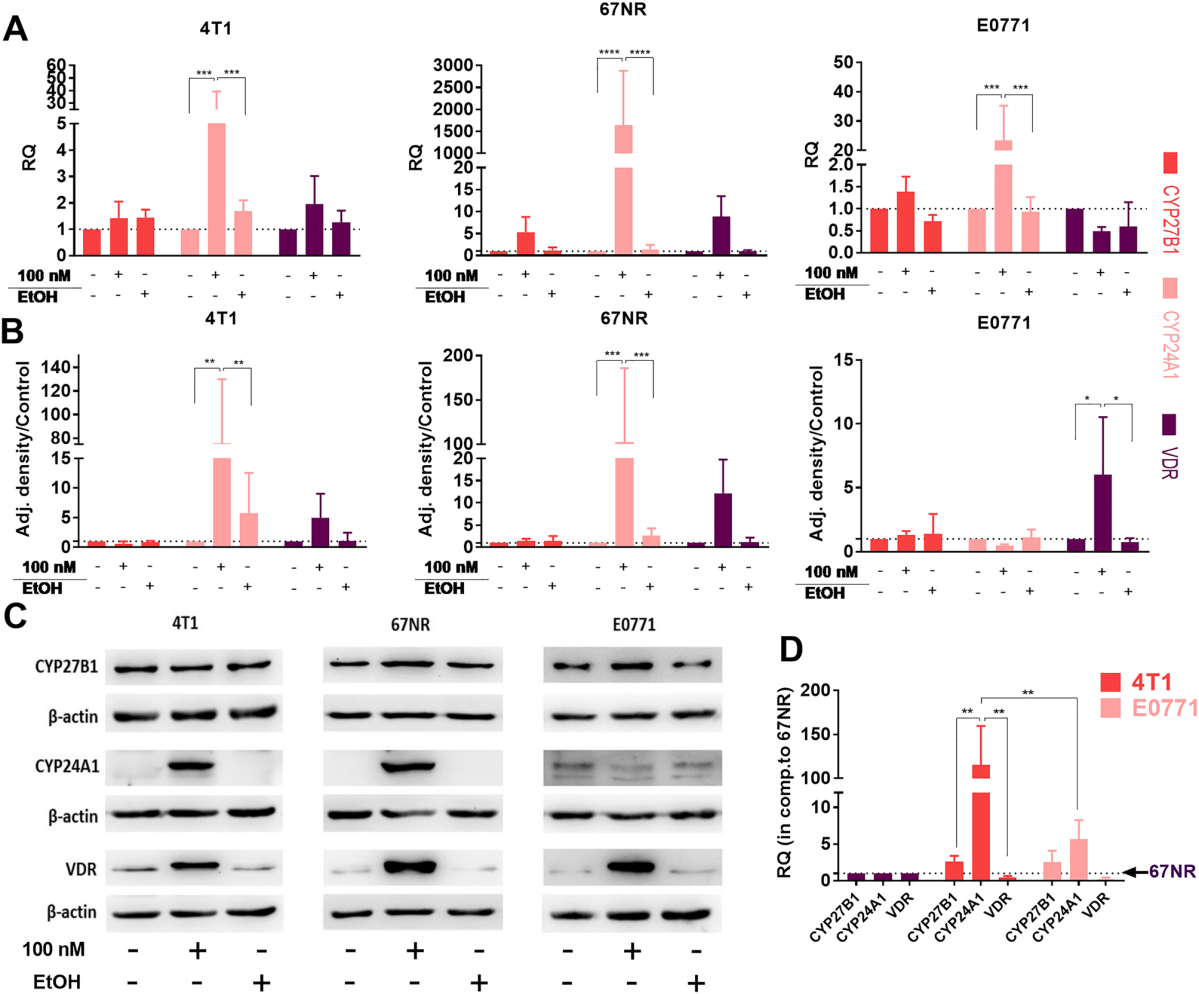
Characterization of 4T1, 67NR, and E0771 cell lines used in animal studies in vitro after 72 h of treatment with 100 nM of calcitriol. (**A**) Real-time PCR analysis of *Cyp24a1*, *Cyp27b1*, and *Vdr* gene expressions. Untreated cells of each model served as a calibrator, and calculations were performed using multiple endogenous controls (*Hprt*, *B2m*, and *Gapdh*); *N* = 3. Statistical analysis: Tukey’s multiple comparisons test. (**B**) Densitometry analysis of target protein level; β-actin was used as the loading control. *N* = 3. (**C**) Representative blots for 4T1, 67NR, and E0771 (from left, respectively). (**D**) Real-time PCR analysis of the selected genes in untreated 4T1 and E0771 cell lines compared with untreated 67NR cells. Statistical analysis: Sidak’s multiple comparisons test. *P < 0.05; **P < 0.01; ***P < 0.001; ****P < 0.0001.

In vitro characterization of the cell lines used in animal studies was performed due to variations in their antiproliferative response to calcitriol. Our previous research demonstrated that 4T1^25^ and E0771^26^ cells do not exhibit sensitivity to proliferation inhibition by calcitriol, whereas the proliferation of 67NR cells is markedly inhibited^25^. In this study, we compared the impact of 100 nM calcitriol treatment (72 h) on the expression of VD_3_ metabolizing enzymes and VDR (Fig. 2). We observed the induction of CYP24A1 at both the mRNA and protein levels in both 4T1 and 67NR cells, whereas in E0771 cells, only mRNA expression increased (Fig. 2A–C). When we compared the baseline mRNA level of these molecules in control (untreated) cells, we noticed the highest *Cyp24a1* expression in 4T1 cells compared with 67NR and E0771. Furthermore, metastatic 4T1 and E0771 cell lines exhibited lower *Vdr* gene expression compared with the non-metastatic 67NR cell line (Fig. 2D).

Tumors collected from mice subjected to varying VD_3_ diets and treated with calcitriol were subjected to western blot analysis to assess the expression of molecules that have previously been identified as distinctive for metastatic 4T1 or E0771 tumors compared with non-metastatic 67NR^31,33^. Notably, 4T1 tumor growth remained unaffected in treatment groups, whereas in 67NR-bearing mice, transient (days 23–25) tumor growth inhibition was observed in mice from the 1000 IU + cal group compared with control. In E0771 tumor-bearing mice, only a tendency toward tumor growth inhibition (not statistically significant) was observed in 1000 IU + cal and 5000 IU groups^26^. PDIA3 expression was reduced in 4T1 tumors from mice fed VD_3_ 5000 IU and 100 IU diets (Suppl. Fig. S1A and B). In these same tumors, the highest expression of DIS3 was observed in mice treated with calcitriol (1000 IU + cal and 100 IU + cal), whereas the highest expression of MMP3 in mice from 5000 IU VD_3_ group (Suppl. Fig. S1C). In 67NR tumors, the lowest expression of MMP3 and DIS3 was observed in the 100 IU + cal group (Suppl. Fig. S1C). The expression of other tested proteins (β-catenin, E-cadherin, and PTHLH) did not exhibit significant changes during treatment, or only the tendencies toward changes were observed (Suppl. Fig. S1A–C).

### Phenotype analysis of TAMs from mice bearing 4T1, 67NR, and E0771 tumors fed diets with varying VD_3_ content and treated with calcitriol

**Figure 3:**
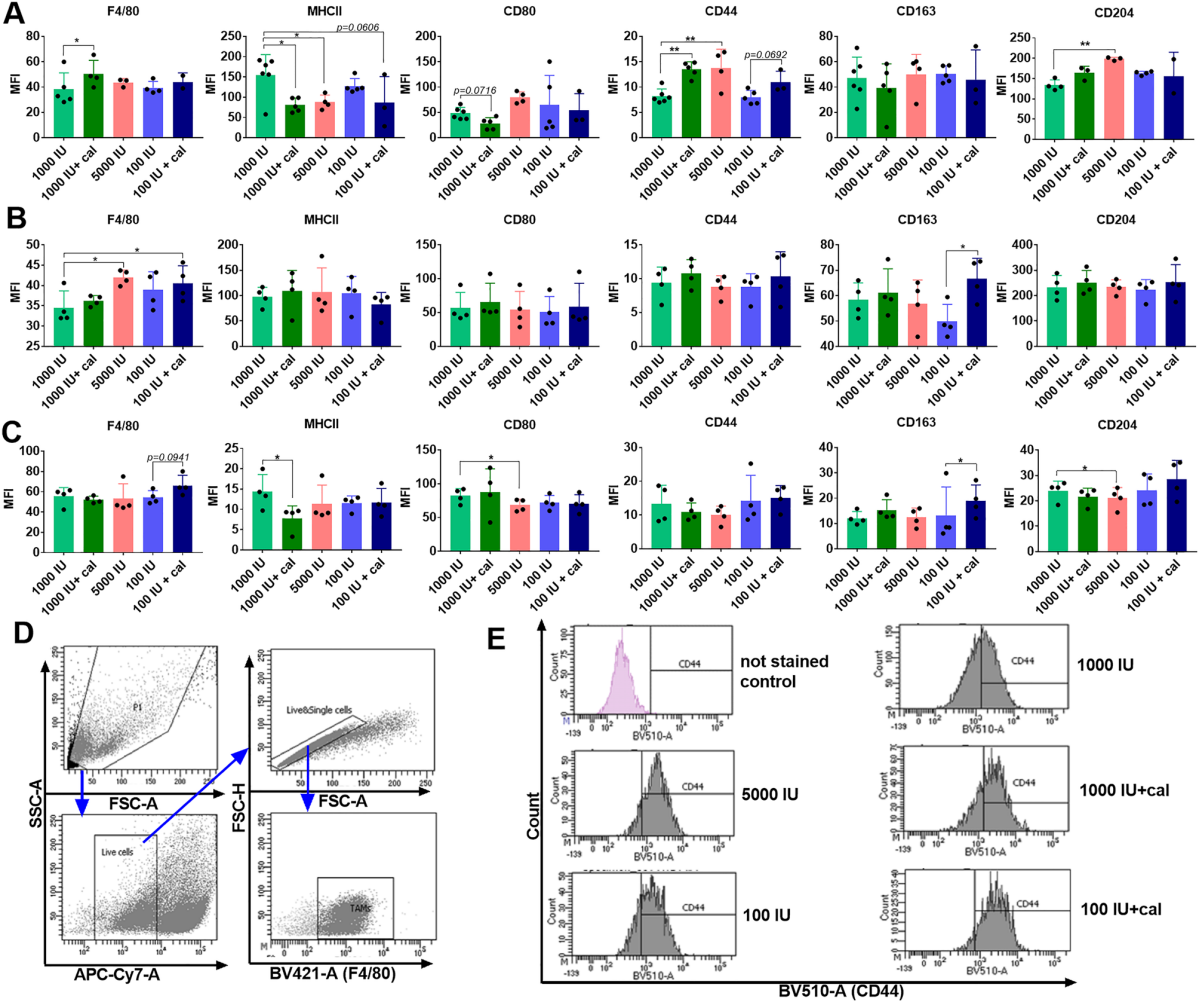
Phenotype analysis of TAMs from mice bearing 4T1, 67NR, and E0771 tumors fed diets with varying VD_3_ contents and treated with calcitriol. Flow cytometry analysis was performed using freshly isolated TAMs from (**A**) 4T1, (**B**) 67NR, and (**C**) E0771 tumors. Expression of the following macrophage markers was analyzed: F4/80, MHCII, CD44, CD80, CD163, and CD204. Data are presented as median fluorescence intensity (MFI), with mean values and SD, as well as data for individual mice. (**D**) Gating strategy for TAMs—live, F4/80-positive cells. (**E**) Representative histograms showing CD44 expression on TAMs isolated from 4T1 tumors and stained with an anti-CD44 antibody (F4/80^+^CD44^+^ cells). *N* = 3–6 independent TAM separations. Statistical analysis: Dunn’s multiple comparisons test; *P < 0.05, **P < 0.01.

To characterize the TME features, we conducted a phenotyping analysis of TAMs (F4/80^+^ macrophages) using flow cytometry. For this purpose, we selected MHCII and CD80 as characteristic markers for M1 macrophages, and CD44, CD163, and CD204 for M2 macrophages^34–36^. Major macrophage marker F4/80 increased in mice bearing 4T1 tumors fed a normal VD_3_ diet and treated with calcitriol (group 1000 IU + cal) (Fig. 3A). F4/80 was also increased in 67NR-bearing mice fed 5000 IU and in mice fed 100 IU VD_3_ diet and treated with calcitriol (100 IU + cal) (Fig. 3B). In E0771-bearing mice, we observed only a tendency to increase F4/80 expression due to calcitriol in mice fed VD_3_-deficient diet (Fig. 3C). MHCII expression on F4/80^+^ cells was lower in mice bearing 4T1 and E0771 tumors from groups 1000 IU + cal (Fig. 3A and C). Also, a VD_3_-elevated diet (5000 IU) led to a decrease in MHCII expression on TAMs from 4T1 tumors, with a similar tendency observed in the 100 IU + cal group (Fig. 3A). The changes in CD44 expression were noticed only in the 4T1 TAMs, where it increased in both the 1000 IU + cal and 5000 IU groups. A similar tendency caused by calcitriol was observed among mice fed a VD_3_ deficiency diet (100 IU + cal) (Fig. 3A and E). CD80 showed a tendency to decrease on 4T1 TAMs from the 1000 IU + cal group, and a significant decrease of this marker was observed on E0771 TAMs from the 5000 IU group (Fig. 3A and C). A significant increase in CD163 caused by calcitriol was observed on 67NR and E0771 TAMs from VD_3_ deficiency groups (Fig. 3B and C). CD204 expression increased on 4T1 TAMs and decreased on E0771 TAMs from mice fed a VD_3_-elevated diet (5000 IU) (Fig. 3A and C). The gating strategy for TAMs is presented as live F4/80-positive cells, and example representative histograms showing CD44 expression on TAMs (F4/80^+^CD44^+^) isolated from 4T1 tumors are presented (Fig. 3D and E). Representative histograms for the remaining staining are presented in Suppl. Fig. S2).

These results indicate that phenotypic markers of TAMs isolated from 4T1 tumors, but not from 67NR and E0771 tumors in groups of mice fed 1000 IU + cal, 5000 IU, and 100 IU + cal diets, exhibit a more pronounced pro-tumor M2 polarization.

BMDMs from 4T1-bearing mice were also phenotyping, showing only decreased CD204 expression in the 5000 IU group (Suppl. Figs. S3 and S4).

### The expression of genes related to macrophage function and VD_3_ action in TAMs and BMDMs derived from 4T1 and 67NR tumor-bearing mice

**Figure 4:**
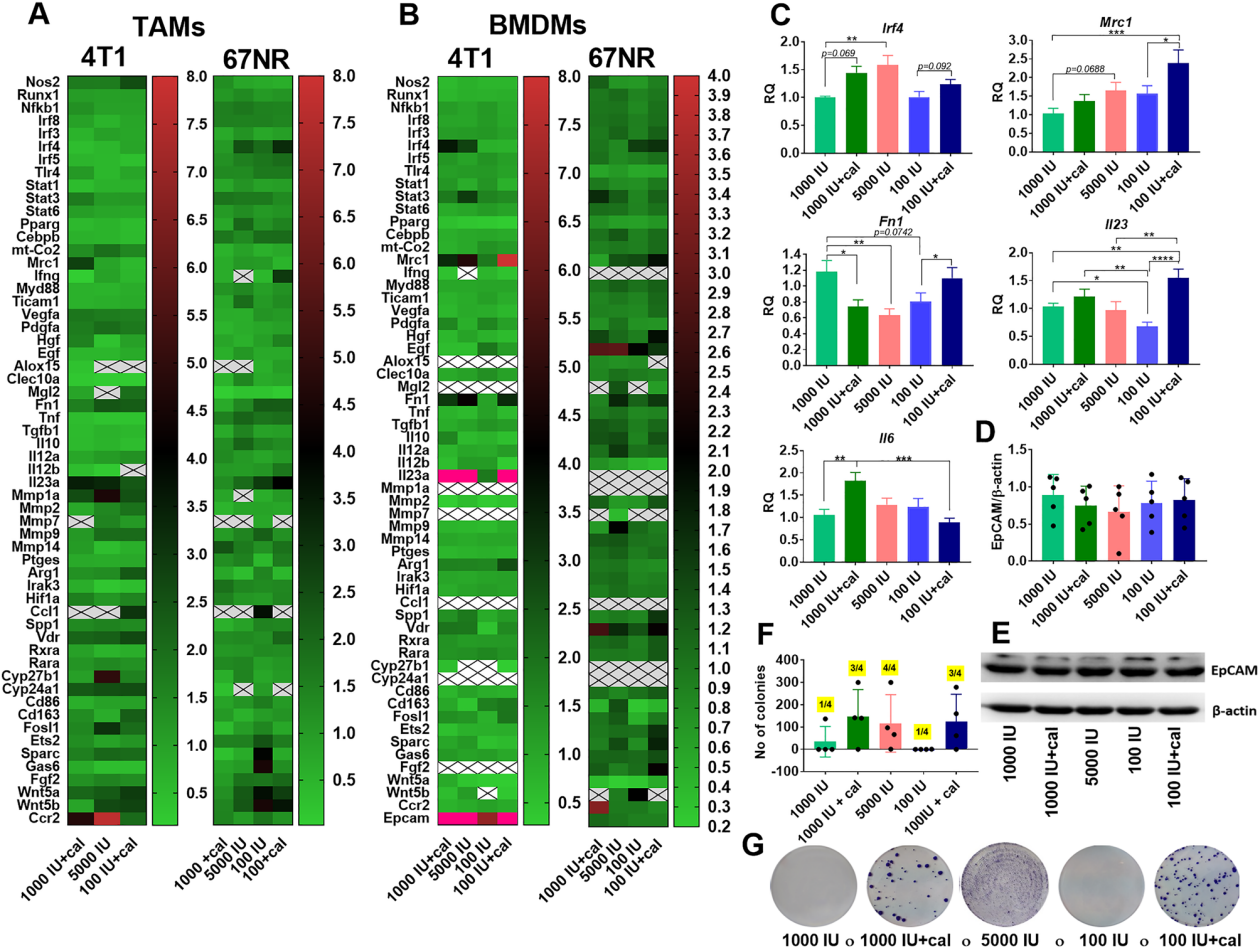
Screening of TAMs and BMDMs from 4T1 and 67NR tumor-bearing mice for gene expression. Heat maps of expression of selected genes related to macrophage function and mechanisms of action of VD_3_ in (**A**) tumor-associated macrophages (TAMs; freshly isolated, plated for additional 3 days, and then treated with LPS, followed by analysis; N = material pooled from 2 to 4 separations, with each separation consisting of TAMs isolated from 3 mice) and (**B**) bone marrow-derived macrophages (BMDMs; isolated bone marrow cells were seeded with the presence of 50 ng/mL of M-CSF for 7 days, then treated with LPS, followed by analysis; *N* = 3 mice). On heat maps: pink indicates values higher than those shown on the scale; crossed-out boxes indicate no detectable expression. (**C**) Real-time PCR analysis of selected genes in 4T1 BMDMs; *N* = 3. It was impossible to isolate the appropriate number of TAMs from 4T1 tumors from mice fed a deficient diet (100 IU). (**A**–**C**) The RQ data are normalized to the 1000 IU group (normal diet—control group). (**D**) Western-blot analysis of epithelial cell adhesion molecule (EpCAM) expression in 4T1 BMDMs; *N* = 5. (**E**) An example membrane showing western blot analysis of EpCAM. (**F**) Number of 4T1 colonies in the bone marrow. Bone marrow cells were seeded in the presence of 10 µM of 6-thioguanine and cultured for 14 days. The 4T1 colonies were stained with crystal violet and counted; *N* = 4. (**G**) An example image of stained 4T1 colonies. Data are shown as the mean with standard error of the mean (SEM). Statistical analysis: Dunn’s multiple comparisons test; *P < 0.05, **P < 0.01, ***P < 0.001, ****P < 0.0001.

To compare the effect of VD_3_ on metastatic and non-metastatic TME cells, PCR arrays were performed on TAMs isolated from mice bearing 4T1 and 67NR tumors as well as on BMDMs differentiated to macrophages in the presence of M-CSF (Fig. 4A and B, Supplementary Tables S1 and S2). Genes were selected based on published data to screen the most important molecules related to macrophage function and the action of VD_3_. The first gene whose expression differed between groups of mice and between tumors is interferon regulatory factor 4 (*Irf4*)*.* It is downregulated in 4T1 TAMs from the 1000 IU + cal and 5000 IU groups but upregulated in 67NR TAMs from 5000 and 100 IU + cal groups. However, in BMDMs isolated from the same 4T1-bearing mice, this gene was upregulated in 1000 IU + cal and 5000 IU groups, and its expression did not change significantly in 67NR BMDMs (Fig. 4A and B). Real-time PCR analysis was additionally performed on BMDMs from 4T1-bearing mice, confirming these observations for *Irf4* (Fig. 4C). The treatment of 4T1 tumor-bearing mice with calcitriol seemed to increase the expression of the mannose receptor C-type 1 (*Mrc1*) gene in TAMs and BMDMs, with the highest expression of this gene observed in TAMs from 1000 IU + cal group and in BMDMs from the 100 IU + cal group (Fig. 4A and B); this observation was confirmed in the qPCR analysis of 4T1 BMDMs (Fig. 4C). Fibronectin-1 (*Fn1*) and *Il23* genes were increased in TAMs and BMDMs from 4T1 mice treated with calcitriol and from the 5000 IU group (Fig. 4A and B), but this observation was confirmed in BMDMs only for the group of mice fed a VD_3_ deficiency diet and treated with calcitriol (100 IU + cal) (Fig. 4C). We also noticed a high expression of C–C chemokine receptor type 2 (*Ccr2*) in 4T1 TAMs from 1000 IU + cal and 5000 IU groups (Fig. 4A). Increased *Il6* expression was noticed in 4T1 BMDMs from 1000 IU + cal group (Fig. 4C). The expressions of *Tgfb1* and *Arg1* were also analyzed by qPCR but did not yield significant results (Suppl. Fig. S5A). In 4T1 BMDMs from all treated groups, the expression of epithelial cell adhesion molecule (*Epcam*) was highly increased (27–98 times for calcitriol-treated or VD_3_-elevated diet groups and 6 times for VD_3_ deficiency group; Table S2). However, at the protein level, we did not observe any differences in EpCAM expression in BMDMs between groups of 4T1 tumor-bearing mice (Fig. 4D and E).

After culturing bone marrow cells in the presence of 6-thioguanine (which selectively eliminates cells other than cancer cells, as 4T1 cells are resistant to 6-thioguanine^37^), we observed the growth of the 4T1 cell colonies. Specifically, we detected 4T1 cell colonies in one mouse from the 1000 IU group (137 colonies) and in one mouse from the 100 IU group (1 colony). In the remaining groups (1000 IU + cal, 5000 IU, and 100 IU + cal), 3 or 4 of the 4 cultures (1 culture = 1 mouse) led to the growth of metastatic 4T1 cells, with colony counts ranging from 142–300; 1–300; 62–279 colonies, respectively (Fig. 4F and G). The binominal test (https://www.graphpad.com/quickcalcs/binomial1/) to estimate the probability of developing bone metastases in the study groups was used. The baseline probability in the control group (incidence rate) is 25%. On this basis, the following *P* values were calculated in individual study groups: 1000 IU + cal *P* = 0.05, 5000 IU *P* = 0.039, 100 IU *P* = 0.68 and 100 IU + ca *P* = 0.05.

### Evaluation of inflammatory cytokines IL-23 and IL-6 secretion by TAMs and BMDMs

**Figure 5:**
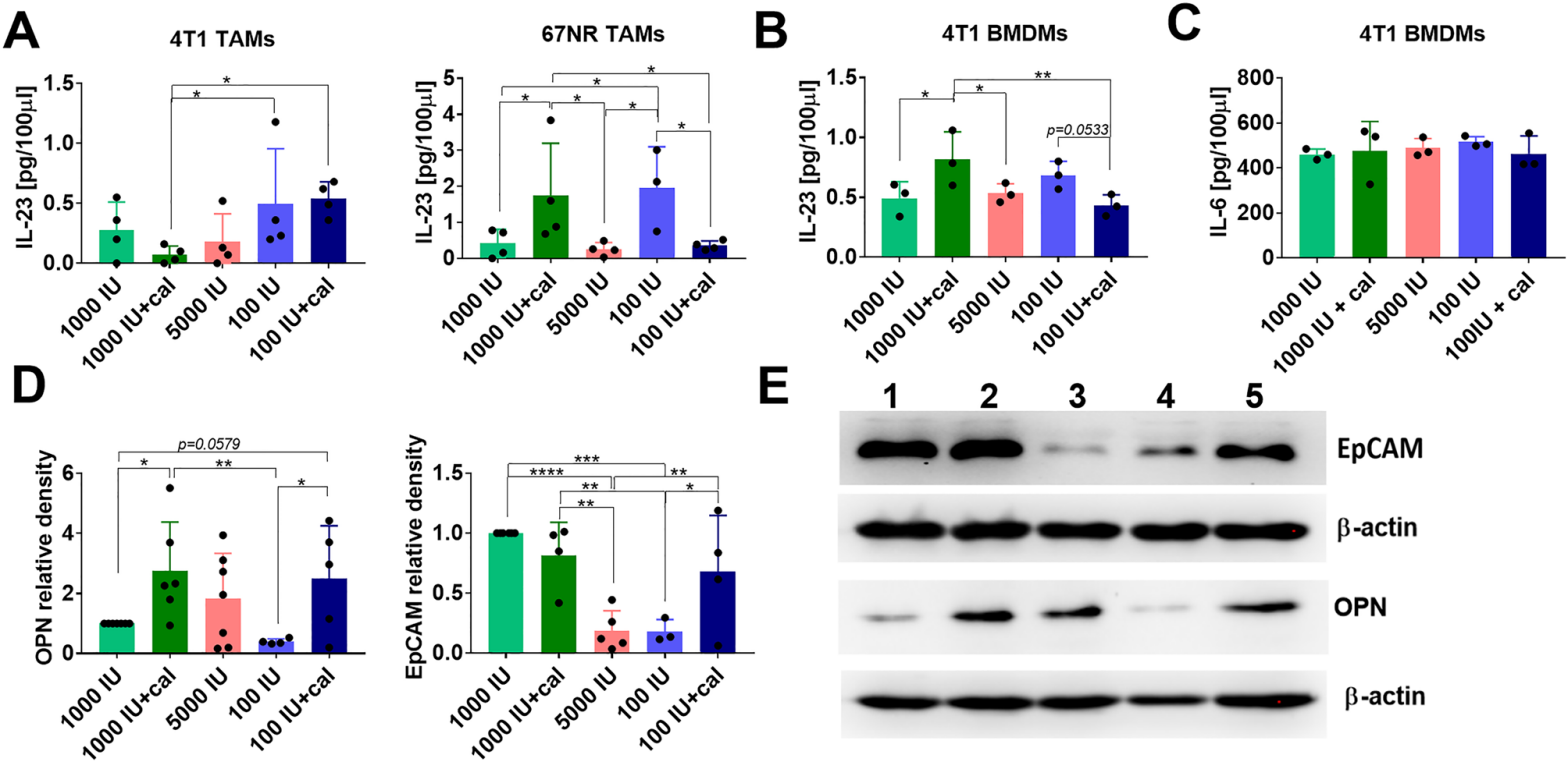
Inflammatory cytokines secreted by TAMs and BMDMs, and tumor tissue expression of osteopontin (OPN) and epithelial cell adhesion molecule (EpCAM). (**A**) The concentration of IL-23 secreted by 4T1 and 67NR TAMs stimulated by 100 ng/mL LPS for 24 h. (**B**) IL-23 and (**C**). IL-6 secretion by 4T1 BMDMs. ELISA assays were conducted to evaluate the secretion of IL-23 (TAMs and BMDMs) and IL-6 for BMDMs only. Supernatants were collected after 24 h of 100 ng/mL LPS stimulation. (**D**) Western blot evaluation of OPN and EpCAM levels in 4T1 tumor tissue homogenates. (**E**) Representative blots of OPN and EpCAM tumor tissue analysis: 1–1000 IU, 2–1000 IU + cal, 3–5000 IU, 4–100 IU, and 5–100 IU + cal. The number of repeats is presented on the graph. Statistical analysis: Sidak’s multiple comparison test; *P < 0.05, **P < 0.01, ***P < 0.001, ****P < 0.0001.

Based on the above results, we selected IL-23 and IL-6 for further evaluation in culture supernatants from TAMs isolated from 4T1 and 67NR tumors. We observed the highest levels of IL-23 in 4T1 TAMs supernatants isolated from both groups of mice fed a VD_3_-deficient diet (100 IU and 100 IU + cal). In contrast, TAMs from 67NR-bearing mice fed a normal VD_3_ diet and treated with calcitriol (1000 IU + cal) showed significantly higher production of IL-23 in culture media compared with TAMs from mice fed a VD_3_-deficient diet and treated with calcitriol (100 IU + cal) (Fig. 5A). The same profile of IL-23 secretion into culture media was observed for 4T1 BMDMs (Fig. 5B). IL-6 levels remained consistently high in 4T1 BMDMs media across all groups of mice (Fig. 5C).

### Expression of selected proteins in 4T1 BMDMs and 4T1 tumor tissue

In 4T1 BMDMs, Western blot analysis was conducted to assess the expressions of CYP27B1, VDR, OPN, TGFβ, PDIA3, MMP3, ZEB1, AP-1, and IRF4 (Suppl. Fig. S5B). We observed a decreased expression of CYP27B1 in the 100 IU + cal group compared with the control. PDIA3 expression was significantly higher in the 1000 IU + cal group compared with 5000 IU, 100 IU, and 100 IU + cal groups. Significantly lower expression of MMP3 was observed in BMDMs from the 1000 IU + cal group, and a similar trend was observed for IRF4 expression (Suppl. Fig. S5B).

In 4T1 tumor tissue lysate, we also analyzed the expressions of OPN and EpCAM, and we found that calcitriol, in mice fed VD_3_ normal (1000 IU + cal) and deficient (100 IU + cal) diets, significantly increased OPN levels. The expression of EpCAM was the lowest in mice fed VD_3_-elevated (5000 IU) and -deficient (100 IU) diets (Fig. 5D and E).

The expressions of AP-1, IRF4, Zeb1, and TGFβ in 4T1 tumor tissue are presented in Supp. Fig. S5C. Only the expression of TGFβ increased significantly in mice fed a VD_3_ 5000 IU diet (Suppl. Fig. S5C).

### Expression of M1 and M2 markers on BMDMs derived from healthy mice being on varied VD_3_ diets and treated with calcitriol cultured ex vivo with the 4T1 culture supernatants

**Figure 6:**
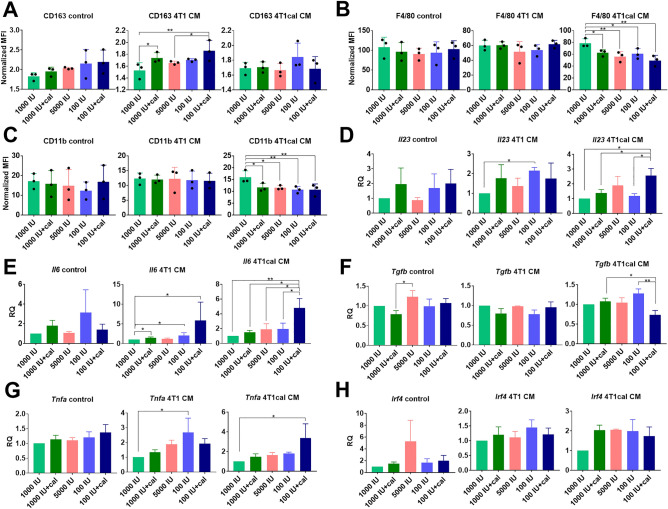
Characterization of BMDMs differentiated ex vivo with 4T1 conditioned medium (CM). BMDMs were derived from healthy mice fed diets containing varying contents of VD_3_ and treated with calcitriol. BMDMs were differentiated in the presence of 50 ng/mL M-CSF and 40% v/v CM-conditioned medium. CM was derived from 100 nM calcitriol-stimulated (4T1cal CM) or not-stimulated 4T1 (4T1 CM) cells for 72 h prior to medium conditioning. Before lysate collection, BMDMs were stimulated for 24 h with 100 ng/mL LPS. Flow cytometry analysis of phenotypic markers was performed. (**A–C**) Results are presented as MFI from 3 independent replicates. Real-time PCR analysis of *Il-23*, *Il-6*, *Tgfb*, *Tnfa*, and *Irf4* was performed. (**D–H**) RQ parameter was calculated using BMDMs from the 1000 IU group as a reference sample in each CM treatment. *Gapdh* was used for analysis as an endogenous control, *N* = 3*.* Statistic: Sidak’s multiple comparisons test; *P < 0.05, **P < 0.01.

To investigate how 4T1 tumor cells influence the differentiation of BMDMs from mice with different VD_3_ status, BMDMs were differentiated toward macrophages in the presence or absence of 4T1 CM. The phenotype of BMDMs isolated from healthy BALB/c mice did not differ significantly among treatment groups (Fig. 6A–C and Suppl. Fig. S6A–D, graphs described as control). When such BMDMs were treated during differentiation with CM from 4T1 cells nontreated (4T1 CM) or treated with 100 nM calcitriol (4T1cal CM), we did not observe any significant changes in the expressions of MHCII, CD44, CD80, and CD204 (Suppl. Fig. S6A–D). Increased expression of CD163 after culture with 4T1 CM was observed in the 1000 IU + cal and 100 IU + cal BMDMs groups (Fig. 6A). Moreover, 4T1cal CM decreased the expression of F4/80 and CD11b on BMDMs from all treatment groups compared with BALB/c mice fed 1000 IU of VD_3_ (Fig. 6B and C). Representative histograms are presented in Supplementary Fig. S7.

### Selected gene expression in BMDMs derived from healthy mice being on varied VD_3_ diets and treated with calcitriol cultured ex vivo with the 4T1 culture supernatants

Such differentiated BMDMs were also subjected to qPCR analysis of the expressions of *Il23*, *Il6*, *Tgfb*, *Tnfa*, *Irf4* (Fig. 6D–H), *Mrc1*, *Ifng, Arg1*, and *Fn1* (Suppl. Fig. S6E–H). Control BMDMs from healthy BALB/c mice did not differ significantly among treatment groups of mice. *Il23* expression was significantly increased in BMDMs from the 100 IU group stimulated with 4T1 CM, but the stimulation with 4T1cal CM led to an increase of *Il23* in the 100 IU + cal group compared with the 1000 IU, 1000 IU + cal, and 100 IU groups (Fig. 6D). BMDMs from the 100 IU + cal group of mice stimulated ex vivo with 4T1 CM and 4T1cal CM have increased the expression of *Il6* compared with 1000 IU, but 4T1cal CM stimulation increased its expression compared with all remaining groups (Fig. 6E). Stimulation with 4T1cal CM decreased *Tgfb* expression in BMDMs from group 100 IU + cal compared with 100 IU and 1000 IU + cal groups (Fig. 6F). The expression of *Tnfa* was the highest in BMDMs from the deficiency group incubated ex vivo with 4T1 CM, whereas *Tnfa* expression after incubation with 4T1cal CM was the highest in the 100 IU + cal group (*P* < 0.05 compared with 1000 IU) (Fig. 6G). *Irf4* (Fig. 6H), *Arg1*, *Mrc1*, *Ifng*, and *Fn1* (Suppl. Fig. S6E–H) gene expressions did not change significantly in these experimental conditions.

### Selected protein expression in BMDMs derived from healthy mice being on varied VD_3_ diets and treated with calcitriol cultured ex vivo with the 4T1 culture supernatants

**Figure 7:**
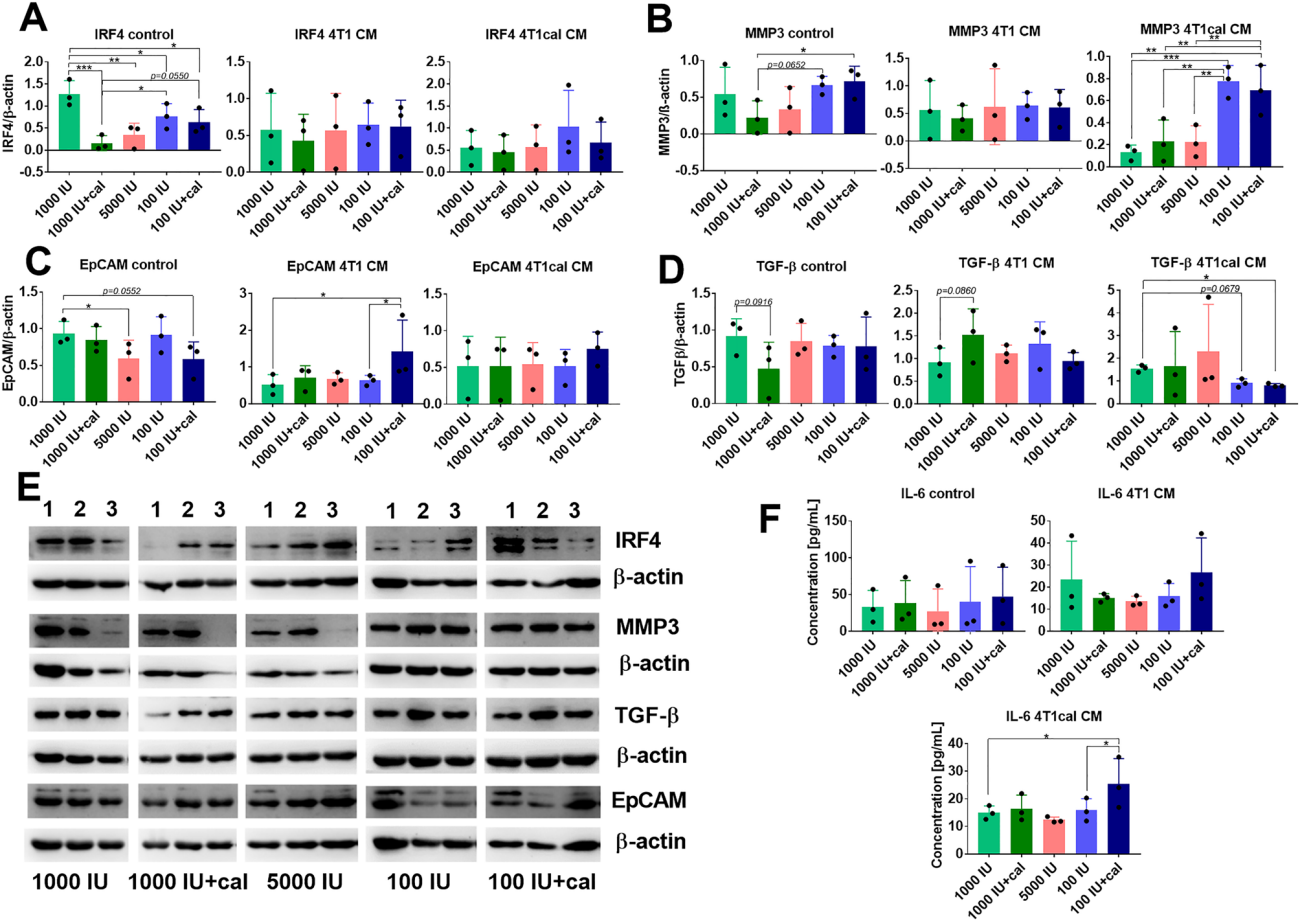
Expression of selected proteins in BMDMs cultured with 4T1 culture supernatants ex vivo. BMDMs were derived from healthy mice fed diets of varying contents of VD_3_ and treated with calcitriol. BMDMs were differentiated in the presence of 50 ng/mL M-CSF and 40% v/v CM-conditioned medium. CM was derived from 100 nM calcitriol-stimulated (4T1cal CM) or not (4T1 CM) 4T1 cells. Before lysate collection, BMDMs were stimulated for 24 h with 100 ng/nL LPS. (**A–E**) Western blot analysis (*N* = 3) of (**A**) IRF4, (**B**) MMP3, (**C**) EpCAM, and (**D**) TGF-β. (**E**) Representative blots; 1–control, 2–4T1 CM, and 3–4T1cal CM. (**F**) Concentration of IL-6 in BMDMs culture media supernatants. *N* = 3. Statistic: Sidak's multiple comparisons test; *P < 0.05, **P < 0.01, ***P < 0.001.

IRF4 protein expression was lower in all BMDMs groups from healthy mice fed various VD_3_ diets treated with control medium compared with 1000 IU group (normal), but it was not affected when BMDMs were treated with 4T1 or 4T1cal CM (Fig. 7A). The impact of various mice treatments on the MMP3 level in BMDMs was the highest in VD_3_ deficiency groups independent of calcitriol treatment. The highest expression of this protein was observed in 100 IU and 100 IU + cal groups when BMDMs were treated with 4T1cal CM (Fig. 7B). In control BMDMs, EpCAM expression was decreased in 5000 IU and 100 IU + cal groups, but when treated ex vivo with 4T1 CM, it increases significantly in 100 IU + cal group (Fig. 7C). In BMDMs treated with 4T1 CM, the highest expression of TGF-β was observed in 1000 IU + cal group. Significantly lower expression of TGF-β was observed in the 100 IU + cal group when BMDMs were treated with 4T1cal CM (Fig. 7D). Representative blots were presented in Fig. 7E. The expressions of PDIA3 and AP-1 did not differ significantly upon 4T1 CM or 4T1cal CM stimulation (Suppl. Fig. S6K and L).

Because the IL-6 level in 4T1 BMDMs was higher than IL-23, and *Il-6* gene was the most induced interleukin in 100 IU + cal group treated both with 4T1 CM and 4T1cal CM, we checked its secretion to supernatants and compared it with previously published results of IL-6 concentration in tumor tissue homogenates from 4T1 tumor-bearing mice^32^. The results revealed a correlation between mRNA expression and secretion status of IL-6 in the case of BMDMs from the VD_3_ deficiency group treated with calcitriol (100 IU + cal) and differentiated in 4T1cal CM (Fig. 7F). Moreover, tumor tissue homogenates showed a similar trend in IL-6 concentration for 100 IU + cal (554 ± 176 pg/mg) compared with 1000 IU (403 ± 94 pg/mg) and 100 IU (315 ± 33 pg/mg) treated mice (data in pg/mg of tumor tissue; *P* = 0.0869 and *P* = 0.0124, respectively, Sidak’s multiple comparison test, data presented in the supplementary file of our previous paper^32^). Consistent with gene analysis, these results demonstrated that the 100 IU + cal group was more susceptible than other groups to IL-6 induction by factor/factors secreted by calcitriol-stimulated 4T1 cells.

### Calcitriol-stimulated 4T1 cells show upregulated COX-2 protein level that directly translates into induced PGE_2_ secretion

**Figure 8:**
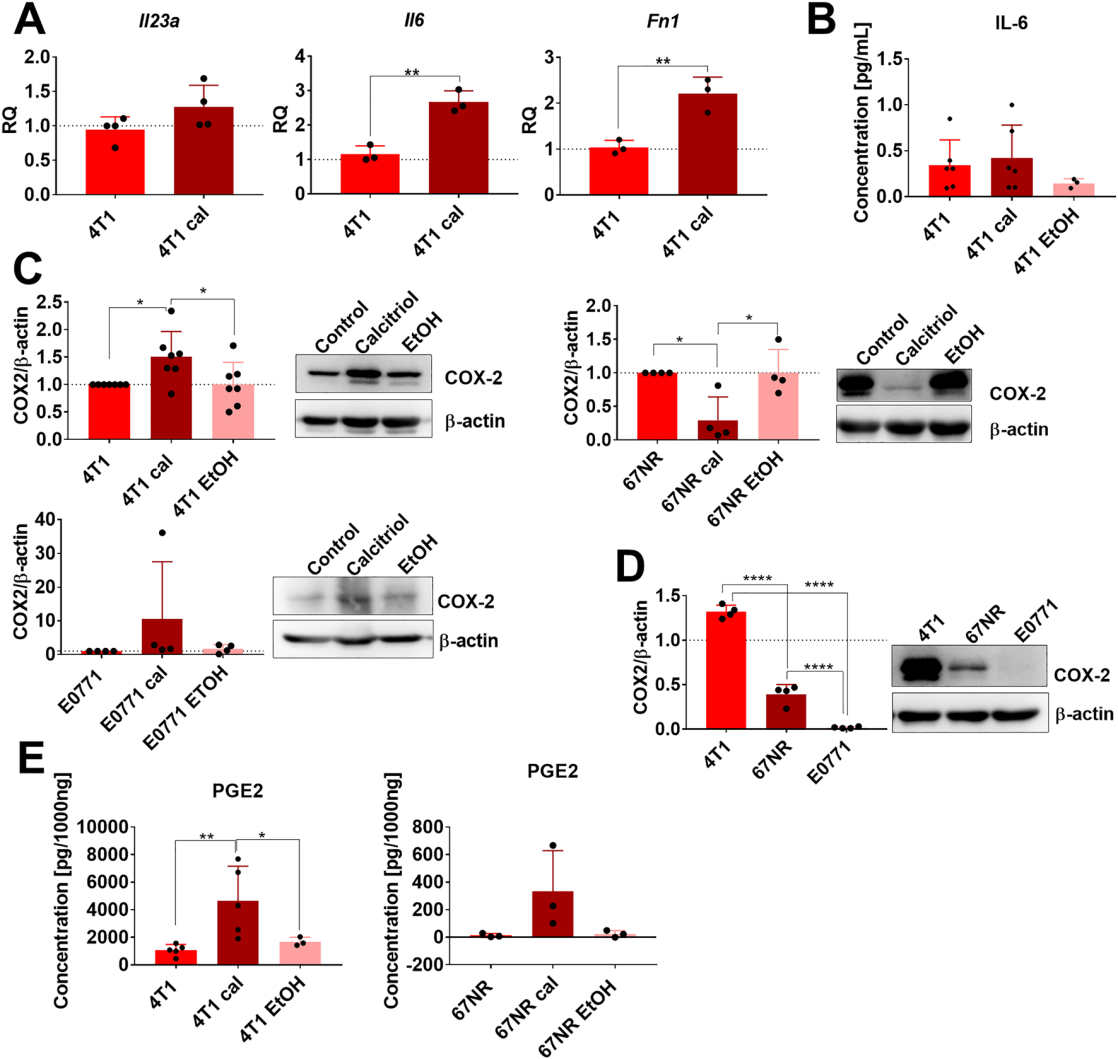
Characteristics of 4T1, 67NR, and E0771 mouse mammary gland cell lines on calcitriol treatment in vitro in the context of inflammatory factors expression. (**A**) Real-time PCR analysis of *Il23*, *Il6*, and *Fn1* gene expressions in 4T1 cells treated with 100 nM calcitriol for 72 h (4T1 cal) in vitro. Untreated cells served as the calibrator, and *Hprt* was used as an endogenous control, *N* = 3 or 4. (**B**) IL-6 concentration using ELISA in culture media from 4T1 cells treated with 100 nM of calcitriol for 72 h and then additionally treated with FBS free medium for 24 h. (**C**) Western blot analysis of cyclooxygenase-2 (COX-2) in 4T1, 67NR, and E0771 cells treated with 100 nM of calcitriol for 72 h. (**D**) Comparison of COX-2 expression between the three mouse mammary gland cancer cell lines (western blot). (**C**) and (**D**) Next to the graphs, representative blots are presented. (**E**) Prostaglandin E2 (PGE2) concentration using ELISA in culture media from 4T1 cells treated with 100 nM of calcitriol for 72 h and then additionally treated with FBS-free medium for 24 h. Statistical analysis: (**A**) Unpaired *t*-test; (**B**), (**D**) and (**E**) Sidak’s multiple comparisons test; (**C**) Dunn's multiple comparisons test; *P < 0.05, **P < 0.01.

Considering the hypothesis that calcitriol stimulates 4T1 cells to secrete factors inducing pathological pro-inflammatory responses in macrophages, we decided to broaden their characterization. Results revealed that 100 nM calcitriol treatment of 4T1 cells induced the expressions of *Fn-1* and *Il-6* genes, whereas *Il23a* remained unchanged (Fig. 8A). No significant differences were observed in IL-6 secretion. Furthermore, IL-6 concentration was very low (Fig. 8B). Interestingly, COX-2 protein levels increased on calcitriol treatment of 4T1 cells, decreased in 67NR cells, and remained unchanged in E0771 cells (Fig. 8C). Additionally, 4T1 cells had the highest COX-2 expression compared with 67NR and E0771. In E0771 cells, the expression of COX-2 was very low (Fig. 8D). Therefore, culture media were checked for PGE-2 content. Calcitriol-stimulated 4T1 cells secreted 4 times more PGE-2 than control and vehicle-treated cells. In 67NR cells, calcitriol did not significantly affect PGE2 expression (Fig. 8E).

COX-2 expression was also analyzed in tumor tissue; however, the treatment used did not significantly affect its expression. In control mice, the highest expression of COX-2 was noticed in 67NR tumors, and the expression of COX-2 did not differ significantly between 4T1 and E0771 tumors (Suppl. Fig. S8).

### Clinical significance of the COX-2 breast tumor tissue level in correlation with VD_3_-metabolizing enzymes in BC patients

**Figure 9:**
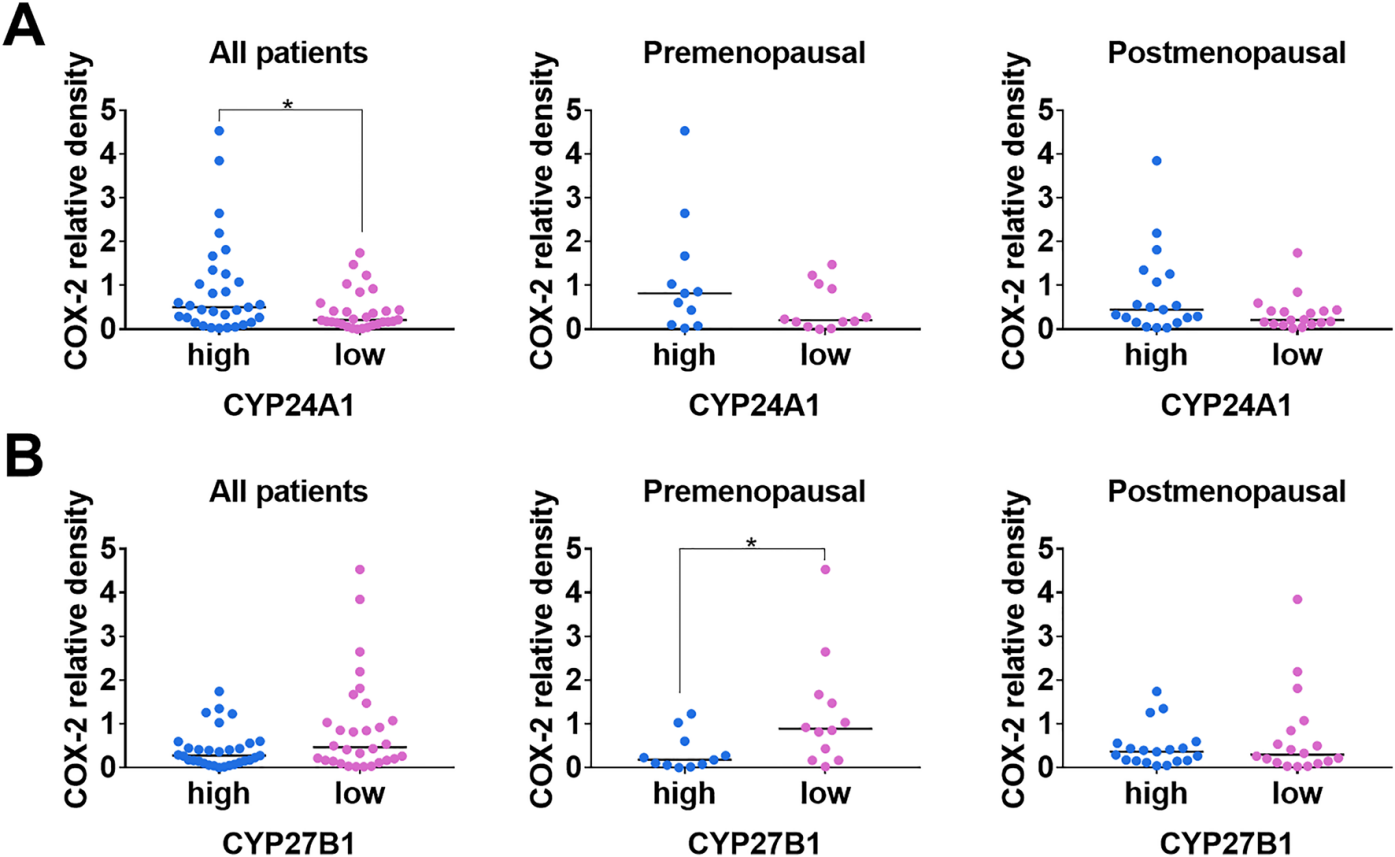
COX-2 expression in tumor tissue of BC patients. (**A**) Dependence of COX-2 expression on the CYP24A1 level. (**B**) Dependence of COX-2 expression on the CYP27B1 level. The patients were divided into groups based on menopausal status (determined by plasma FSH level: FSH > 25.8 mIU/mL postmenopausal; FSH < 25.8 mIU/mL premenopausal), vitamin D status (normal: 25(OH)D_3_ ≥ 30 ng/mL, deficient: 25(OH)D_3_ < 30 ng/mL plasma level), and tumor CYP24A1 or CYP27B1 expression. Protein expression was analyzed by western blot. The median value of densitometric analysis (protein/β-actin) was used as the criterion to divide the groups into high and low enzyme levels. Graphs depict the relative density of COX-2/β-actin in various groups of patients. Statistical analysis: Mann–Whitney test, *P < 0.05.

Tumor tissue from 60 BC patients was analyzed (Table 2). COX-2 expression in tumor tissue was notably higher in tumors with high expression of CYP24A1, and this difference was statistically significant when considering all patients (Fig. 9A). Tumors from premenopausal patients with low levels of CYP27B1 expressed higher COX-2 levels compared with patients with low expression of this enzyme (Fig. 9B). We also explored the impact of VDR expression on COX-2 with observed a tendency (*P* = 0.0595) toward lower expression of COX-2 in premenopausal patients with low expression of VDR (Suppl. Fig. S9A). Plasma 25(OH)D_3_ level did not appear to influence the expression of COX-2 (Suppl. Fig. S9B) in tumor tissues. Supplementary Fig. S9C displays the western blot membranes of analyzed tumors. Uncropped western blot images for all analyzes presented in this work are included in Supplementary Figures.

## Discussion

Numerous experimental or clinical studies have suggested a potential correlation between VD_3_ deficiency and poor outcomes in BC patients^20,38,39^. Paradoxically, several reports have indicated that high plasma levels of 25(OH)D_3_ may not confer benefits for BC patients^22,23^. Moreover, findings from experimental studies conducted on the 4T1 tumor model have demonstrated that subcutaneous injections of calcitriol or its analogs into young mice fed a normal VD_3_ diet could enhance metastasis^25,40^. However, another study has shown that initiating calcitriol treatment before injecting 4T1 cells could reduce tumor metastasis^28^. Hence, we aimed to investigate how the growth and metastasis of various murine mammary gland cancers are influenced by varying dietary intake of VD_3_ and particularly the plasma levels of the VD_3_ metabolite, 25(OH)D_3_. In addition, we examined whether oral administration of calcitriol had similar effects when administered to normal or VD_3_-deficient mice^26^. In the 4T1 tumor model, we observed an increased metastatic spread to the lungs in both calcitriol-treated groups and the group of mice fed with a 5000 IU diet. This suggests that, in this tumor model, increased delivery of VD_3_ or calcitriol itself might enhance the metastatic process. Notably, such changes were not observed in the VD_3_ deficiency group^26^. Conversely, in the E0771 metastatic mammary gland cancer model, we did not observe any significant effects of various diets or calcitriol treatment on lung metastases. However, there was a tendency toward decreased tumor growth following calcitriol treatment. In the non-metastatic 67NR model, which is isogenic to 4T1^41^, we only observed temporary tumor growth retardation with calcitriol treatment (1000 IU + cal)^26^. As demonstrated in our studies, all cell lines used in vitro responded to calcitriol treatment by increasing *Cyp24a1* expression. Confirmation of this effect at the protein level was observed in the 4T1 and 67NR cell lines and not in E0771. However, calcitriol treatment led to increased expression of VDR in E0771 cells. Notably, both 4T1 and E0771 showed higher mRNA expressions of *Cyp24a1*, *Cyp27b1*, and lower *Vdr* expressions compared with 67NR. Furthermore, 4T1 and E0771, but not 67NR, displayed resistance to the antiproliferative action of calcitriol^25,26^. However, regarding metastatic properties, calcitriol-induced metastasis to the lungs and bones appears to be specific for 4T1 in vivo model. Therefore, in further studies, we focused on characterizing the TME, particularly in the context of tumor models.

Macrophages constitute a main component of the TME, and we investigated their phenotype to assess polarization. In mice bearing 4T1 cells, fed a high VD_3_ diet, or treated with calcitriol, we observed a decrease in MHCII, which is characteristic of M1 polarization, along with an increase in markers typical of M2 macrophages. Notably, we observed an elevation in the surface protein CD44 in groups with a VD_3_-elevated diet or calcitriol treatment. CD44 serves as a receptor that plays a crucial role in macrophage phagocytosis^42^, its increased expression is often associated with the macrophage polarization toward the M2 phenotype^43^. CD44 is overexpressed in various cancer types and is linked to poor prognosis and increased potential of cancer cells to adhere to bone marrow endothelial cells^44,45^. Furthermore, CD44 serves as a receptor for several ligands, including OPN, a protein known to promote BC progression^46^ and induce M2 polarization in macrophages^47^. Interestingly, we observed an elevated level of OPN in lysates from whole 4T1 tumors in calcitriol-treated mice, suggesting its significant role in the process of metastasis of these tumor cells under the influence of VD_3_ and TAMs as the main effector cells. This finding may provide an explanation for an increased presence of metastatic foci in the bone marrow of these mice.

Our analysis of gene expression in TAMs or BMDMs from 4T1 tumor-bearing mice revealed the induction of several genes associated with alternative macrophage genotype under the influence of calcitriol, and notably, this effect was less pronounced in cells derived from 67NR mice. TAMs derived from mice fed an elevated VD_3_ diet or treated with calcitriol exhibited high *Il23* (also observed in 67NR-bearing mice) and *Ccr2* expressions. IL-23 is a proinflammatory cytokine in the TME that mediates the infiltration of M2 macrophages and neutrophils while inducing immunosuppression by reducing the infiltration of CD4^+^ and CD8^+^T cells into tumor tissues^48^. Additionally, CCR2 expression was elevated in these TAMs. CCR2 is expressed by macrophages with potent proinflammatory functions; paradoxically, in the TME, such macrophages can exhibit strong immunosuppressive properties^49^. Furthermore, we observed increased expressions of nitric oxide synthase 2 (*Nos2*), arginase 1 (*Arg1),* FOS like 1, AP-1 transcription factor subunit (*Fosl1*), C–C motif chemokine ligand 1 (*Ccl1*), and *Cd163* especially in mice from 100 IU + cal group. Although *Cd163*, *Ccl1*, and *Arg1* are considered characteristic of M2 macrophages, *Nos2* acts opposite^50,51^, and *Fosl1* (Fra-1) is known to decrease *Arg1* while increasing *Nos2*^52^. Despite the increase in *Nos2* mRNA in TAMs, BMDMs from 4T1 tumor-bearing mice did not exhibit significant differences in NO production between the groups of mice (Suppl. Fig. S10). Moreover, BMDMs derived from mice bearing 4T1 tumors displayed elevated expressions of *Il23* and interferon-regulatory factor 4 (*Irf4*) genes in groups treated with calcitriol. Transcription factor IRF4, induced by IL-4, favors M2 macrophage phenotype^53^. Furthermore, *Il6* expression increased in BMDMs from the 1000 IU + cal group compared with the 1000 IU group. The highest level of the *Mrc1* gene (the gene encoding the CD206 protein, a marker of M2 macrophages^35^) was observed in the 100 IU + cal group. In addition, an increase in *Fn1* (encoding fibronectin 1) was noticed in this group of mice, suggesting a profibrotic polarization of macrophages^54^. All of these findings led to the conclusion that calcitriol, especially in mice fed a VD_3_-deficient diet and treated with calcitriol (100 IU + cal), leads to M2 polarization of TAMs or BMDMs, consequently increasing the metastatic potential of 4T1 cells to bone marrow and lungs^26^. M2-like macrophages in tumor tissue can also induce angiogenesis^50^. In our previous studies, on the same mice from which TAMs and BMDMs are harvested, we observed decreased blood flow that may express a less organized, chaotic nature of blood vessels in 100 IU + cal group^32^. Moreover, it is accepted, that M2 polarization is the effect of the activity of Th2-derived cytokines^50^. A similar conclusion is also supported by our studies, in which calcitriol or its analogs increased metastasis and Th2 response^55^ and by Cao et al. in which calcitriol enhanced 4T1 tumor growth and increased Th2 response^56^.

The conclusions mentioned above were drawn from analyses conducted on cells (macrophages) obtained from tumor-bearing mice. However, due to indications from the analysis of BMDMs and bone marrow (*Epcam* expression, presence of metastases) suggesting the possibility of the cancer cell presence or their remnants in the material (gene expression studies), further investigations were carried out using BMDMs isolated from healthy mice and differentiated in the presence of CM from 4T1 cells stimulated with calcitriol. Differentiation of BMDMs (derived from healthy mice fed with a varied VD_3_ content and treated with calcitriol) in the presence of a 4T1 CM did not significantly affect the phenotype of macrophages. However, on analyzing the specific genes selected based on screening analyses, it became evident that the CM from 4T1 cells cultured with calcitriol (4T1cal CM) strongly stimulated the expression of genes encoding pro-inflammatory factors like TNFα, IL-6, and IL-23. These cytokines play fundamental roles in mediating tumor-promoting chronic inflammation within the TME and may act as diagnostic or prognostic markers^57–59^. It was also reported that TNFα secreted by macrophages can inhibit VDR expression in cancer cells, whereas upregulation of VDR decreases the metastatic potential of 4T1 cells^28^.

**Figure 10:**
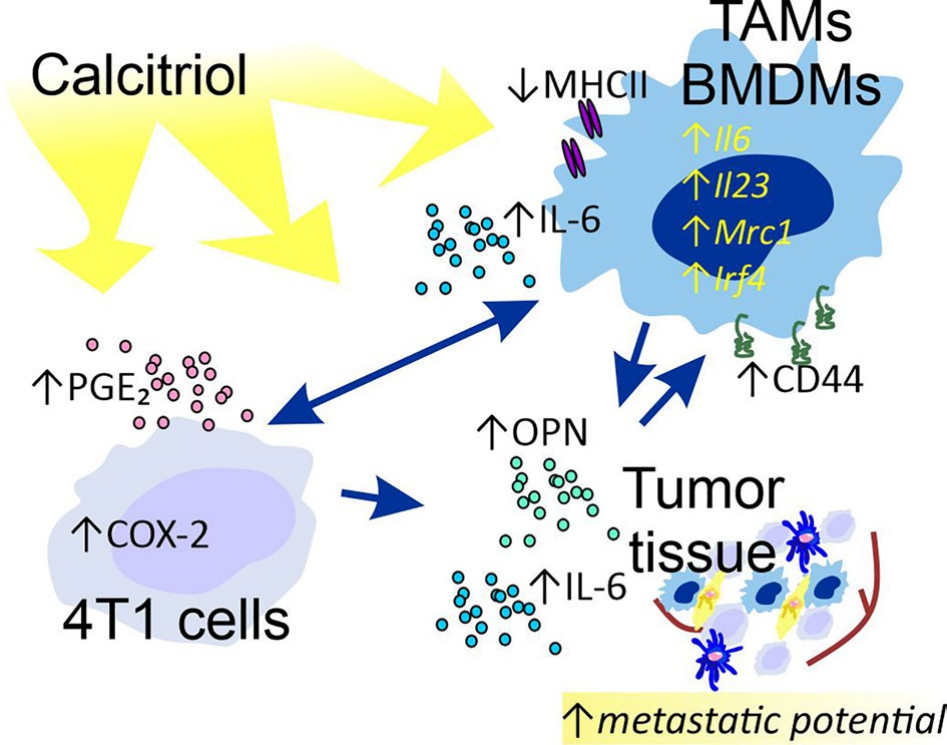
Proposed mechanism for the induction of invasive potential in 4T1 mammary gland cancer by calcitriol.

Furthermore, IL-6 secretion was stimulated by 4T1cal CM in the case of BMDMs from the VD_3_ deficiency group treated with calcitriol, and its concentration was also elevated in this group of mice in tumor tissue^32^. This finding provides evidence that these cells (from a 100 IU + cal group of mice) are more sensitive to the factors secreted by 4T1 cells upon stimulation with calcitriol. Our subsequent investigations revealed that 4T1 cells treated with calcitriol in vitro increased COX-2 expression and secreted higher levels of PGE_2_ into the culture media compared with control cells. PGE_2_ directly promotes M2 polarization via the cyclic AMP-responsive element binding (CREB)-mediated induction of Krupple-like factor 4 (KLF4)^60^. Furthermore, COX-2/PGE_2_ induces the release of IL-6 by macrophages and facilitates EMT in BC cells^61^. A study by Radharani et al. demonstrated that M2-activated RAW264.7 macrophages, in response to 4T1 CM, secreted elevated levels of IL-6. IL-6 derived from RAW264.7 further enhanced the metastatic properties of 4T1 cells in vitro and promoted tumor progression in vivo by inducing stem cell-related transcription factor expression through the STAT-3 pathway^62^. Additionally, it has been reported that IL-6 can reduce the antitumor activity of vitamin D in triple-negative BC (TNBC) cells overexpressing VDR in vitro. Moreover, when the TNBC cell lines were incubated with a combination of calcitriol and IL-6, they significantly downregulated E-cadherin gene expression and increased CD44^+^ cells compared with cells treated with calcitriol alone^63^. Furthermore, IL-6 trans-signaling contributes to the upregulation of OPN in macrophages, which is also important in the crosstalk between macrophages and fibroblasts^64,65^. Additionally, OPN could upregulate the expression of IL-6 in human chondrocytes^66^. Calcitriol transcriptionally regulates the *Spp-1* (encoding OPN) gene expression^67^, thereby contributing to the increased expression of this protein observed in our studies within tumor tissue. Studies involving the 4T1 mouse mammary gland cancer model have demonstrated that tumor-secreted PGE2 induces IL-23 production in the TME, leading to Th17 cell expansion^68^. In our previous investigations, we reported that tacalcitol, an analog of calcitriol, induced metastatic progression of 4T1 cells and promoted Th17 response in young mice^69^. These molecules, whose expression/secretion is stimulated via VD_3_ in TAMs and/or BMDMs, such as IL-6, IL-23, TNFα, and IRF4, in turn, are the major drives of Th17 immune response and facilitate the maintenance of Th17 status^39^. These factors may contribute to the increased metastatic potential of 4T1 cells. Therefore, based on our studies, a hypothesis has emerged that calcitriol-stimulated 4T1 cells, by secreting PGE_2_, induce TAMs M2 polarization and IL-6 production, and this cytokine, in turn, promotes the invasive potential of cancer cells (Fig. 10).

The relationship between VD_3_ and PGE_2_ metabolism has been previously demonstrated, with an elevated COX-2 and a reduced VDR expression in cancer patients compared with healthy women^70^. However, our patient study has shown that low VDR expression is associated with low expression of COX-2 (in premenopausal patients). Conversely, we have also demonstrated that COX-2 expression is higher in breast tumors with elevated expression of CYP24A1, an enzyme responsible for VD_3_ catabolizing, whereas tumors with high CYP27B1 expression (enzyme responsible for VD_3_ synthesis) exhibit lower COX-2 expression. It can be postulated that high expression of CYP24A1 favors reduced levels of the active form of VD_3_, calcitriol, within the tumor. Conversely, high expression of an enzyme directly involved in the synthesis of calcitriol will favor its high levels, potentially leading to decreased COX-2 expression. Consequently, these results may indirectly suggest that a high level of calcitriol within the tumor reduces the expression of COX-2. This observation is somewhat in line with studies indicating that breast and ovarian patients diagnosed during winter had significantly lower serum levels of 25(OH)D_3_ while displaying higher serum levels of PGE2^70^. However, such a correlation between 25(OH)D_3_ plasma levels and COX-2 tumor tissue expression was not evident in our studies. Nevertheless, COX-2, CYP27B1, and CYP24A1 expressions have also been associated with the pathogenesis of human colorectal cancer linked to chronic inflammation. Brozek et al. proposed that COX-2 activity associated with tumor initiation might result from escaping the constraints of the CYP27B1/VDR system^71^. As we presented herein, two isogenic mouse BC cell lines, 4T1 and 67NR, exhibited opposite responses toward calcitriol treatment and increased and decreased COX-2 expressions, respectively. According to published data, 4T1 and E0771 cells have a triple-negative and basal-like phenotype, whereas 67NR, presenting of nuclear ERα, displays a mixed luminal/basal phenotype^31,37^. In addition to the ability to metastasize, these lines also differ, e.g. in p53 expression, E0771 expresses mutant p53, while 67NR and 4T1 are *p53*-null^31,33,37,72^. Consequently, our observations indicate that the impact of VD_3_ varies both among diverse patient groups and across different cell lines, underscoring its close association with cancer subtype. Friedrich et al., in in vitro studies on human BC cell lines MCF-7 and MDA-MB-231, demonstrate that calcitriol inhibited COX-2 protein expression in MDA-MB-231 cells, as well as *PTGS2* (encoding COX-2) mRNA expression in both cell lines. Furthermore, a combination of calcitriol and celecoxib (COX-2 inhibitor) exhibited a synergistic growth-inhibitory effect in those cancer cell lines^73^, consistent with findings in other studies^74^. On the contrary, treatment of human immortalized HaCaT keratinocytes with calcitriol increased PGE_2_ production and COX-2 protein expression. This effect of calcitriol was characterized by rapid action dependent on PKC and Src kinase activities. Based on this study, the authors concluded that “upregulation of COX-2 expression with the consequent increase in PGE_2_ synthesis may be one of the mechanisms explaining the Janus face of calcitriol as both a promoter and attenuator of cutaneous inflammation”^75^. However, in macrophages, calcitriol suppresses COX-2 expression upon LPS stimulation by inhibiting the Akt/NF-κB/COX-2 pathway^76^. Additionally, human lung fibroblasts (HFL-1) incubated with VD_3_, 25(OH)D_3_, and calcitriol significantly lowered PGE_2_ production but no effect on COX-2, TGFβ1, VEGF, or fibronectin expression^77^. These varying effects of VD_3_ on various cell types can contribute to the complexity of the overall response observed in the organism.

## Conclusions

There exists a relationship between the response of macrophages to specific factors secreted by calcitriol-stimulated cancer cells, and VD_3_ deficiency may render immune cells more susceptible to pathological activation in response to calcitriol treatment. Furthermore, our findings suggest that the detrimental effects of calcitriol activation in 4T1 cells may be attributed to the induction of COX-2 and PGE2. As a result, future investigations will focus on the COX-2/PGE_2_/IL-6 pathway as the primary mediator of calcitriol-stimulated pathological inflammation within the 4T1 TME. These findings hold significance, particularly in the context of the widespread use of VD_3_ supplementation among BC patients, many of whom exhibit VD_3_ deficiency.

### Supplementary Information

Supplementary Information.

Supplementary Figures.

## Data Availability

All data generated or analyzed during this study are included in this published article (and its supplementary information files).
