# Supplementary material for: Calcitriol promotes M2 polarization of tumor-associated macrophages in 4T1 mouse mammary gland cancer via the induction of proinflammatory cytokines
Source: Sci Rep. 2024 Feb 15;14:3778. doi: 10.1038/s41598-024-54433-x (PMC10866890; doi:10.1038/s41598-024-54433-x)
Supplement: Supplementary file 1 — Supplementary Information. [file 41598_2024_54433_MOESM1_ESM.pdf]

Supplementary information 1

Calcitriol promotes M2 polarization of tumor-associated macrophages in 4T1 mouse mammary gland cancer *via* the induction of proinflammatory cytokines

Martyna Stachowicz-Suhs[1], Natalia Łabędź[1], Artur Anisiewicz[1], Joanna Banach[1], Dagmara Kłopotowska[1], Magdalena Milczarek[1], Aleksandra Piotrowska[2], Piotr Dzięgiel[2], Adam Maciejczyk[3,4], Rafał Matkowski[3,4], Joanna Wietrzyk[1*]

[1]Department of Experimental Oncology, Hirszfeld Institute of Immunology and Experimental Therapy, Weigla 12, 53-114 Wroclaw, Poland; martyna.stachowicz@hirszfeld.pl, natalia.labedz@hirszfeld.pl, a.anisiewicz@captortherapeutics.com, joanna.banach@hirszfeld.pl, dagmara.klopotowska@hirszfeld.pl, magdalena.milczarek@hirszfeld.pl, joanna.wietrzyk@hirszfeld.pl

[2]Division of Histology and Embryology, Department of Human Morphology and Embryology, Faculty of Medicine, Wroclaw Medical University, Chałubińskiego 6a, 50-368 Wroclaw, Poland; aleksandra.piotrowska@umw.edu.pl, piotr.dziegiel@umw.edu.pl,

[3]Department of Oncology, Wroclaw Medical University, Pl. Ludwika Hirszfelda 12, 53-413 Wrocław, Poland; rafal.matkowski@umw.edu.pl, adam.maciejczyk@umw.edu.pl

[4]Lower Silesian Oncology, Pulmonology and Hematology Center, Pl. Ludwika Hirszfelda 12, 53-413 Wrocław, Poland; matkowski.rafal@dco.com.pl, adam.maciejczyk@dco.com.pl

*Correspondence: joanna.wietrzyk@hirszfeld.pl; Tel. +48713709985

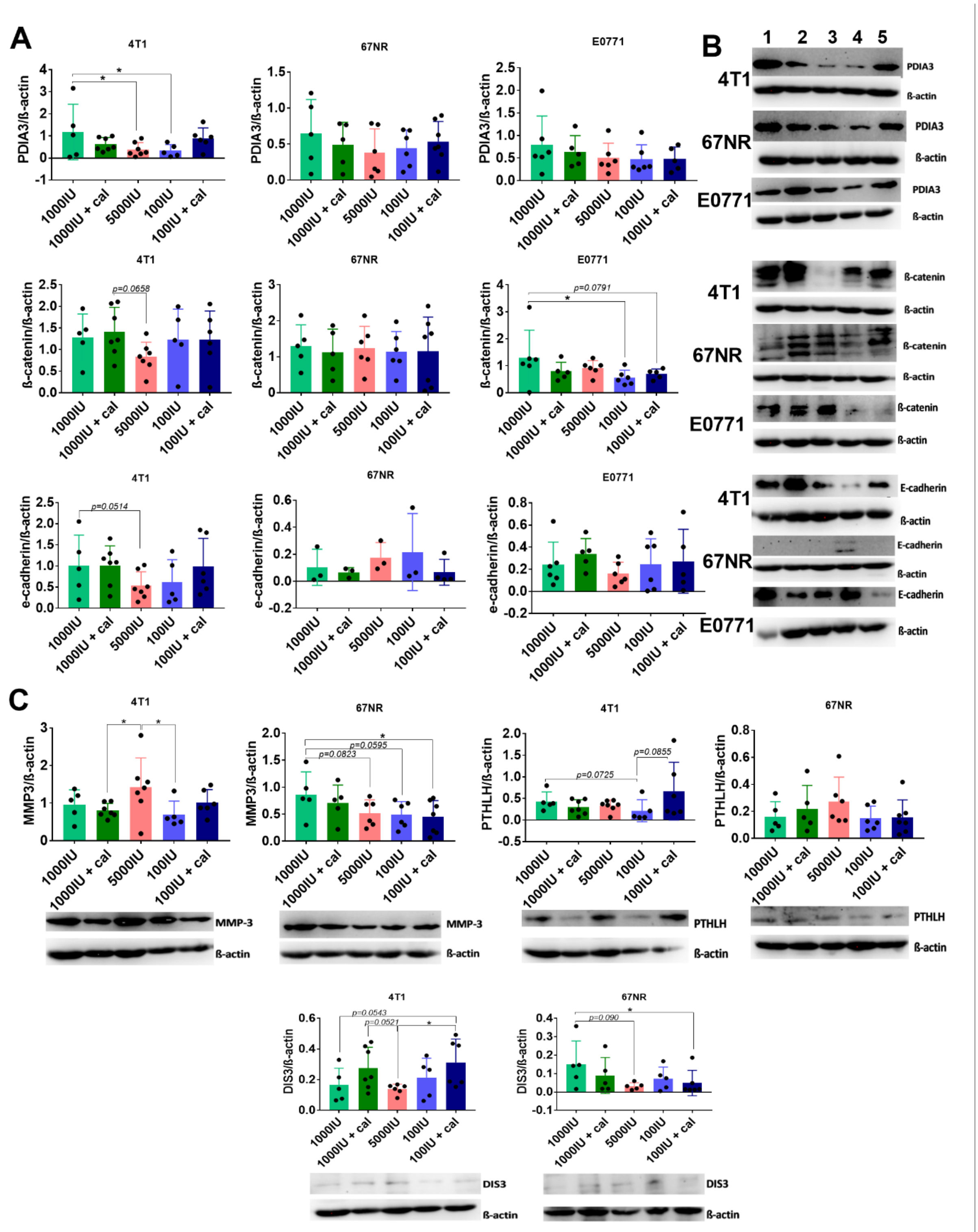

Figure S1. Western blot analysis of tumor tissue according to the proteins typical for metastatic 4T1 or E0771 tumors as compared to non-metastatic 67NR. **A** Expression of PDIA3, β-catenin and E-cadherin in mice bearing 4T1, 67NR and E0771 tumors. **B** Representative blots for analysis of expression of PDIA3, β-catenin and E-cadherin in mice bearing 4T1, 67NR and E0771 tumors. **C** Expression and representative blots of MMP3, PTHLH, and DIS3 in mice bearing 4T1 and 67NR tumors. Mice were exposed to normal/control (1000 IU/kg), deficient (100 IU/kg), and elevated (5000 IU/kg) VD_3 diets. After 6 weeks, 4T1, 67NR, or E0771 cells were injected orthotopically (day 0). The diets were continued until the end of the experiment (day 28 for 4T1 and 67NR or 23 for E0771 tumor-bearing mice). Mice fed 1000 IU/kg and 100 IU/kg diets received additional oral calcitriol gavage starting 7 days after cancer cells inoculation (1 μg/kg; three times a week).

Tumor tissue homogenates were analyzed from 3-7 mice (as shown on graphs). Statistical analysis: Sidak's multiple comparison test; **P*<0.05, ***P*<0.01, ****P*<0.001, *****P*<0.0001.

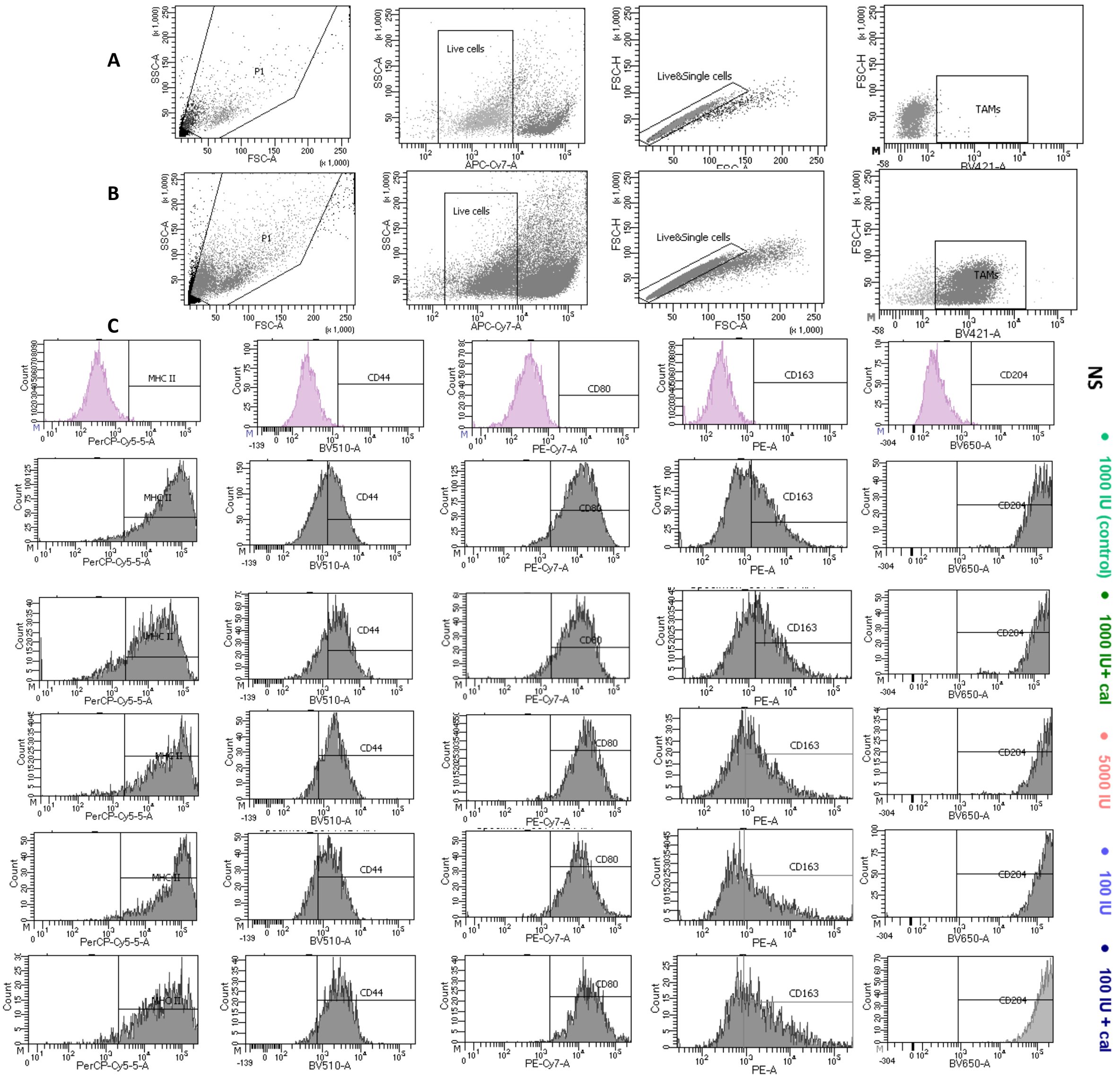

Figure S2. Gating strategy of TAMs and representative histograms. Presented results are obtained on TAMs isolated from 4T1 tumors. Gating strategy: **A** isotype control; **B** F4/80 positive cells. **C** Representative histograms showing MHCII, CD44, CD80, CD163, CD204 expression on TAMs isolated from 4T1 tumors. NS – not stained.

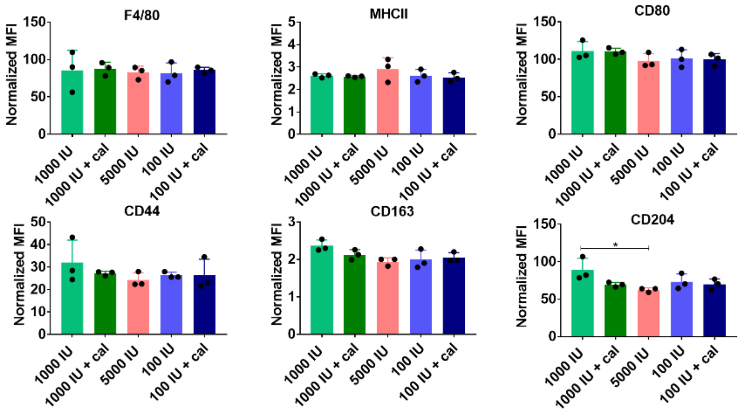

Figure S3. Phenotype analysis of BMDMs (7 days of differentiation with M-CSF next 24h stimulation with LPS) from mice bearing 4T1 tumors fed diets with varying VD$_3$ content and treated with calcitriol. Flow cytometry analysis was done using BMDMs differentiated in the presence of M-CSF. Expression of the following macrophage markers was analyzed: F4/80, MHCII, CD44, CD80, CD163, CD204. Data are presented as median fluorescence intensity (MFI); mean value with SD as well as data for individual mice are presented. N=3. Statistical analysis: Dunn's multiple comparisons test; **P*<0.05.

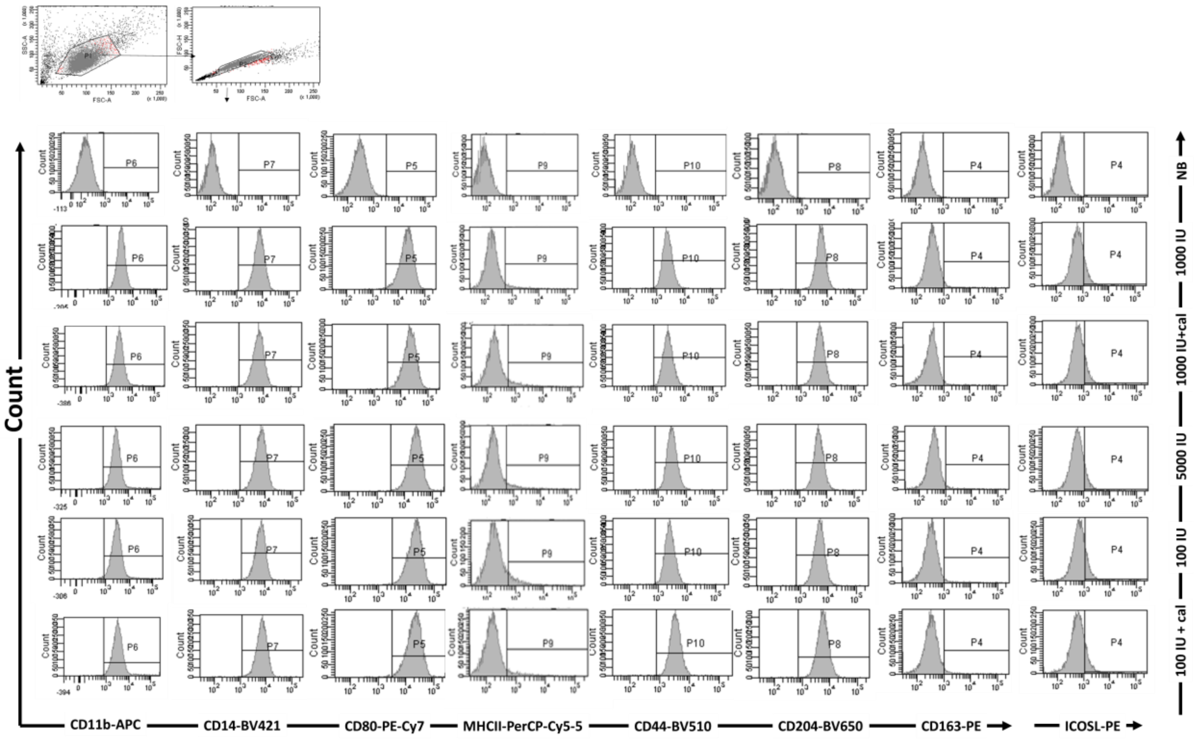

Figure S4. BMDMs from 4T1-bearing mice. Representative histograms.

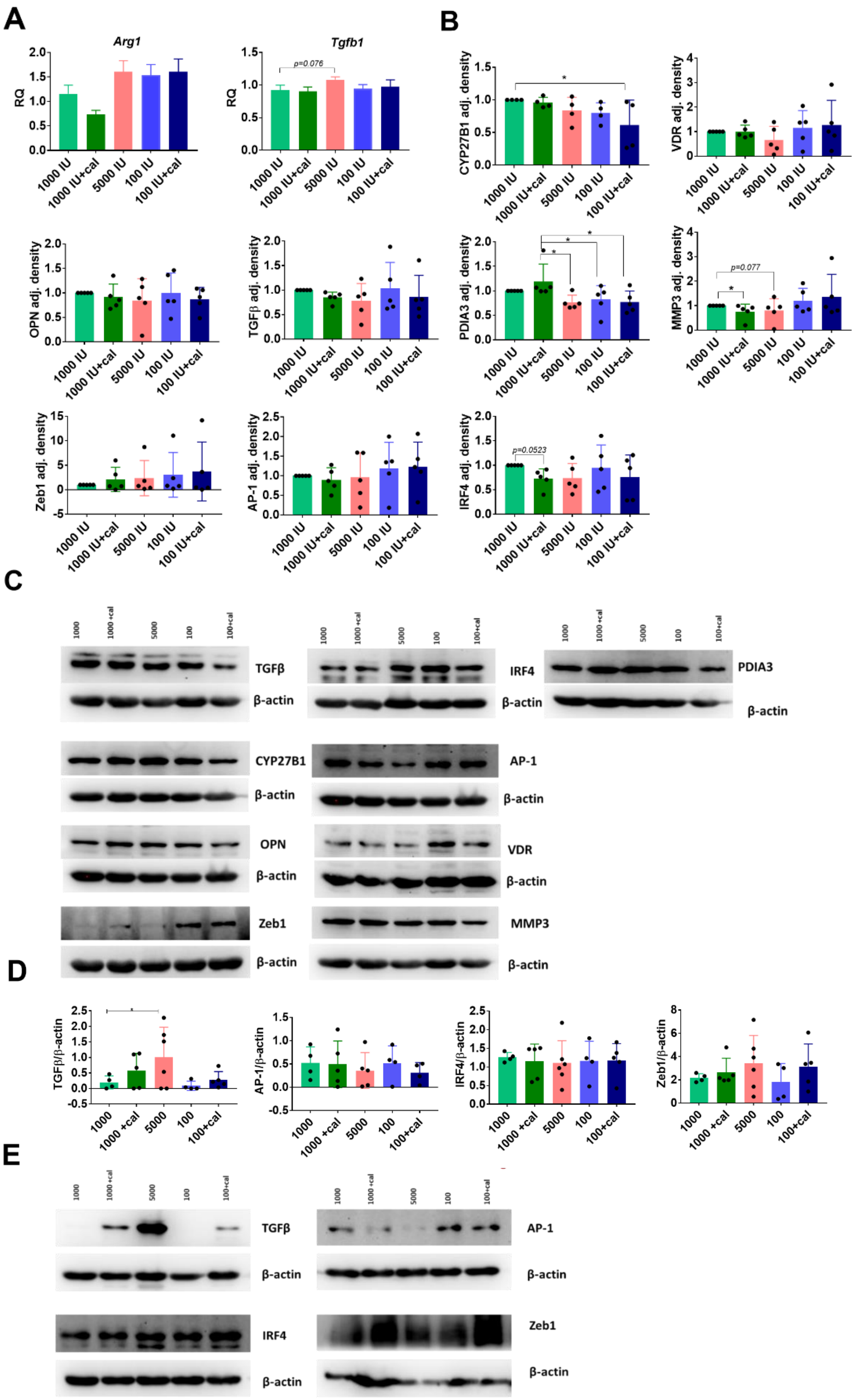

Figure S5. Gene and protein expression in BMDMs isolated from 4T1 tuomr-bearing mice. **A** Real-time PCR analysis of *Arg1* and *Tgfb1* in 4T1 BMDMs; N=3. **B** Western-blot analysis of CYP27B1, VDR, OPN, TGFβ, PDIA3, MMP3, Zeb1, AP-1 and IRF4 expression in 4T1 BMDMs; N=5. **C** Representative blots are presented. **D** Western blot evaluation of AP-1, IRF4, Zeb1, and TGFβ level in 4T1 tumor tissue homogenates. **E** Representative blots are presented. N=4-5. Statistical analysis: Dunn's multiple comparisons test; **P*<0.05, ***P*<0.01, ****P*<0.001, *****P*<0.0001.

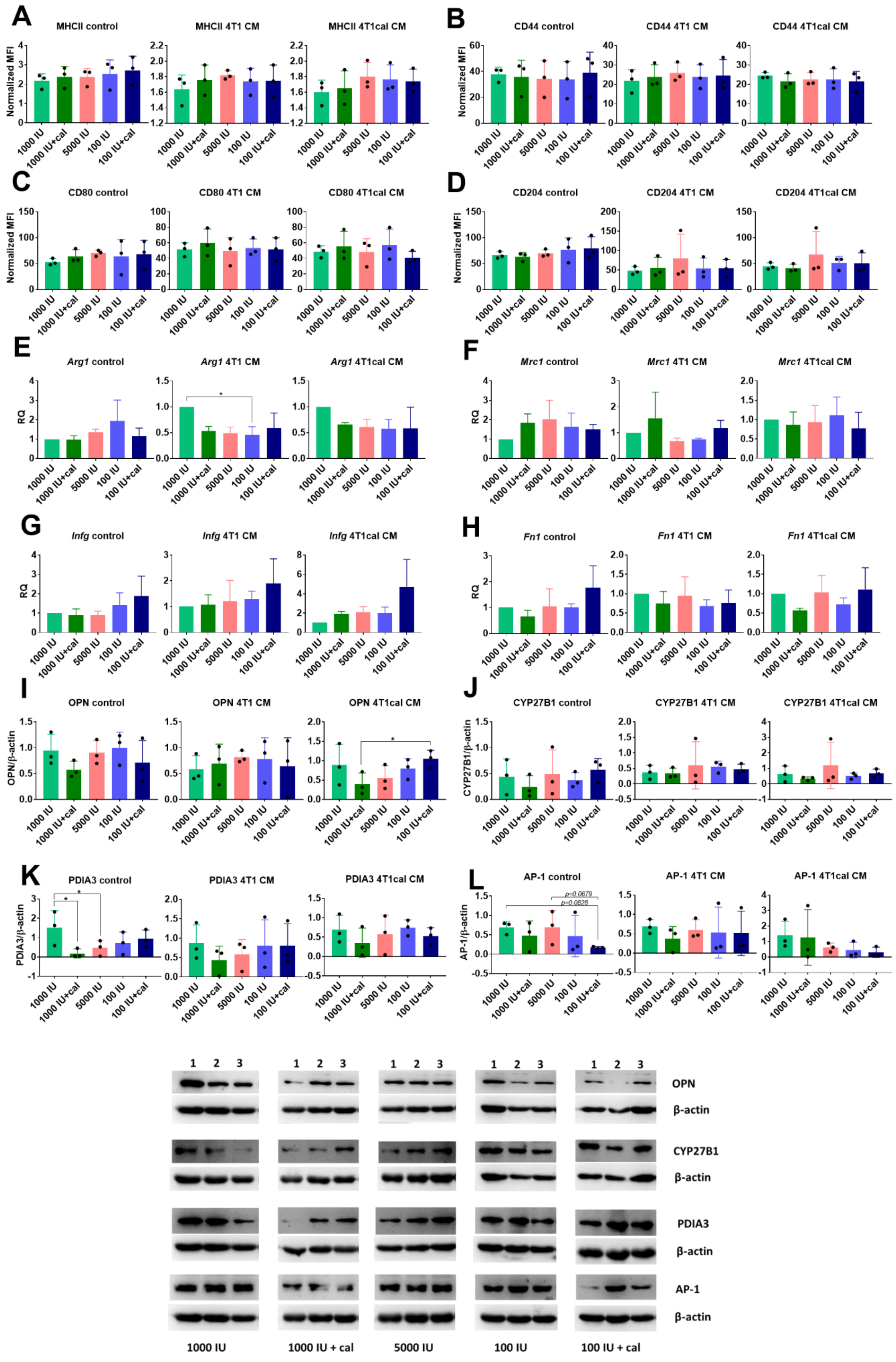

A
MHCII control
MHCII 4T1 CM
MHCII 4T1cal CM
B
CD44 control
CD44 4T1 CM
CD44 4T1cal CM
C
CD80 control
CD80 4T1 CM
CD80 4T1cal CM
D
CD204 control
CD204 4T1 CM
CD204 4T1cal CM
Normalized MFI
E
Arg1 control
Arg1 4T1 CM
Arg1 4T1cal CM
F
Mrc1 control
Mrc1 4T1 CM
Mrc1 4T1cal CM
G
Infg control
Infg 4T1 CM
Infg 4T1cal CM
H
Fn1 control
Fn1 4T1 CM
Fn1 4T1cal CM
RQ
I
OPN control
OPN 4T1 CM
OPN 4T1cal CM
OPN/β-actin
J
CYP27B1 control
CYP27B1 4T1 CM
CYP27B1 4T1cal CM
CYP27B1/β-actin
K
PDIA3 control
PDIA3 4T1 CM
PDIA3 4T1cal CM
PDIA3/β-actin
L
AP-1 control
AP-1 4T1 CM
AP-1 4T1cal CM
AP-1/β-actin
p=0.0679
p=0.0828
1000 IU
1000 IU+cal
5000 IU
100 IU
100 IU+cal
1 2 3
OPN
β-actin
CYP27B1
PDIA3
AP-1
1000 IU
1000 IU + cal
5000 IU
100 IU
100 IU + cal

Figure S6. The characteristics of BMDMs cultured *ex vivo* with 4T1 culture supernatants. BMDMs were derived from healthy mice fed diets containing varying contents of VD_3 and treated with calcitriol. BMDMs were differentiated in the presence of 50 ng/mL M-CSF and 40% v/v CM-conditioned medium. CM was derived from 100 nM calcitriol-stimulated (4T1cal CM) or not-stimulated 4T1 (4T1 CM) cells for 72 h prior to medium conditioning. Before lysates collection, BMDMs were stimulated for 24 h with 100 ng/mL LPS. **A-D** Analysis of phenotypic markers was done using flow cytometry. Results are presented as MFI from 3 independent replicates. **E-H** Real-time PCR analysis of *Arg1*, *Mrc1*, *Infg*, *Fn1* was done. RQ parameter was calculated using BMDMs from the 1000 IU group as a reference sample in each CM treatment. *GAPDH* was used for analysis as an endogenous control, N=3. **I-L** Western blot analysis with representative blots of OPN, CYP27B1, PDIA3, AP-1. N= 3. Representative blots; 1 - control, 2 – 4T1 CM, 3 – 4T1cal CM. Statistic: Sidak's multiple comparisons test, **P*<0.05.

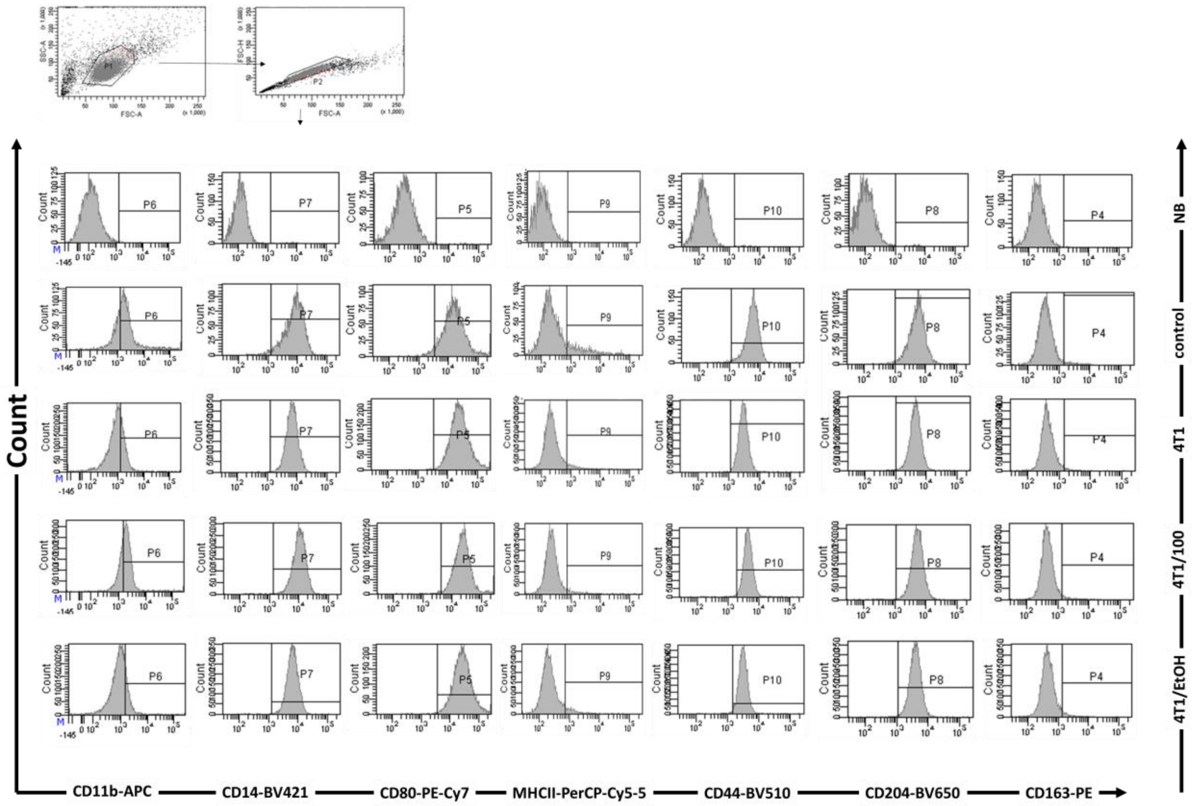

Figure S7. BMDMs from healthy BALB/c mice – example group 1000 IU.

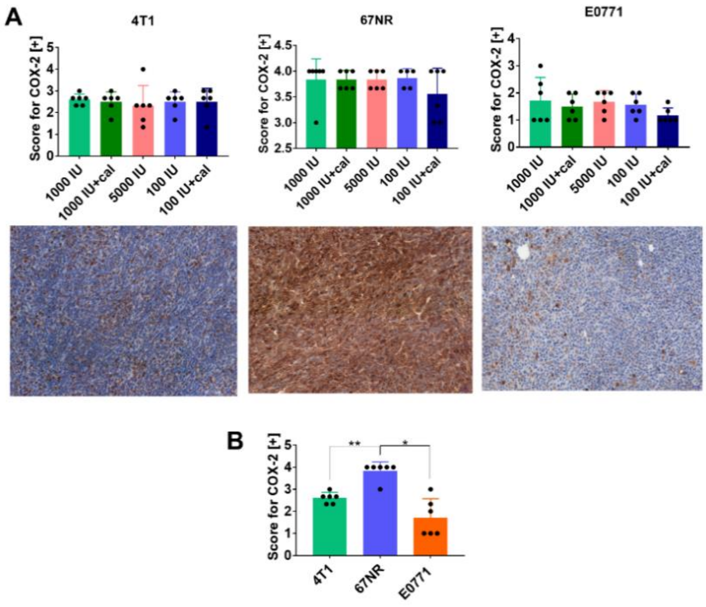

Figure S8. **COX-2 expression in tumor tissue from mice bearing 4T1, 67NR, and E0771 tumors. A** Score for COX-2 expression and representative images of COX-2 staining (below each graph). **B** Comparison of COX-2 expression in control 4T1, 67NR, and E0771 tumors. N=6. Statistical analysis: Dunn's multiple comparisons test; *$P<0.05$, **$P<0.01$.

Supplementary method description for Figure S8 results:

Tissue Microarrays (TMAs)

Tissue microarrays (TMAs) were prepared from formalin-fixed and paraffin-embedded tissue using the TMA Grand Master (3DHistech, Budapest, Hungary) automatic tissue microarrayer. Hematoxylin and eosin-stained sections were used to select representative sites by a pathologist. 3 representative cores with a size of 1.5 mm from the donor block were transferred to the TMA recipient block.

Immunohistochemistry (IHC)

Immunohistochemical reactions were performed on 4 µm paraffin sections obtained from TMA blocks using Autostainer Link48 (Dako, Glostrup, Denmark). In order to deparaffinize, rehydrate and unmask the epitopes the slides were boiled in EnVision FLEX Target Retrieval Solution (pH 9.0, at 97˚C, 20 min; Dako). Inactivation of the endogenous peroxidase was performed by incubation sections for 5 min with EnVision FLEX Peroxidase-Blocking Reagent (Dako). Anti-COX-2 antibody (1:100, cat. no. MA5-42821, ThermoFisher Scientific, Waltham, MA, USA) was used as primary antibody (20 min incubation, RT). In next step, the slides were incubated with EnVision FLEX/HRP (20 min, room temperature). 3,3'-diaminobenzidine (DAB) was utilized as the peroxidase substrate (10 min incubation, RT). Hematoxylin was used to visualize cell nuclei. Finally, slides were dehydrated in graded alcohol concentrations (70%, 96%, absolute) and xylene and were closed with coverslips in Dako Mounting Medium (Dako).

Evaluation of IHC reactions

Evaluation of IHC reactions was performed using BX41 Olympus microscope (Olympus Corporation, Tokyo, Japan). The intensity of the reaction of COX-2 antigen in cancer cells was based on the percentage of reaction-positive cells among cancer cells. The points determining the expression were as follows: 0 point - 0%; 1 point - ≤10%; 2 points - 11%-25%; 3 points - 26%-50%; 4 points - over 50% of the cells with positive reaction.

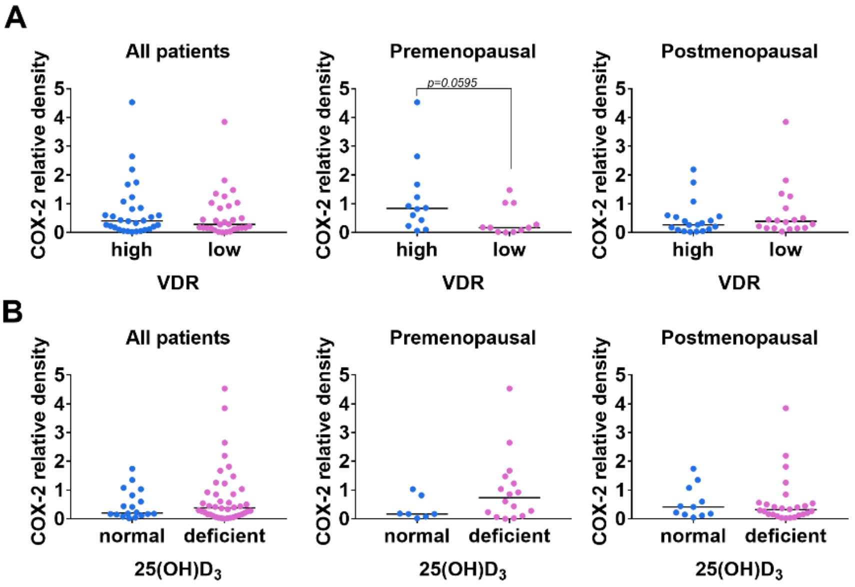
A
All patients
Premenopausal
Postmenopausal
COX-2 relative density
p=0.0595
high
low
VDR
B
normal
deficient
25(OH)D3

C

COX-2

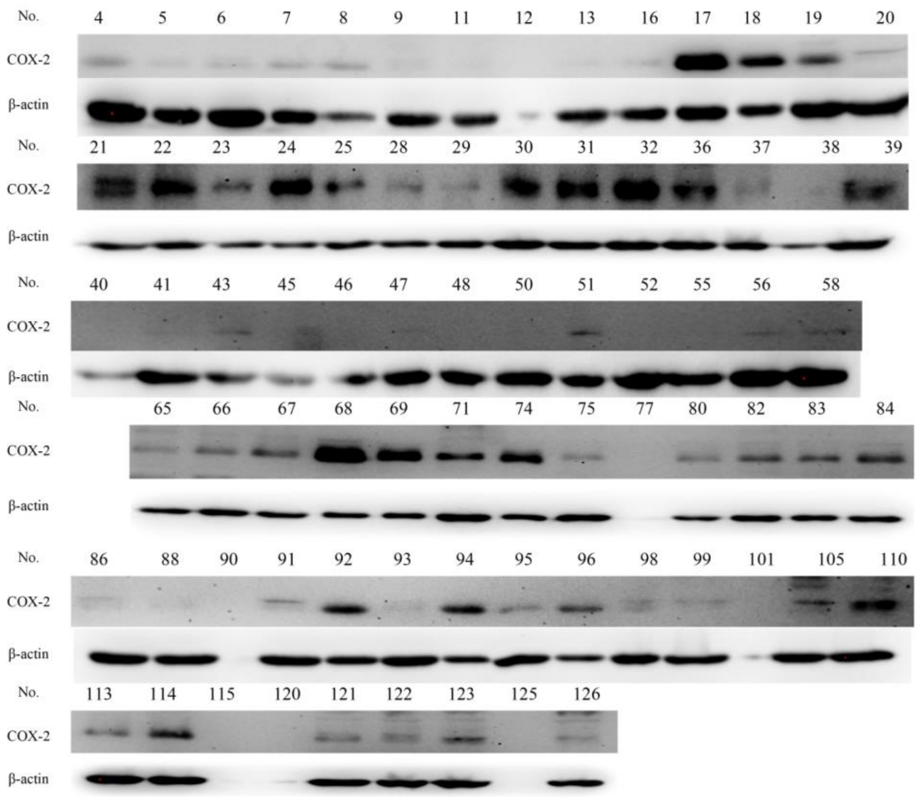
No.
4 5 6 7 8 9 11 12 13 16 17 18 19 20
COX-2
β-actin
21 22 23 24 25 28 29 30 31 32 36 37 38 39
40 41 43 45 46 47 48 50 51 52 55 56 58
65 66 67 68 69 71 74 75 77 80 82 83 84
86 88 90 91 92 93 94 95 96 98 99 101 105 110
113 114 115 120 121 122 123 125 126

VDR

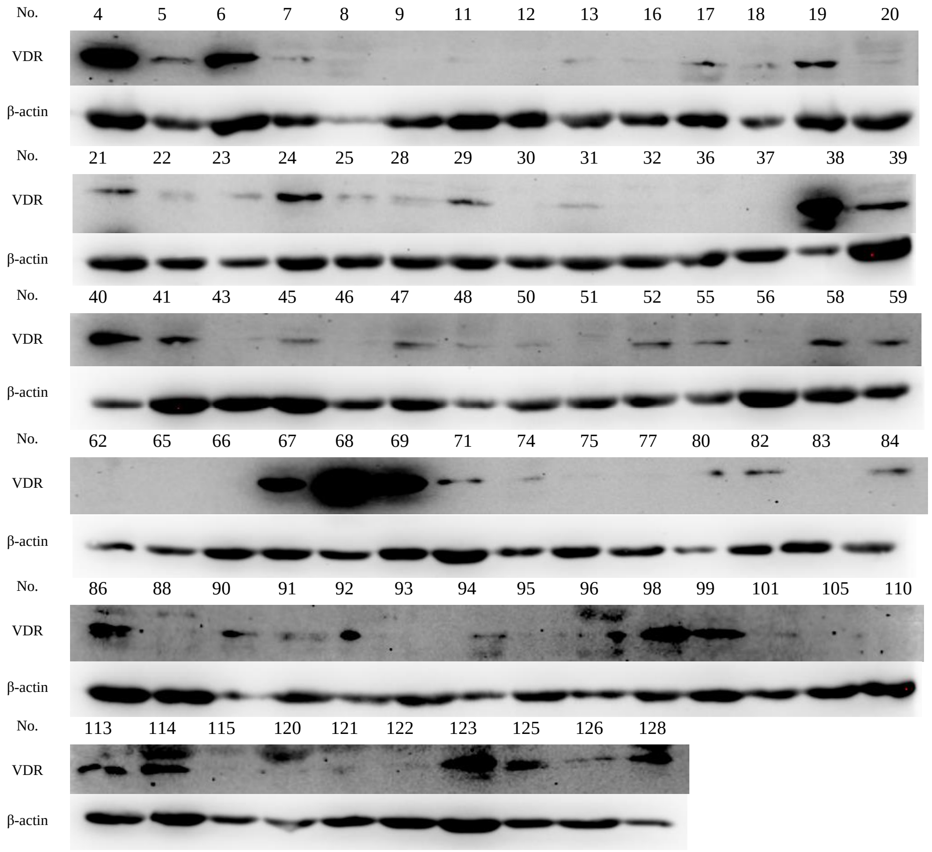
No. 4 5 6 7 8 9 11 12 13 16 17 18 19 20
VDR
β-actin
No. 21 22 23 24 25 28 29 30 31 32 36 37 38 39
VDR
β-actin
No. 40 41 43 45 46 47 48 50 51 52 55 56 58 59
VDR
β-actin
No. 62 65 66 67 68 69 71 74 75 77 80 82 83 84
VDR
β-actin
No. 86 88 90 91 92 93 94 95 96 98 99 101 105 110
VDR
β-actin
No. 113 114 115 120 121 122 123 125 126 128
VDR
β-actin

CYP24A1

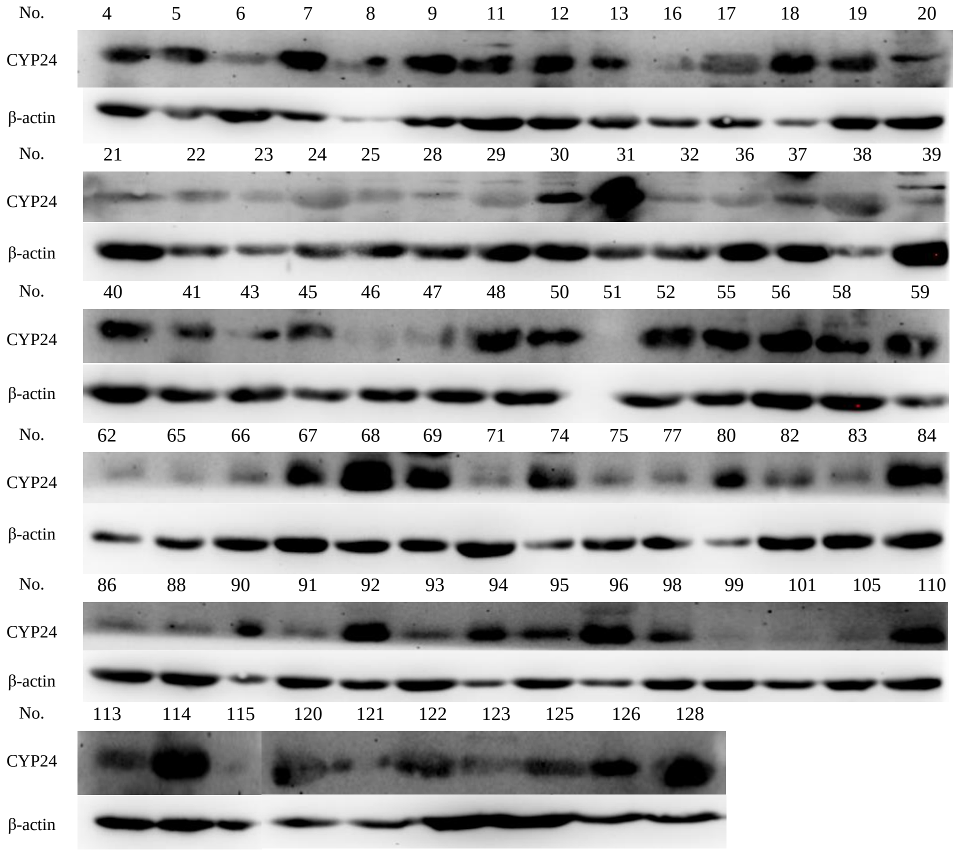
No. 4 5 6 7 8 9 11 12 13 16 17 18 19 20
CYP24
β-actin
No. 21 22 23 24 25 28 29 30 31 32 36 37 38 39
CYP24
β-actin
No. 40 41 43 45 46 47 48 50 51 52 55 56 58 59
CYP24
β-actin
No. 62 65 66 67 68 69 71 74 75 77 80 82 83 84
CYP24
β-actin
No. 86 88 90 91 92 93 94 95 96 98 99 101 105 110
CYP24
β-actin
No. 113 114 115 120 121 122 123 125 126 128
CYP24
β-actin

CYP27B1

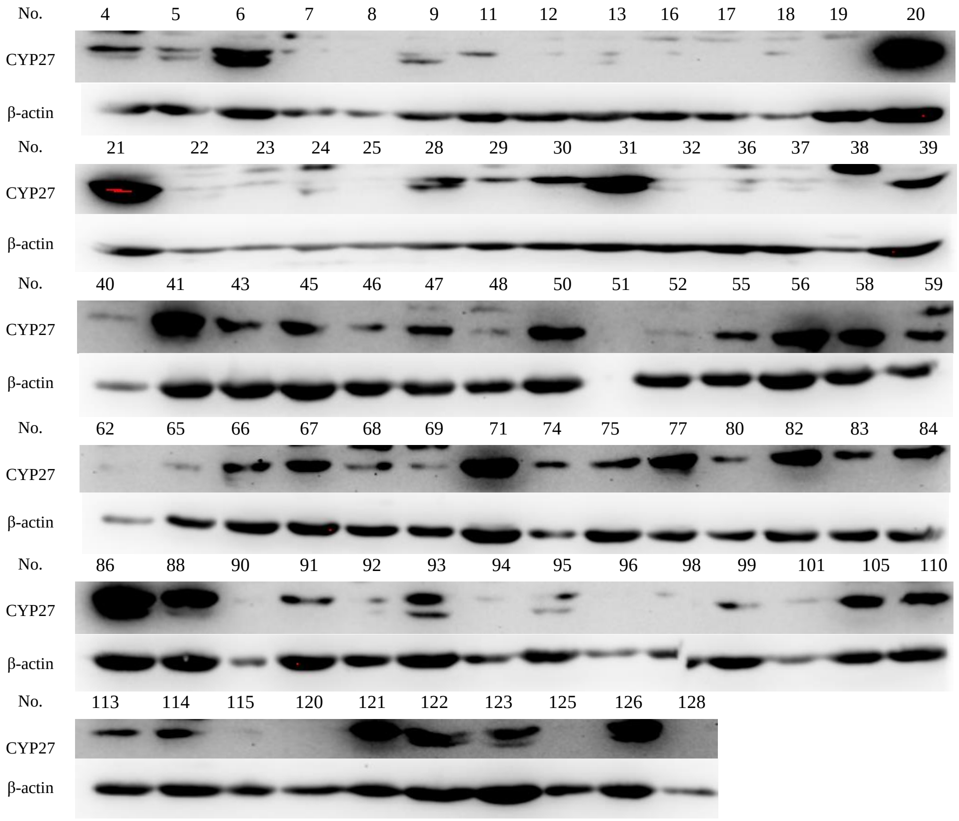

Figure S9. **COX-2 expression in cancer patients.** Western blot analysis. **A** Dependence of COX-2 expression on the VDR level. **B**. Dependence of COX-2 expression on the 25(OH)D3 plasma level. The patients were divided into groups according to the menopausal status (on the basis of plasma FSH level: FSH > 25.8 mIU/mL postmenopausal; FSH < 25.8 mIU/mL premenopausal), VD3 status (normal: 25(OH)D3 ≥ 30ng/mL, deficient: 25(OH)D3 < 30 ng/mL plasma level) and on the basis of VDR expression in tumor: the median value of densitometric analysis (VDR/β-actin) was arbitrarily chosen as dividing the group with high and low levels of VDR. **C** Images of membranes from western-blot analyzes of COX-2, VDR, CYP24A1, CYP27B1 of all examined patients. Statistical analysis: Mann Whitney test.

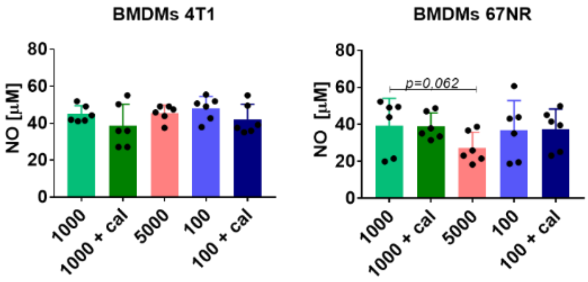

Figure S10. NO production by BMDMs derived from 4T1 and 67NR tumor-bearing mice. N=6. Griess diazotization reaction-based test was applied as described earlier by Porshneva et al. [1]. N=6. Statistical analysis: Dunn's multiple comparisons test.

Table S1. The expression of genes related to macrophage function and VD_3 mechanisms of action in TAMs isolated from 4T1 and 67NR tumors.

	RQ						
	4T1			67NR			
Target genes:	1000+cal	5000	100+cal	1000+cal	5000	100	100+cal
Nos2-Mm00440502_m1	1.724	1.698	2.606	1.237	0.787	0.685	0.605
Runx1-Mm01213405_m1	1.089	1.028	1.059	1.065	1.323	1	1.128
Nfkb1-Mm00476361_m1	1.328	1.007	1.011	1.53	1.681	1.48	1.459
Irf8-Mm00492567_m1	0.999	0.641	0.924	1.51	1.482	1.51	1.411
Irf3-Mm00516784_m1	0.659	1.011	0.898	0.706	1.138	0.757	0.914
Irf4-Mm00516431_m1	0.456	0.585	0.933	1.03	2.446	1.923	3.154
Irf5-Mm00496477_m1	0.632	0.572	0.421	1.188	1.528	1.697	1.906
Tlr4-Mm00445273_m1	1.138	0.577	0.925	1.334	1.318	1.646	1.64
Stat1-Mm00439531_m1	0.48	0.679	0.687	0.762	1.132	0.857	0.929
Stat3-Mm01219775_m1	1.722	1.12	1.157	1.751	1.654	1.648	1.308
Stat6-Mm01160477_m1	1.059	0.926	0.871	1.011	1.268	1.017	1.146
Pparg-Mm01184322_m1	0.55	0.649	0.493	0.746	1.987	1.182	1.471
Cebpb-Mm00843434_s1	0.68	0.648	0.522	1.679	1.341	1.903	1.909
Mrc1-Mm01329362_m1	2.829	0.942	0.546	0.672	1.046	0.99	0.505
Ifng-Mm01168134_m1	0.162	0.98	0.593	0.652	x	0.445	2.892
Myd88-Mm00440338_m1	0.687	0.659	0.781	1.142	1.363	0.913	1.17
Ticam1-Mm00844508_s1	0.691	0.751	0.826	0.745	1.874	1.022	1.298
Vegfa-Mm00437306_m1	1.816	1.706	1.65	0.648	0.64	1.128	1.014
Pdgfa-Mm01205760_m1	1.703	2.008	1.766	0.492	0.646	0.769	0.881
Hgf-Mm01135193_m1	1.602	0.372	1.017	0.791	0.573	0.914	1.47
Egf-Mm00438696_m1	0.6	0.593	0.827	0.593	1.058	0.718	0.708
Alox15-Mm00507789_m1	0.362	x	x	x	x	0.586	0.944
Clec10a-Mm00546124_m1	0.269	0.184	0.169	0.514	1.396	0.697	0.784
Mgl2-Mm00460844_m1	0.938	x	1.959	0.02	0.222	0.204	0.067
Fn1-Mm01256744_m1	1.649	2.42	2.284	0.689	1.369	2.229	2.218
Tnf-Mm00443258_m1	0.473	0.353	0.337	1.505	2.059	1.764	1.853
Tgfb1-Mm01178820_m1	0.509	0.673	0.574	0.695	1.452	0.868	0.928
Il10-Mm01288386_m1	0.537	0.652	0.469	1.256	2.056	1.473	1.294
Il12a-Mm00434169_m1	0.889	1.106	1.322	1.322	1.027	0.795	1.024
Il12b-Mm00434174_m1	0.466	0.232	x	1.025	0.985	1.097	1.702
Il23a-Mm00518984_m1	3.504	3.324	2.392	1.275	1.926	2.836	3.998
Mmp1a-Mm00473485_m1	2.618	4.587	2.531	0.813	x	0.634	0.998
Mmp2-Mm00439506_m1	0.857	1.484	1.891	0.492	0.805	0.82	0.772
Mmp7-Mm01168420_m1	x	1.564	1.149	x	x	0.752	x
Mmp9-Mm00442991_m1	1.173	1.999	2.029	0.981	1.433	2.158	1.202
Mmp14-Mm00485054_m1	1.106	0.453	0.728	2.39	1.634	1.204	1.808
Ptges-Mm00452105_m1	0.835	0.798	0.851	1.241	1.483	0.425	1.604
Arg1-Mm00475988_m1	1.301	1.387	2.368	0.757	0.325	1.43	0.433
Irak3-Mm00518541_m1	0.761	0.314	0.455	1.819	1.391	1.302	1.376

Hif1a-Mm00468869_m1	1.652	1.204	1.552	1.508	1.082	1.005	1.408
Ccl1-Mm00441236_m1	x	x	3.058	x	x	3.702	x
Spp1-Mm00436767_m1	1.443	1.577	1.431	0.571	0.861	1.451	1.401
Vdr-Mm00437297_m1	2.142	2.232	2.702	1.282	1.329	1.213	2.006
Rxra-Mm00441185_m1	1.021	0.65	0.768	1.065	1.128	1.31	1.271
Rara-Mm01296312_m1	1.249	1.47	1.302	1.326	1.434	0.916	1.088
Cyp27b1-Mm01165918_g1	1.024	4.959	1.61	0.604	1.446	0.765	0.994
Cyp24a1-Mm00487244_m1	2.361	2.57	2.603	0.287	x	0.975	x
Cd86-Mm00444543_m1	0.742	0.991	1.005	0.832	1.12	1.586	1.009
Cd163-Mm00474091_m1	1.387	0.787	2.268	1.022	0.553	0.959	1.131
Fosl1-Mm00487429_m1	1.518	3.101	2.001	0.35	0.973	1.394	0.742
Ets2-Mm00468977_m1	1.369	0.974	1.244	1.487	1.501	2.391	1.578
Sparc-Mm00486332_m1	1.639	2.094	2.028	0.264	0.675	3.915	0.7
Gas6-Mm00490378_m1	0.805	0.467	0.472	0.836	1.003	4.465	1.228
Fgf2-Mm01285715_m1	1.319	1.571	1.393	0.991	1.791	1.752	1.982
Wnt5a-Mm00437347_m1	2.121	2.358	2.916	1.216	2.277	3.735	2.787
Wnt5b-Mm00437350_m1	0.929	2.668	2.765	1.422	2.322	4.465	3.603
Ccr2-Mm00438270_m1	4.765	7.692	2.036	1.458	0.987	1.752	1.149

x – no detectable

Table S2. The expression of genes related to macrophage function and vitamin D mechanisms of action in differentiated BMDMs isolated from 4T1 and 67NR tumors.

	RQ							
	4T1				67NR			
Target genes:	1000+cal	5000	100	100+cal	1000+cal	5000	100	100+cal
Nos2-Mm00440502_m1	0.632	0.815	0.707	0.707	1.201	0.713	0.685	0.915
Runx1-Mm01213405_m1	0.696	0.833	0.808	0.853	0.984	1.106	0.877	0.996
Nfkb1-Mm00476361_m1	1.18	1.027	1.247	0.956	1	1.018	0.827	1.05
Irf8-Mm00492567_m1	1.026	1.02	1.224	0.977	1.03	1.076	0.919	1.07
Irf3-Mm00516784_m1	1.188	1.226	1.283	1.11	0.975	1.163	0.909	1.2
Irf4-Mm00516431_m1	3.673	2.859	0.816	0.771	1.012	0.831	1.518	1.638
Irf5-Mm00496477_m1	1.303	1.051	1.355	1.136	1.053	0.821	0.917	1.058
Tlr4-Mm00445273_m1	1.154	1.23	1.255	1.068	0.572	0.673	0.539	0.569
Stat1-Mm00439531_m1	1.306	0.885	1.334	0.796	0.976	0.796	0.759	0.963
Stat3-Mm01219775_m1	1.833	2.996	1.653	1.499	1.7	1.25	0.866	1.186
Stat6-Mm01160477_m1	0.896	1.016	1.2	1.058	1.085	1.047	0.88	1.041
Pparg-Mm01184322_m1	0.531	0.414	0.361	0.287	0.768	0.635	0.616	0.708
Cebpb-Mm00843434_s1	0.851	0.956	0.992	0.913	1.036	1.267	0.9	1.091
Mrc1-Mm01329362_m1	3.321	4.814	1.842	7.987	0.606	1.327	1.402	1.943
Ifng-Mm01168134_m1	0.48	x	0.72	0.473	x	x	x	x
Myd88-Mm00440338_m1	0.974	0.976	1.03	1.032	1.014	1.202	1.036	1.197
Ticam1-Mm00844508_s1	0.774	0.896	1.124	0.883	0.87	0.987	0.876	1.132
Vegfa-Mm00437306_m1	0.676	0.923	0.879	0.68	0.904	0.872	0.829	1.097
Pdgfa-Mm01205760_m1	1.589	0.834	1.594	1.104	0.757	1.043	0.741	0.721
Hgf-Mm01135193_m1	0.669	1.047	0.796	0.592	1.443	1.112	0.93	1.965

Egf-Mm00438696_m1	0.573	0.572	1.904	1.189	2.901	2.952	2.062	1.588
Alox15-Mm00507789_m1	x	x	x	x	0.835	0.765	0.794	x
Clec10a-Mm00546124_m1	0.937	1.09	1.424	1.082	0.865	1.045	0.942	0.869
Mgl2-Mm00460844_m1	x	x	x	x	x	1.315	x	0.646
Fn1-Mm01256744_m1	3.059	4.256	1.071	3.312	0.884	1.744	1.186	1.563
Tnf-Mm00443258_m1	0.779	0.636	1.086	0.639	0.96	1.07	0.831	0.883
Tgfb1-Mm01178820_m1	0.89	1.05	1.343	1.177	1.3	1.204	0.925	1.129
Il10-Mm01288386_m1	1.626	0.639	0.775	0.524	1.324	1.401	0.885	1.336
Il12a-Mm00434169_m1	0.721	1.002	0.947	1.181	1.059	1.575	1.045	1.109
Il12b-Mm00434174_m1	0.554	1.219	1.208	0.306	0.765	0.615	0.425	0.341
Il23a-Mm00518984_m1	15.833	11.68	1.851	14.443	x	x	x	x
Mmp1a-Mm00473485_m1	x	x	x	x	x	x	x	x
Mmp2-Mm00439506_m1	0.271	0.581	0.312	0.442	1.204	0.675	0.714	1.309
Mmp7-Mm01168420_m1	x	x	x	x	x	0.382	x	x
Mmp9-Mm00442991_m1	0.733	0.671	0.623	0.84	0.682	2.064	1.114	0.939
Mmp14-Mm00485054_m1	0.999	0.905	0.998	0.998	1.251	1.305	1.035	0.995
Ptges-Mm00452105_m1	0.689	0.768	0.825	0.744	1.078	1.199	0.946	1.074
Arg1-Mm00475988_m1	1.671	1.65	1.601	2.7	0.99	1.024	0.83	0.787
Irak3-Mm00518541_m1	1.007	0.96	1.055	1.112	1.059	1.116	0.865	0.918
Hif1a-Mm00468869_m1	0.888	1.065	1.182	1.099	0.924	1.075	0.933	0.965
Ccl1-Mm00441236_m1	x	x	x	x	x	x	x	x
Spp1-Mm00436767_m1	1.403	1.838	0.903	1.218	0.861	0.962	0.861	1.593
Vdr-Mm00437297_m1	1.936	1.896	0.465	1.55	2.712	1.708	1.34	2.15
Rxra-Mm00441185_m1	1.367	1.21	1.243	1.253	1.103	1.17	0.918	1.269
Rara-Mm01296312_m1	1.36	1.427	1.59	1.372	0.971	0.983	0.937	1.268
Cyp27b1-Mm01165918_g1	0.567	x	x	0.289	x	x	x	x
Cyp24a1-Mm00487244_m1	x	x	x	x	x	x	x	x
Cd86-Mm00444543_m1	0.715	1.063	0.868	0.794	1.071	0.852	1.054	1.256
Cd163-Mm00474091_m1	0.985	0.429	1.706	1.909	0.45	1.37	0.267	0.43
Fosl1-Mm00487429_m1	1.439	1.323	0.78	1.946	1.024	1.261	0.467	0.973
Ets2-Mm00468977_m1	0.521	0.81	0.823	0.859	0.984	1.087	1.059	1.643
Sparc-Mm00486332_m1	1.003	1.249	0.503	0.613	0.914	1.396	0.789	1.777
Gas6-Mm00490378_m1	1.244	1.075	1.165	0.96	1.162	1.172	1.11	1.367
Fgf2-Mm01285715_m1	x	x	x	x	1.254	0.911	1.087	2.15
Wnt5a-Mm00437347_m1	0.92	0.44	0.292	0.277	0.356	0.2	0.26	0.521
Wnt5b-Mm00437350_m1	0.579	1.177	x	0.383	x	0.943	2.089	x
Ccr2-Mm00438270_m1	0.765	0.899	1.098	0.916	3.327	0.963	0.972	0.819
Epcam-Mm00493214_m1	98.537	27.822	6.892	77.115	1.139	1.007	0.912	0.913

x – no detectable

References:

1. Porshneva K, Papiernik D, Psurski M, Łupicka-Słowik A, Matkowski R, Ekiert M, et al. Temporal inhibition of mouse mammary gland cancer metastasis by CORM-A1 and DETA/NO

combination therapy. Theranostics [Internet]. 2019 [cited 2019 Jun 25];9:3919–39. Available from: http://www.thno.org/v09p3918.htm
