# Supplementary material for: Calcitriol promotes M2 polarization of tumor-associated macrophages in 4T1 mouse mammary gland cancer via the induction of proinflammatory cytokines
Source: Sci Rep. 2024 Feb 15;14:3778. doi: 10.1038/s41598-024-54433-x (PMC10866890; doi:10.1038/s41598-024-54433-x)
Supplement: Supplementary file 2 — Supplementary Figures. [file 41598_2024_54433_MOESM2_ESM.pdf]

Supplementary information 2

Calcitriol promotes M2 polarization of tumor-associated macrophages in 4T1 mouse mammary gland cancer *via* the induction of proinflammatory cytokines

Martyna Stachowicz-Suhs[1], Natalia Łabędź[1], Artur Anisiewicz[1], Joanna Banach[1], Dagmara Kłopotowska[1], Magdalena Milczarek[1], Aleksandra Piotrowska[2], Piotr Dzięgiel[2], Adam Maciejczyk[3,4], Rafał Matkowski[3,4], Joanna Wietrzyk[1*]

[1]Department of Experimental Oncology, Hirszfeld Institute of Immunology and Experimental Therapy, Weigla 12, 53-114 Wroclaw, Poland; martyna.stachowicz@hirszfeld.pl, natalia.labedz@hirszfeld.pl, a.anisiewicz@captortherapeutics.com, joanna.banach@hirszfeld.pl, dagmara.klopotowska@hirszfeld.pl, magdalena.milczarek@hirszfeld.pl, joanna.wietrzyk@hirszfeld.pl

[2]Division of Histology and Embryology, Department of Human Morphology and Embryology, Faculty of Medicine, Wroclaw Medical University, Chałubińskiego 6a, 50-368 Wroclaw, Poland; aleksandra.piotrowska@umw.edu.pl, piotr.dziegiel@umw.edu.pl,

[3]Department of Oncology, Wroclaw Medical University, Pl. Ludwika Hirszfelda 12, 53-413 Wrocław, Poland; rafal.matkowski@umw.edu.pl, adam.maciejczyk@umw.edu.pl

[4]Lower Silesian Oncology, Pulmonology and Hematology Center, Pl. Ludwika Hirszfelda 12, 53-413 Wrocław, Poland; matkowski.rafal@dco.com.pl, adam.maciejczyk@dco.com.pl

*Correspondence: joanna.wietrzyk@hirszfeld.pl; Tel. +48713709985

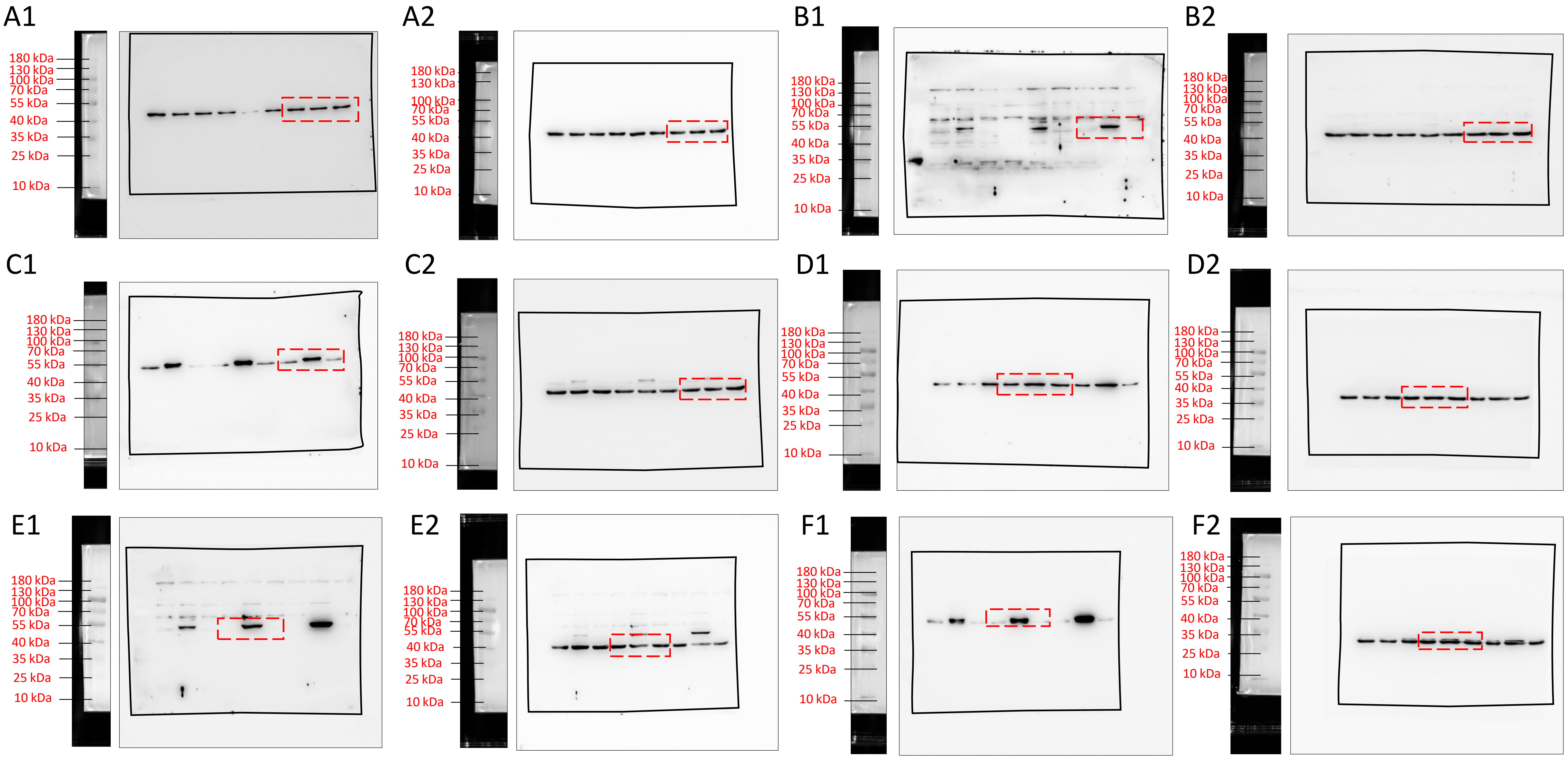

Figure S 1. **Uncropped western blot images corresponding to cropped blots presented in Figure 2 C in the manuscript**. A-C 4T1 cell line, D-F 67NR cell line. A1, D1 – CYP27B1; B1,E1 – CYP24A1; C1,F1 – VDR (vitamin D receptor); A2,B2,C2,D2,E2,F2 – loading control - β-actin. Chemiluminescence photographs with fragment of colorimetric picture showing protein ladder and the red box showing the cropping line. Visualization was performed on ChemiDoc Imaging System (Bio-Rad, Hercules, CA, USA) with exposure time estimated automatically.

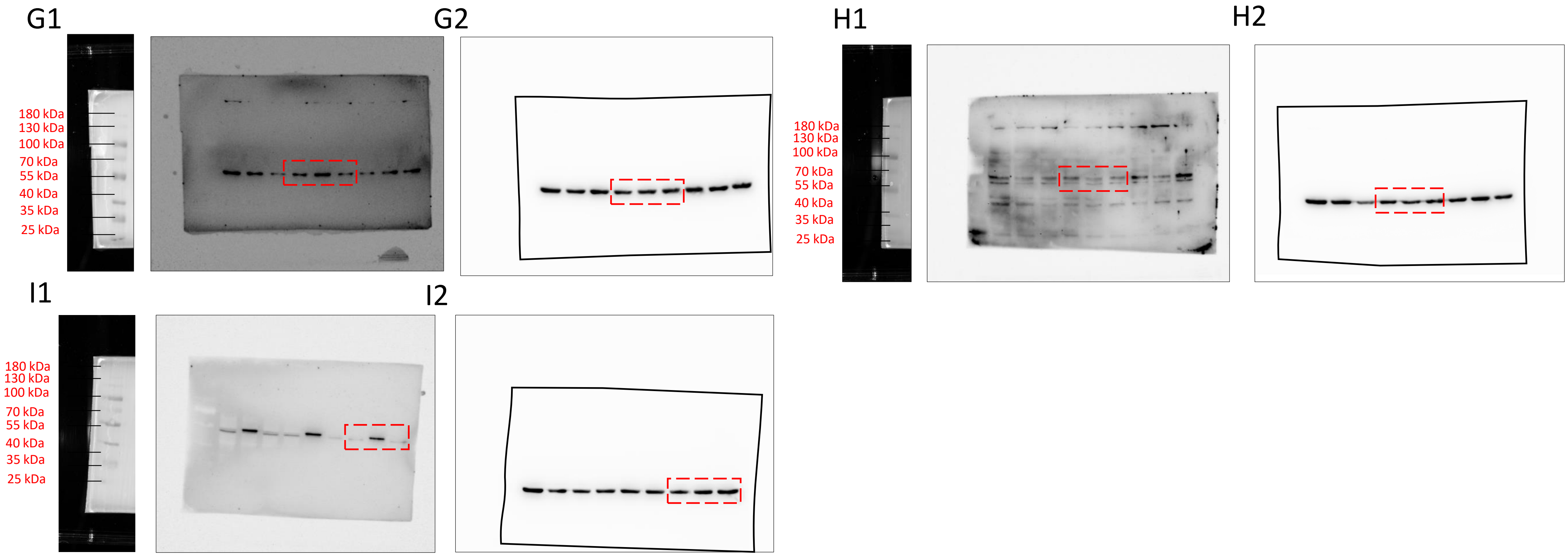

Figure S 1 c.d. **Uncropped western blot images corresponding to cropped blots presented in Figure 2 C in the manuscript**. G-I E0771cell line. G1 – CYP27B1; H1 – CYP24A1; I1 – VDR (vitamin D receptor); G2,H2,I2 – loading control - β-actin. Chemiluminescence photographs with fragment of colorimetric picture showing protein ladder and the red box showing the cropping line. Visualization was performed on ChemiDoc Imaging System (Bio-Rad, Hercules, CA, USA) with exposure time estimated automatically.

A1

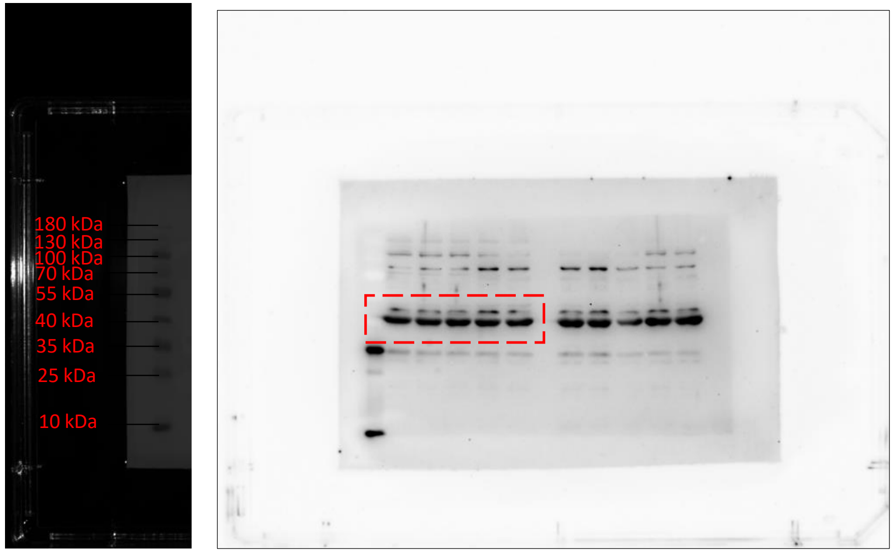

A2

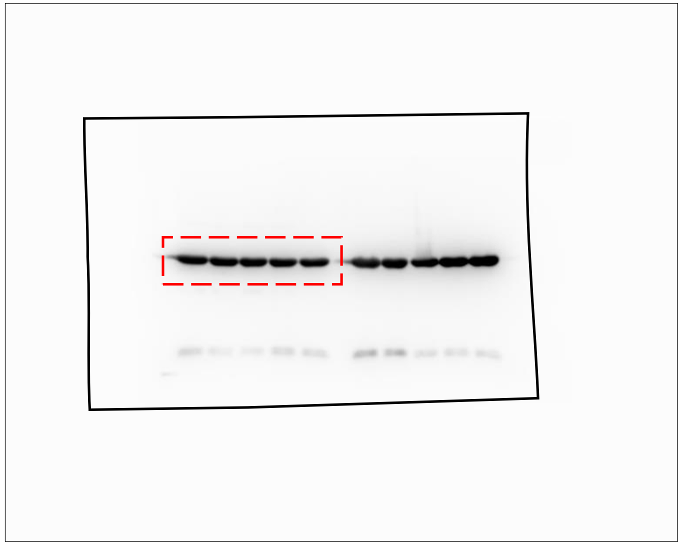

Figure S 2. **Uncropped blot images corresponding to cropped blots presented in Figure 4 E in the manuscript.** A1 – EpCAM, A2 – loading control – β-actin. Chemiluminescence photographs with fragment of colorimetric picture showing protein ladder and the red box showing the cropping line. Visualization was performed on ChemiDoc Imaging System (Bio-Rad, Hercules, CA, USA) with exposure time estimated automatically.

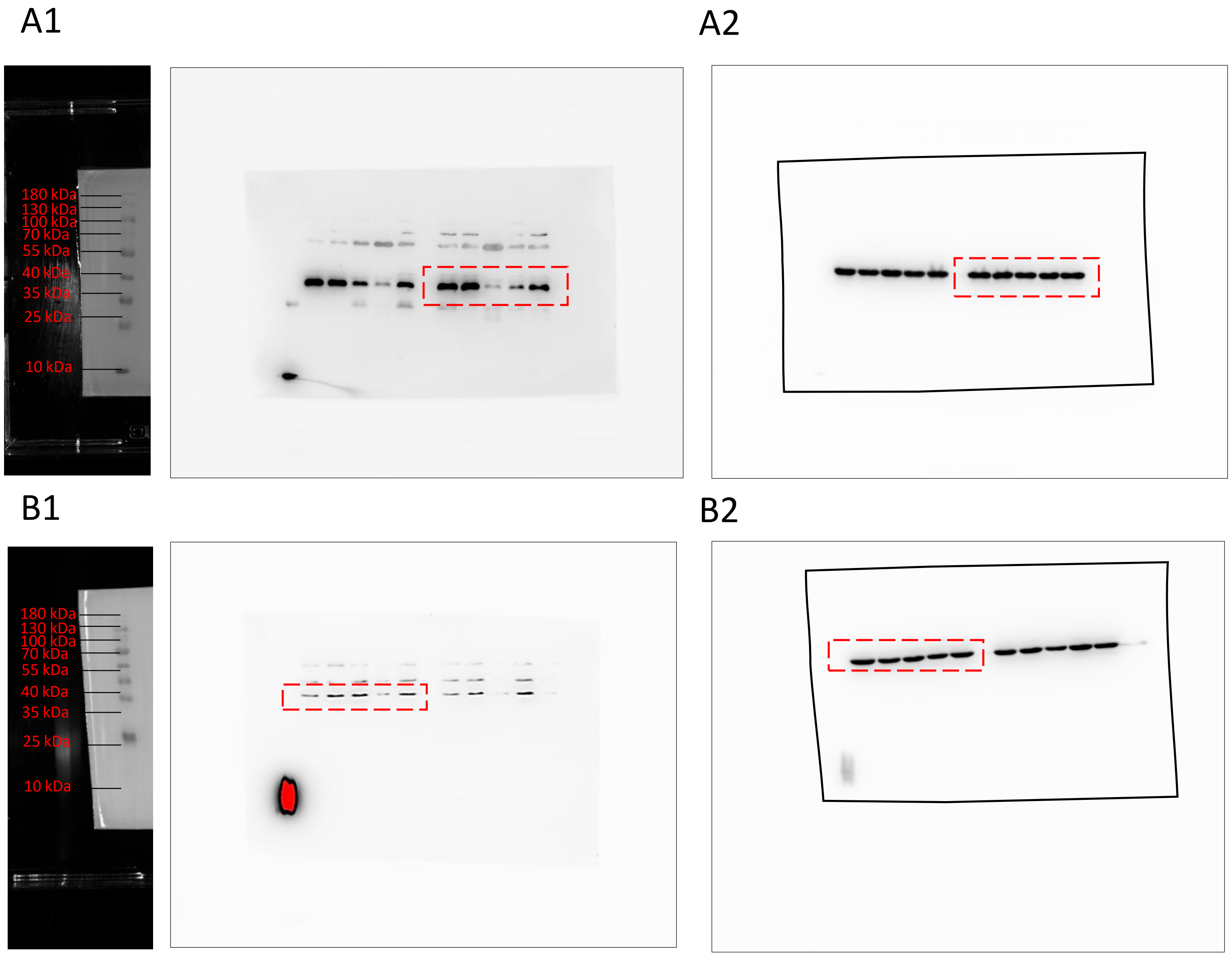

Figure S 3. **Uncropped blot images corresponding to cropped blots presented in Figure 5 E in the manuscript.** A1 – EpCAM; B1 – OPN (osteopontin). A2, B2 – loading control – β-actin. Chemiluminescence photographs with fragment of colorimetric picture showing protein ladder and the red box showing the cropping line. Visualization was performed on ChemiDoc Imaging System (Bio-Rad, Hercules, CA, USA) with exposure time estimated automatically.

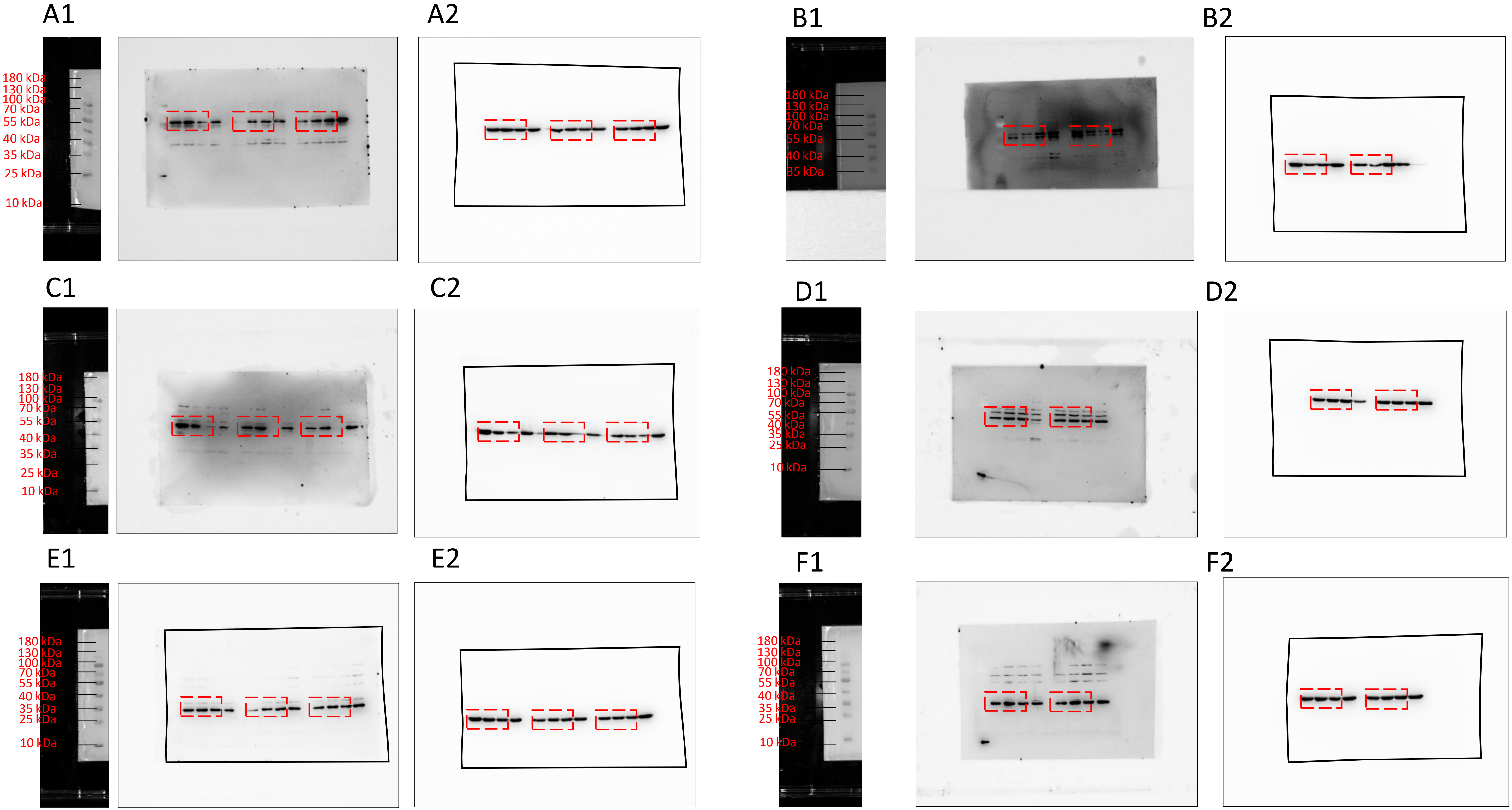

Figure S 4. **Uncropped blot images corresponding to cropped blots presented in Figure 7 E in the manuscript.** A1, B1 – IRF4; C1,D1 – MMP3; E1, F1 – TGFβ. A2, B2, C2, D2, E2, F2 – loading control – β-actin. Chemiluminescence photographs with fragment of colorimetric picture showing protein ladder and the red box showing the cropping line. Visualization was performed on ChemiDoc Imaging System (Bio-Rad, Hercules, CA, USA) with exposure time estimated automatically.

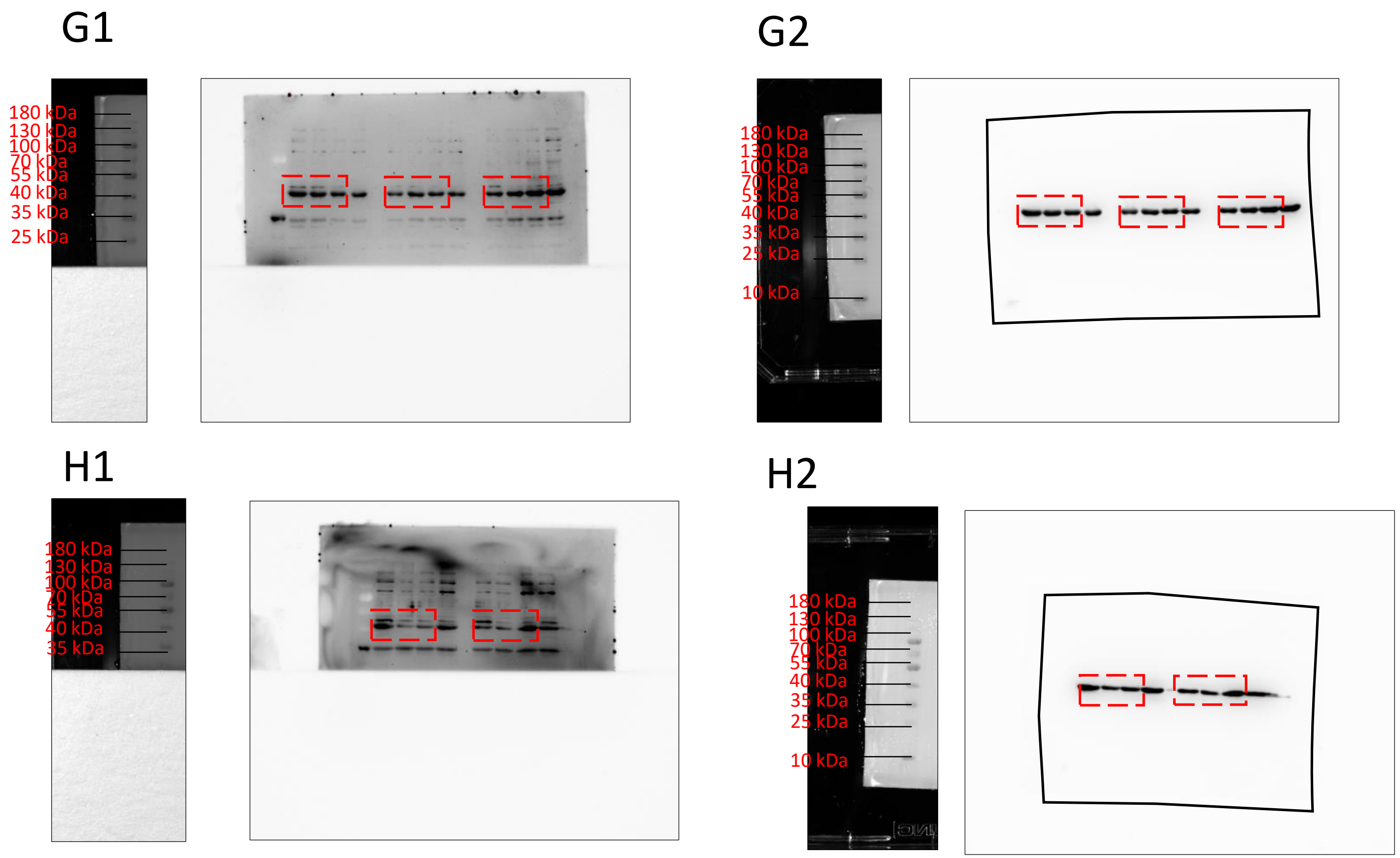

Figure S 4 c.d. **Uncropped blot images corresponding to cropped blots presented in Figure 7 E in the manuscript. G1,H1 – EpCAM; G2, H2** loading control – β-actin. Chemiluminescence photographs with fragment of colorimetric picture showing protein ladder and the red box showing the cropping line. Visualization was performed on ChemiDoc Imaging System (Bio-Rad, Hercules, CA, USA) with exposure time estimated automatically.

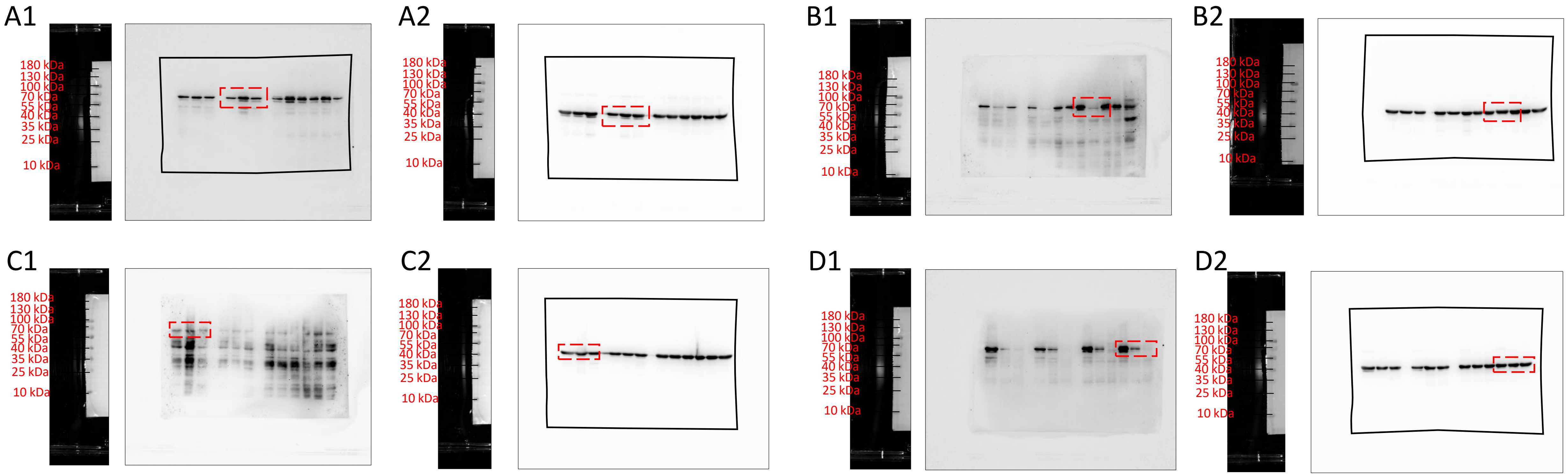

Figure S 5. **Uncropped blot images corresponding to cropped blots presented in Figure 8 C (A-C) and D (D) in the manuscript.** A – 4T1 cell line, B – 67NR cell line, C – E0771 cell line, D – comparison of all tested cell lines. A1, B1, C1, D1 – COX2 (cyclooxygenase 2); A2, B2, C2, D2 – loading control- β-actin. Chemiluminescence photographs with fragment of colorimetric picture showing protein ladder and the red box showing the cropping line. Visualization was performed on ChemiDoc Imaging System (Bio-Rad, Hercules, CA, USA) with exposure time estimated automatically.

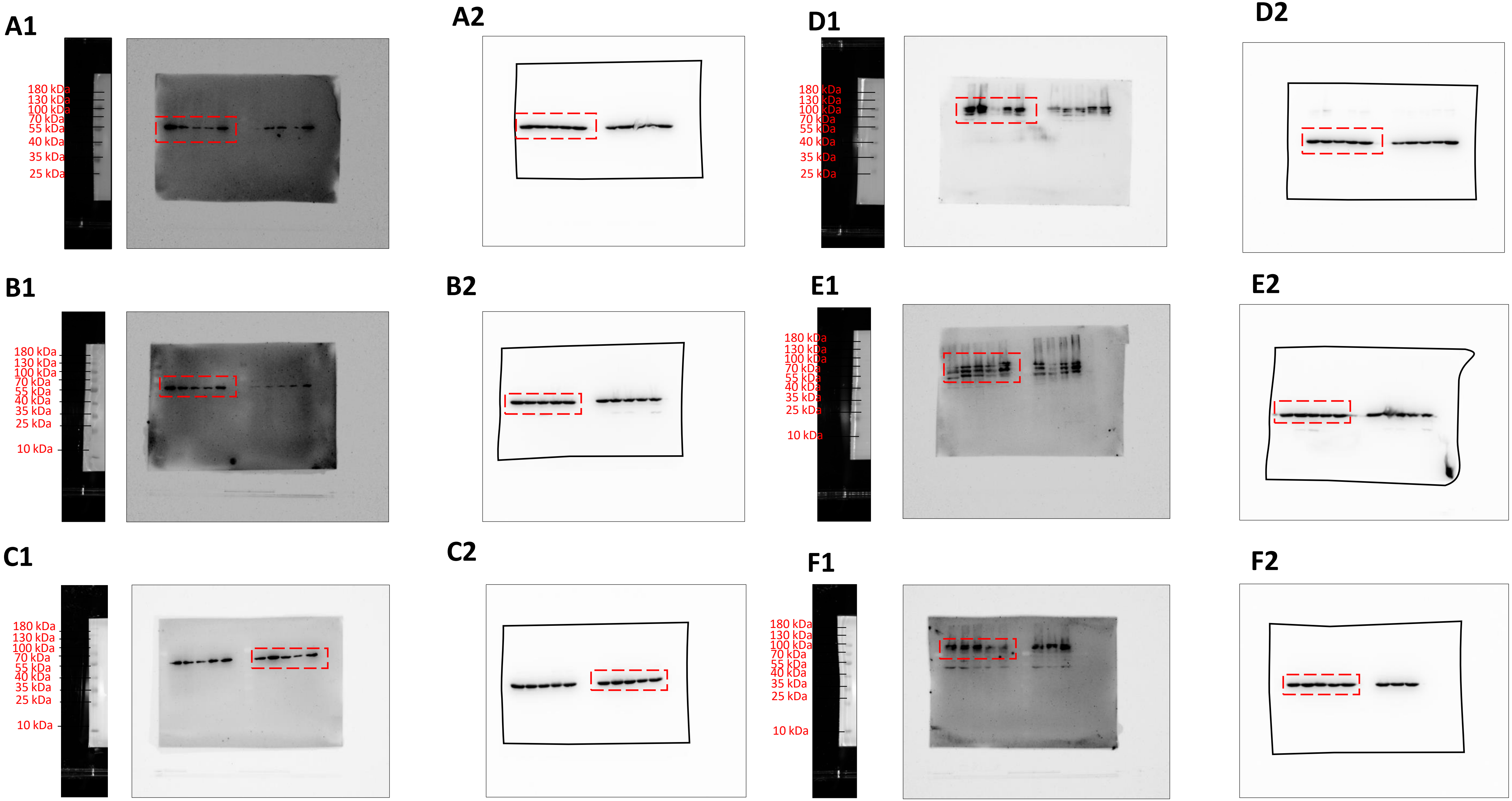

Figure S 6. **Uncropped blot images corresponding to cropped blots presented in Figure S 1 B in the supplementary file 1. A1, B1, C1 - PDIA3; D1, E1, F1 - β-catenin. A2, B2, C2, D2, E2, F2 – loading control – β-actin. A,D - 4T1, B,E - 67NR, C, F - E0771.** Chemiluminescence photographs with fragment of colorimetric picture showing protein ladder and the red box showing the cropping line. Visualization was performed on ChemiDoc Imaging System (Bio-Rad, Hercules, CA, USA) with exposure time estimated automatically.

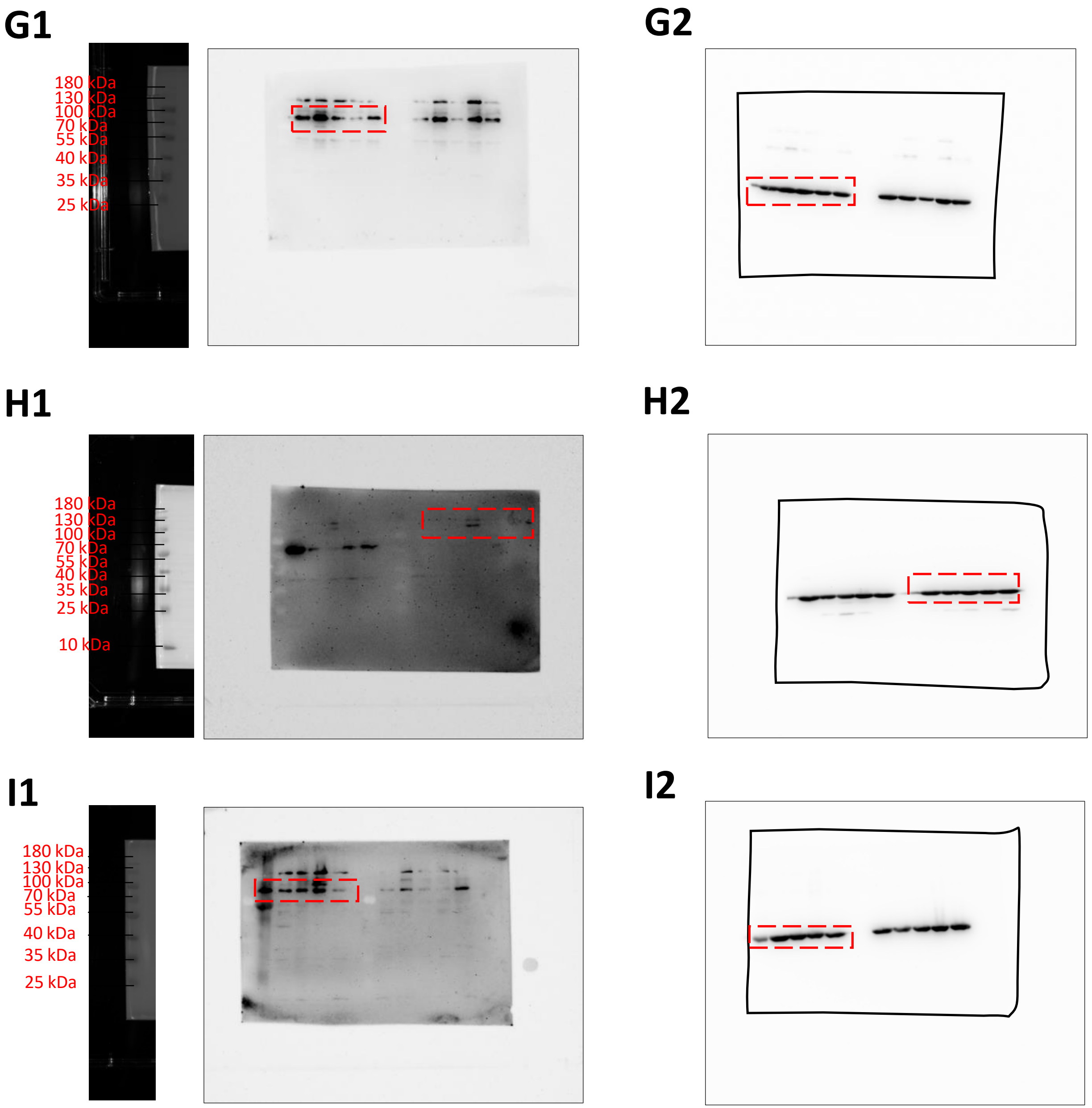

Figure S 6 c.d.. **Uncropped blot images corresponding to cropped blots presented in Figure S 1 B in the supplementary file 1. G1, H1, I1 - E-cadherin.; G2, H2, I2 - loading control – β-actin. G - 4T1, H - 67NR, I - E0771**. Chemiluminescence photographs with fragment of colorimetric picture showing protein ladder and the red box showing the cropping line. Visualization was performed on ChemiDoc Imaging System (Bio-Rad, Hercules, CA, USA) with exposure time estimated automatically.

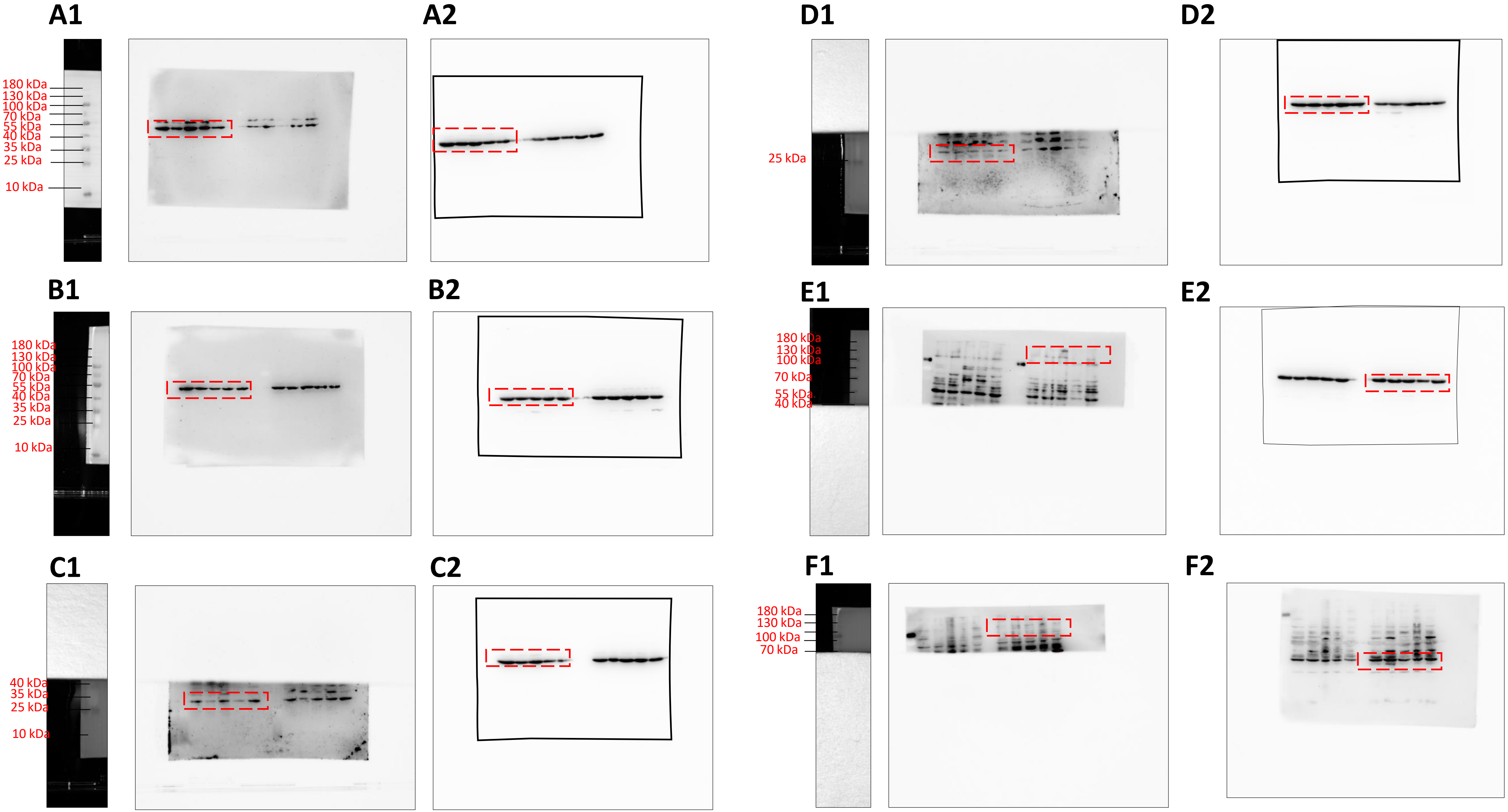

Figure S 6. **Uncropped blot images corresponding to cropped blots presented in Figure S 1 C in the supplementary file 1.** A1, B1 - MMP3; C1, D1 – PTHLH; E1, F1 – DIS3. A2, B2, C2, D2, E2, F2 - loading control – β-actin. A,C, E - 4T1; B, D, F - 67NR. Chemiluminescence photographs with fragment of colorimetric picture showing protein ladder and the red box showing the cropping line. Visualization was performed on ChemiDoc Imaging System (Bio-Rad, Hercules, CA, USA) with exposure time estimated automatically.

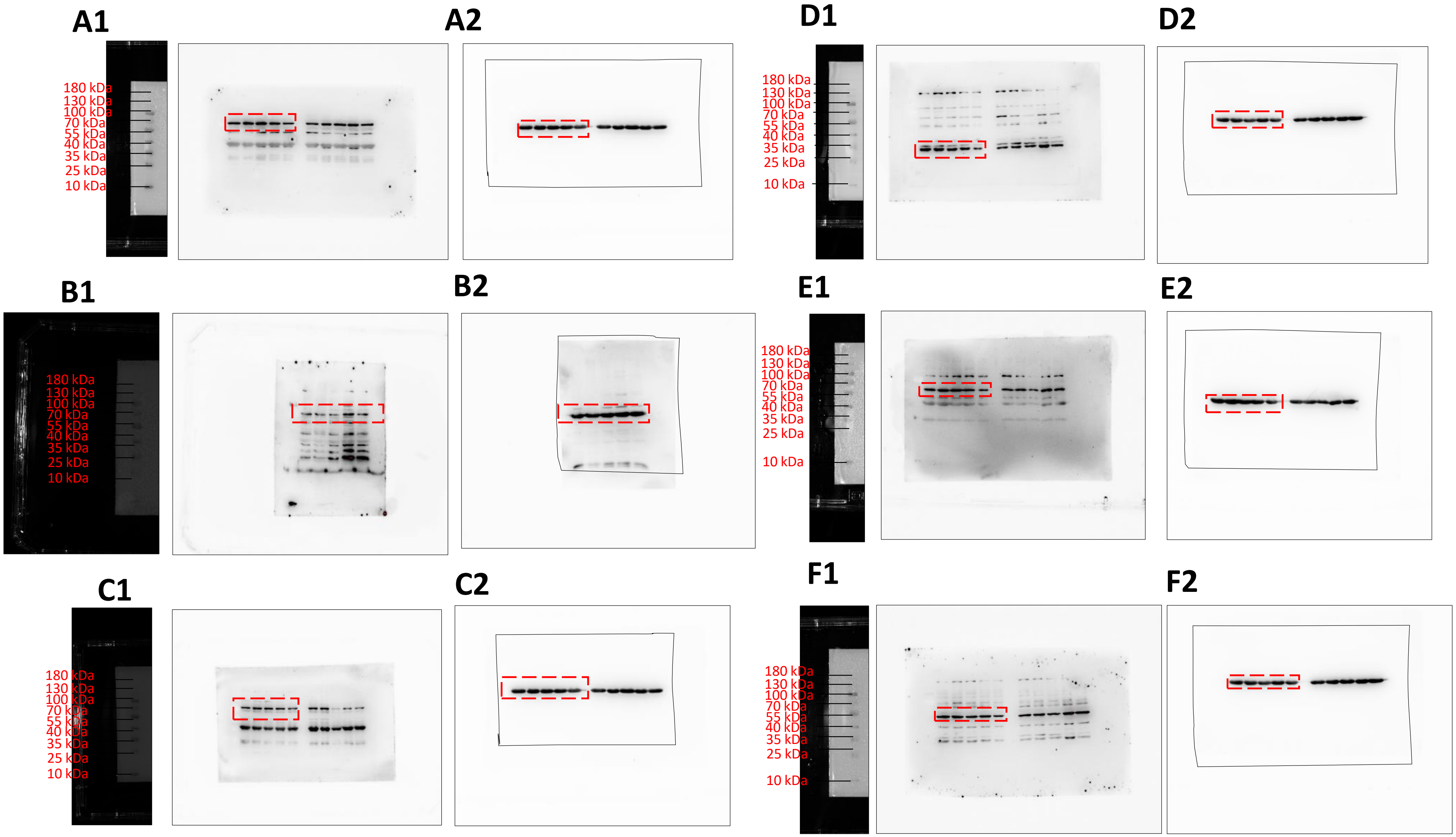

Figure S 7. **Uncropped blot images corresponding to cropped blots presented in Figure S 5 B in the supplementary file 1.** A1. CYP27B1, B1 VDR, C1 OPN, D1 TGFβ, E1 PDIA3, F1 MMP3; A2, B2, C2, D2, E2, F2 - loading control – β-actin. A,C, E - 4T1; B, D, F - 67NR. Chemiluminescence photographs with fragment of colorimetric picture showing protein ladder and the red box showing the cropping line. Visualization was performed on ChemiDoc Imaging System (Bio-Rad, Hercules, CA, USA) with exposure time estimated automatically.

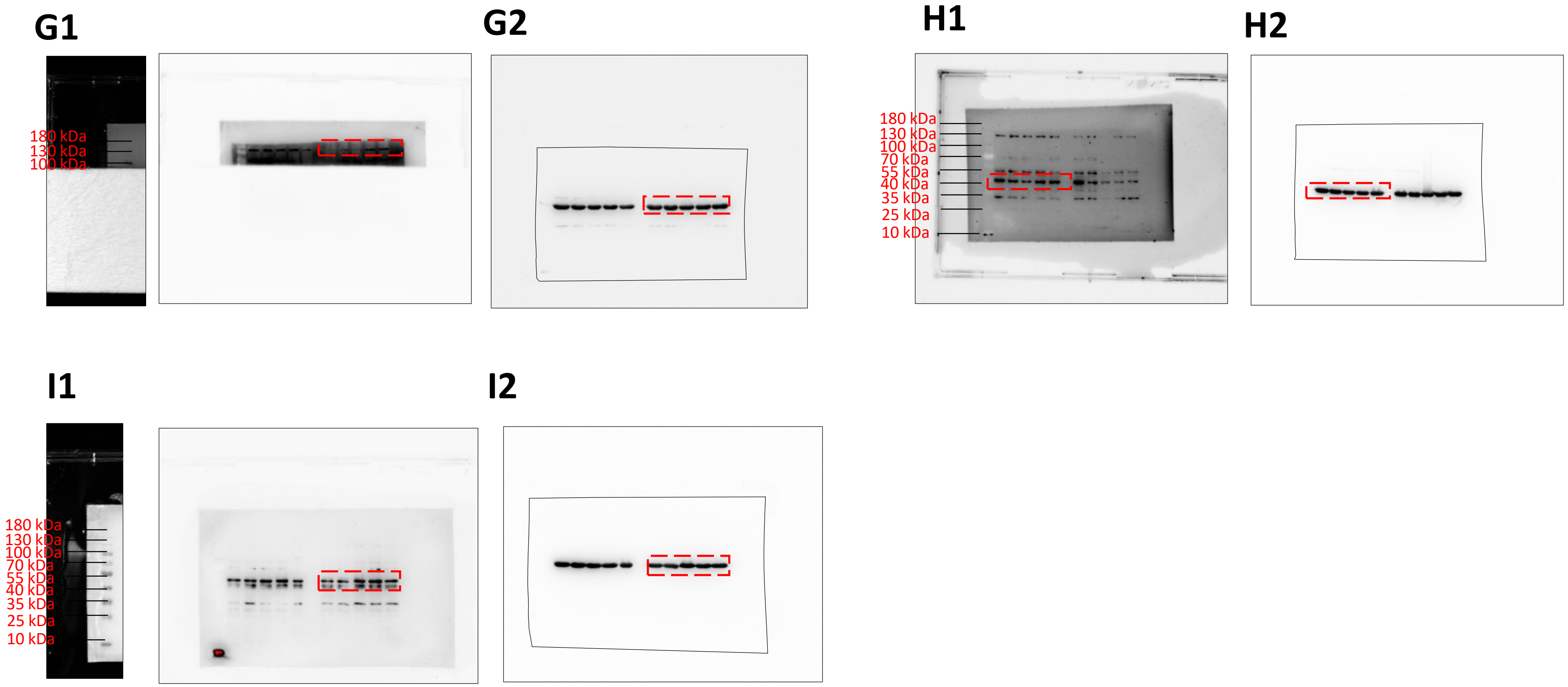

Figure S 7. **Uncropped blot images corresponding to cropped blots presented in Figure S 5 B in the supplementary file 1.** G1 Zeb1, H1 AP-1, I1 IRF4; G2, H2, I2 - loading control – β-actin. Chemiluminescence photographs with fragment of colorimetric picture showing protein ladder and the red box showing the cropping line. Visualization was performed on ChemiDoc Imaging System (Bio-Rad, Hercules, CA, USA) with exposure time estimated automatically.

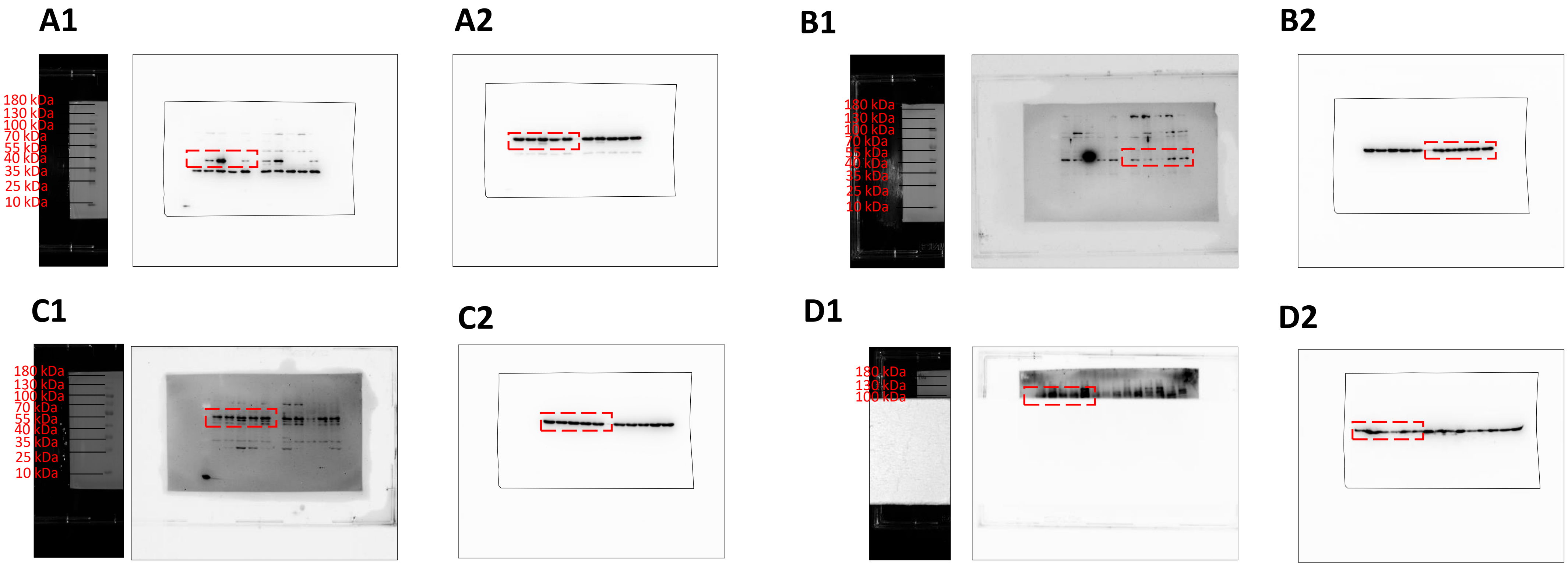

Figure S 7. **Uncropped blot images corresponding to cropped blots presented in Figure S 5 C in the supplementary file 1.** A1 TGFβ, B1, AP-1, C1 IRF4, D1 Zeb-1; A2,B2,C2,D2 -- loading control – β-actin. Chemiluminescence photographs with fragment of colorimetric picture showing protein ladder and the red box showing the cropping line. Visualization was performed on ChemiDoc Imaging System (Bio-Rad, Hercules, CA, USA) with exposure time estimated automatically.

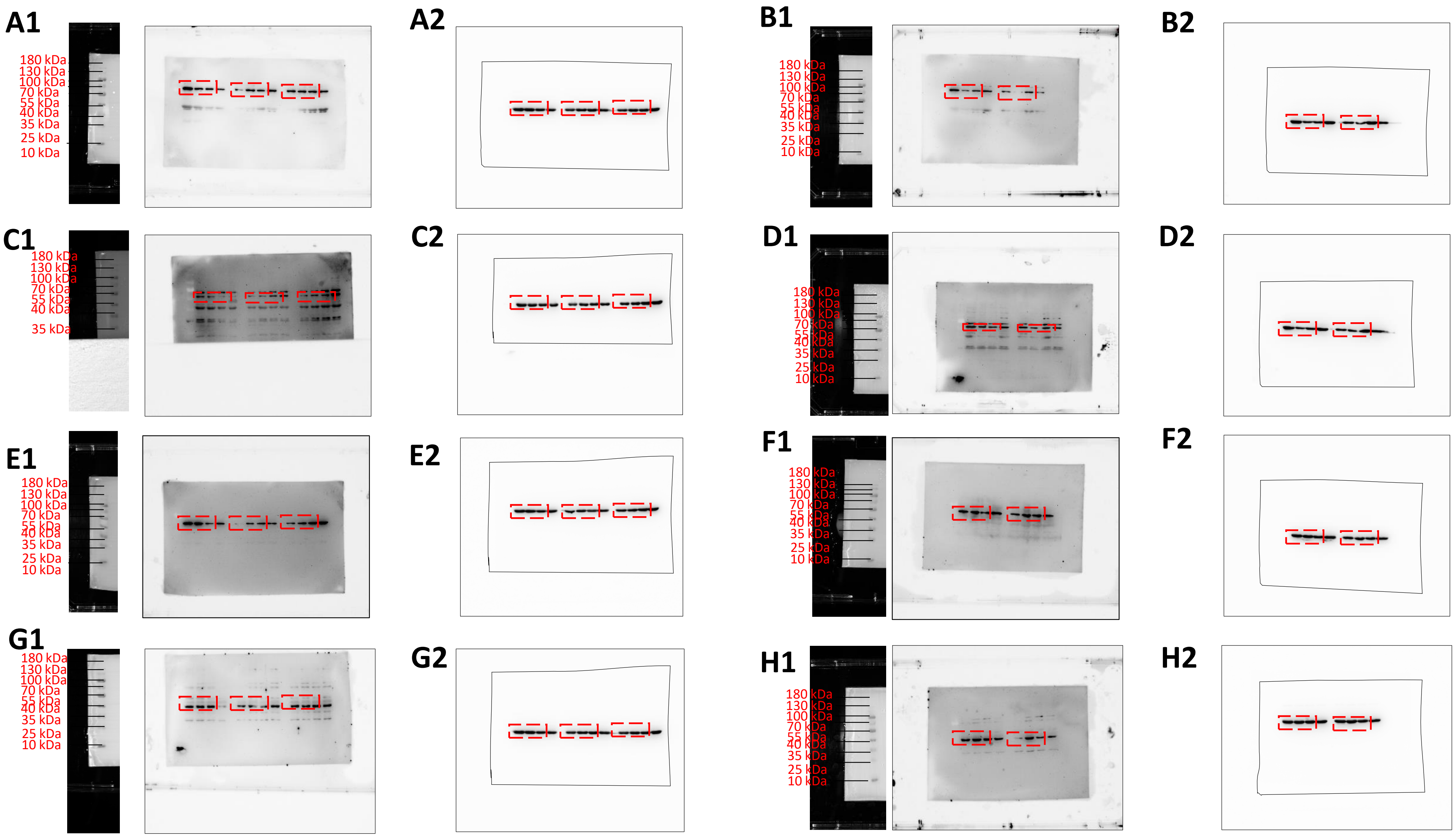

Figure S 7. **Uncropped blot images corresponding to cropped blots presented in Figure S 6 I-L in the supplementary file 1. A1, B1 OPN, C1, D1 CYP27B1, E1, F1 PDIA3, G1, H1 AP-1. A2, B2, C2, D2, E2, F2, G2, H2 -** loading control – β-actin. Chemiluminescence photographs with fragment of colorimetric picture showing protein ladder and the red box showing the cropping line. Visualization was performed on ChemiDoc Imaging System (Bio-Rad, Hercules, CA, USA) with exposure time estimated automatically.

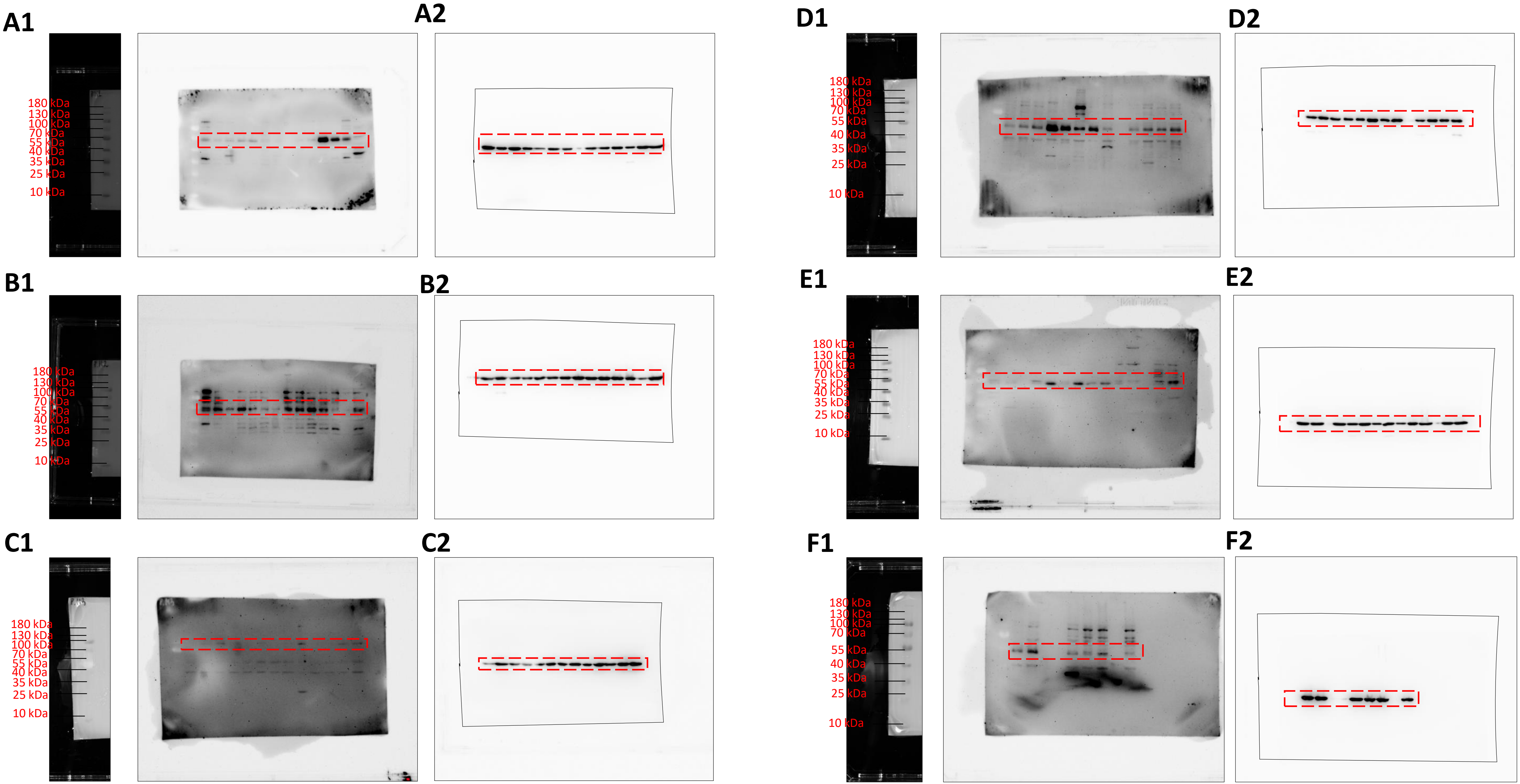

Figure S 8. **Uncropped blot images corresponding to cropped blots presented in the order shown in Figure S 9C in the supplementary file 1. A1, B1, C1, D1, E1, F1 – COX-2. A2, B2, C2, D2, E2, F2-** loading control – β-actin. Chemiluminescence photographs with fragment of colorimetric picture showing protein ladder and the red box showing the cropping line. Visualization was performed on ChemiDoc Imaging System (Bio-Rad, Hercules, CA, USA) with exposure time estimated automatically.

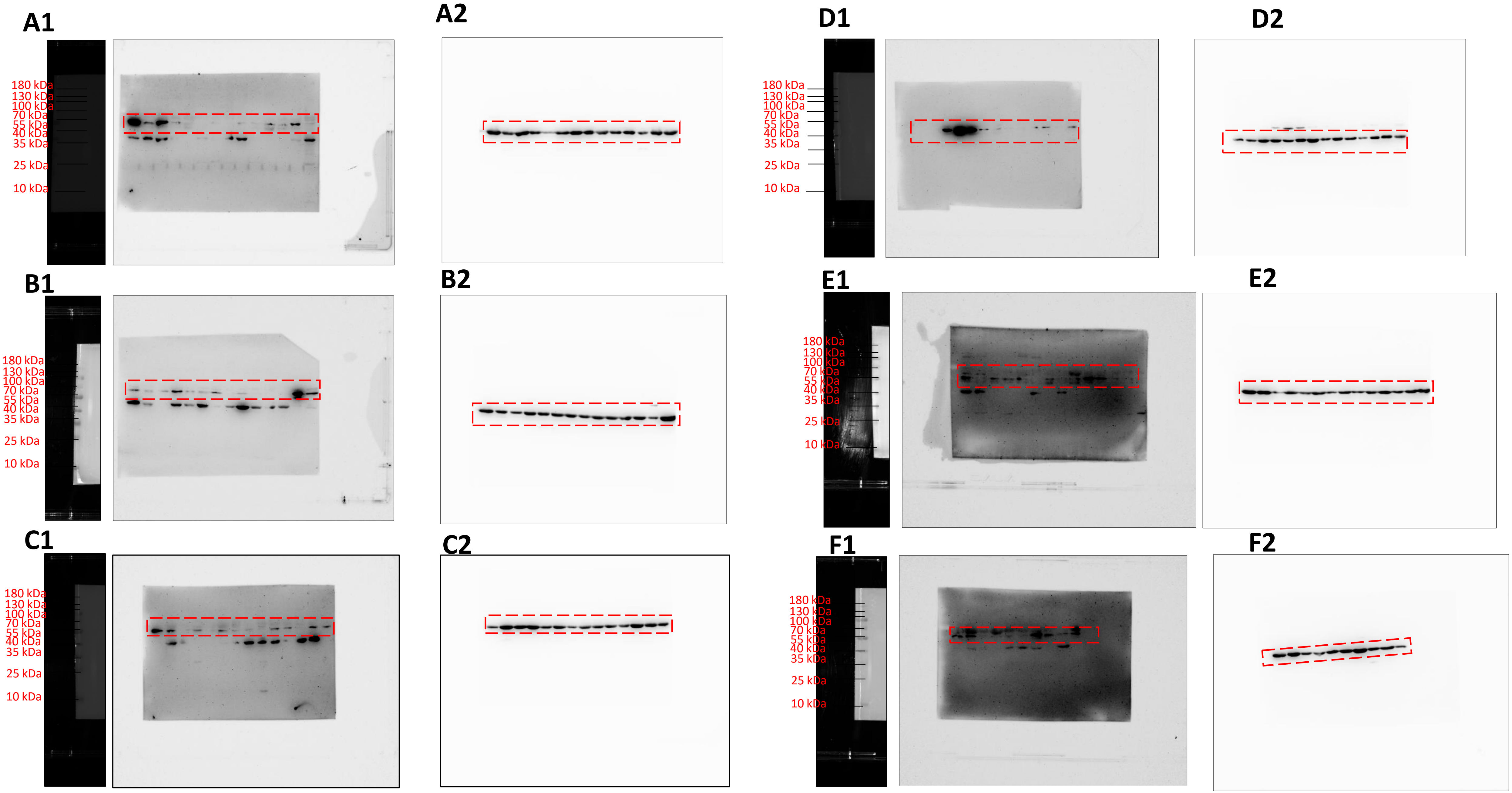

Figure S 9. **Uncropped blot images corresponding to cropped blots presented in the order shown in Figure S 9C in the supplementary file 1. A1, B1, C1, D1, E1, F1 – VDR. A2, B2, C2, D2, E2, F2-** loading control – β-actin. Chemiluminescence photographs with fragment of colorimetric picture showing protein ladder and the red box showing the cropping line. Visualization was performed on ChemiDoc Imaging System (Bio-Rad, Hercules, CA, USA) with exposure time estimated automatically.

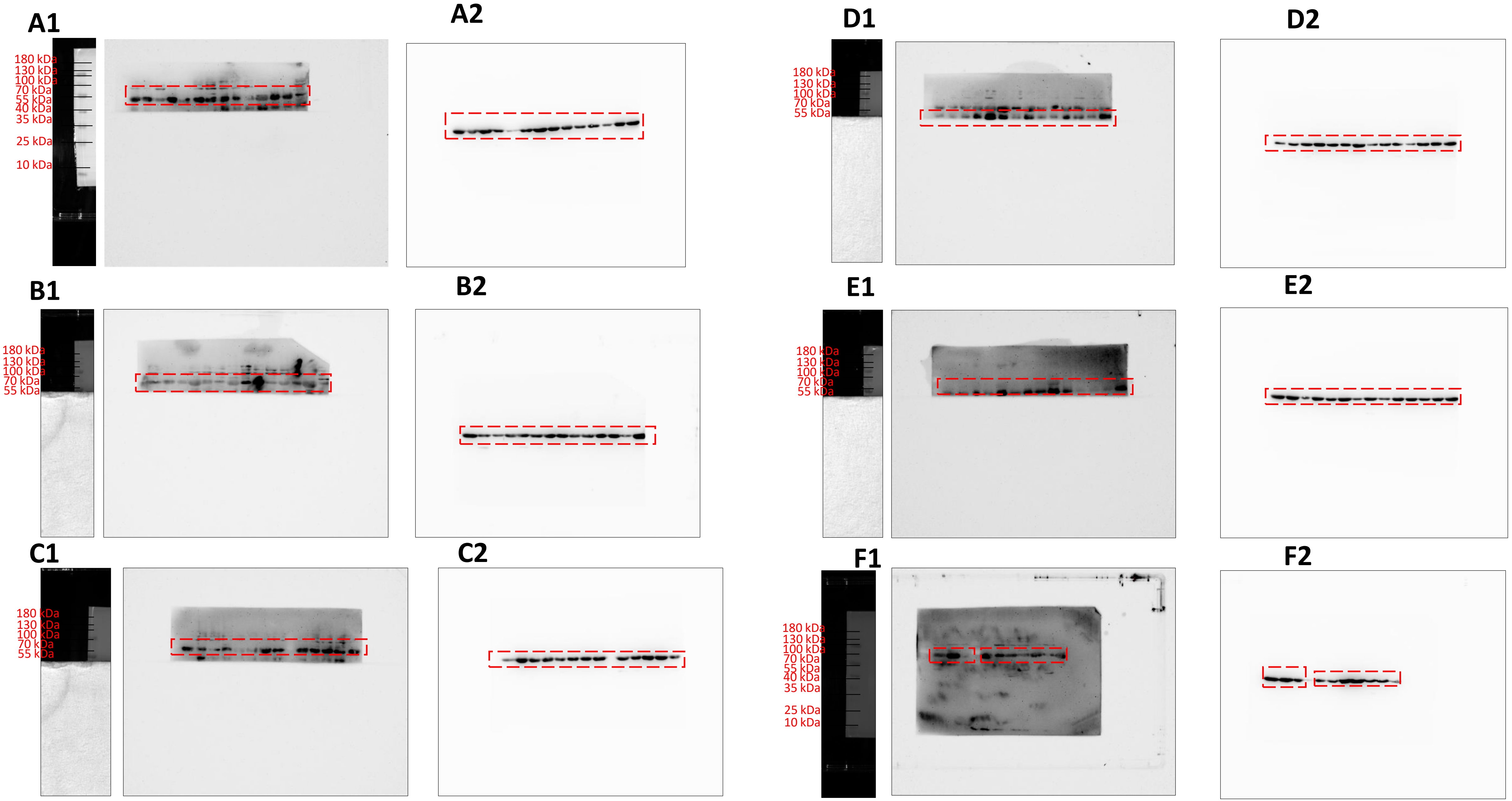

Figure S 10. **Uncropped blot images corresponding to cropped blots presented in the order shown in Figure S 9C in the supplementary file 1. A1, B1, C1, D1, E1, F1 – CYP24A1. A2, B2, C2, D2, E2, F2-** loading control – β-actin. Chemiluminescence photographs with fragment of colorimetric picture showing protein ladder and the red box showing the cropping line. Visualization was performed on ChemiDoc Imaging System (Bio-Rad, Hercules, CA, USA) with exposure time estimated automatically.

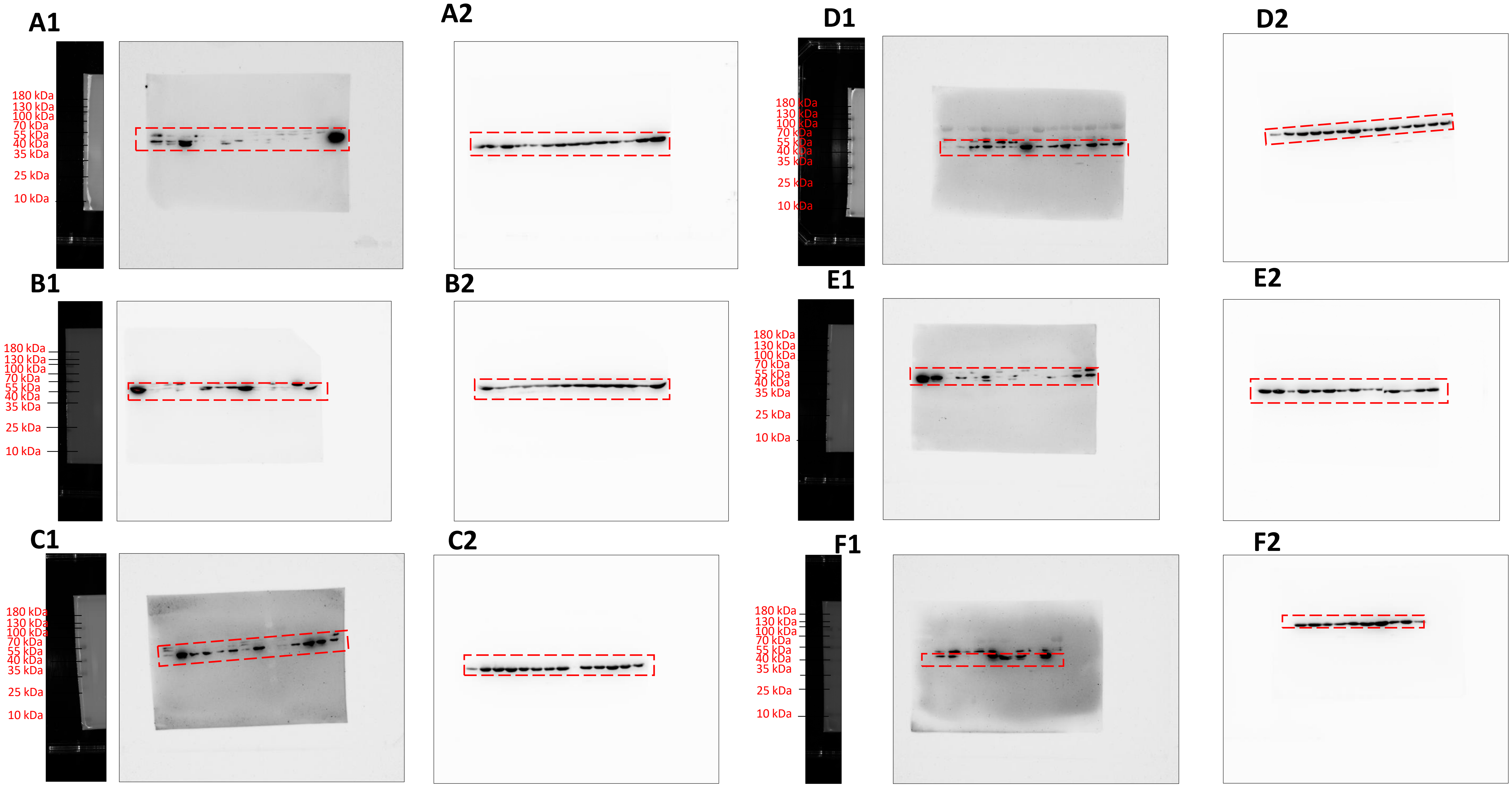

Figure S 11. **Uncropped blot images corresponding to cropped blots presented in the order shown in Figure S 9C in the supplementary file 1. A1, B1, C1, D1, E1, F1 – CYP27B1. A2, B2, C2, D2, E2, F2-** loading control – β-actin. Chemiluminescence photographs with fragment of colorimetric picture showing protein ladder and the red box showing the cropping line. Visualization was performed on ChemiDoc Imaging System (Bio-Rad, Hercules, CA, USA) with exposure time estimated automatically.
